# A1 is induced by pathogen ligands to limit myeloid cell death and NLRP3 inflammasome activation

**DOI:** 10.15252/embr.202356865

**Published:** 2023-10-17

**Authors:** Mary Speir, Hazel Tye, Timothy A Gottschalk, Daniel S Simpson, Tirta M Djajawi, Pankaj Deo, Rebecca L Ambrose, Stephanie A Conos, Jack Emery, Gilu Abraham, Ashlyn Pascoe, Sebastian A Hughes, Ashley Weir, Edwin D Hawkins, Isabella Kong, Marco J Herold, Jaclyn S Pearson, Najoua Lalaoui, Thomas Naderer, James E Vince, Kate E Lawlor

**Affiliations:** ^1^ Centre for Innate Immunity and Infectious Diseases Hudson Institute of Medical Research Clayton VIC Australia; ^2^ Department of Molecular and Translational Science Monash University Clayton VIC Australia; ^3^ The Walter and Eliza Hall Institute of Medical Research Parkville VIC Australia; ^4^ Department of Medical Biology University of Melbourne Parkville VIC Australia; ^5^ Department of Biochemistry and Molecular Biology, Monash Biomedicine Discovery Institute Monash University Clayton VIC Australia; ^6^ Department of Microbiology Monash University Clayton VIC Australia

**Keywords:** BCL‐2A1, mitochondrial apoptosis, myeloid cells, NLRP3, TLR ligation, Autophagy & Cell Death, Immunology, Signal Transduction

## Abstract

Programmed cell death pathways play an important role in innate immune responses to infection. Activation of intrinsic apoptosis promotes infected cell clearance; however, comparatively little is known about how this mode of cell death is regulated during infections and whether it can induce inflammation. Here, we identify that the pro‐survival BCL‐2 family member, A1, controls activation of the essential intrinsic apoptotic effectors BAX/BAK in macrophages and monocytes following bacterial lipopolysaccharide (LPS) sensing. We show that, due to its tight transcriptional and post‐translational regulation, A1 acts as a molecular rheostat to regulate BAX/BAK‐dependent apoptosis and the subsequent NLRP3 inflammasome‐dependent and inflammasome‐independent maturation of the inflammatory cytokine IL‐1β. Furthermore, induction of A1 expression in inflammatory monocytes limits cell death modalities and IL‐1β activation triggered by *Neisseria gonorrhoeae*‐derived outer membrane vesicles (NOMVs). Consequently, A1‐deficient mice exhibit heightened IL‐1β production in response to NOMV injection. These findings reveal that bacteria can induce A1 expression to delay myeloid cell death and inflammatory responses, which has implications for the development of host‐directed antimicrobial therapeutics.

## Graphical abstract

Cell death and inflammatory signalling crosstalk is critical for innate immune responses to pathogens. This study identifies the BCL‐2 family member A1 as a temporal regulator of these processes in myeloid cells upon bacterial recognition.

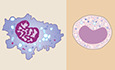

## Introduction

The programmed death of innate immune cells can be deployed to combat infection. In the case of bacteria, the cytosolic NOD‐like receptor (NLR) and absent in melanoma 2 (AIM2) sensing of specific bacterial ligands, or the activity of effector molecules, leads to the assembly and activation of multimeric signalling platforms called inflammasomes. Inflammasomes, such as the NLRP3 inflammasome, activate caspase‐1 to mature the pro‐inflammatory cytokine, interleukin (IL)‐1β, as well as to cleave and activate the pore‐forming protein Gasdermin D (GSDMD) that can induce a lytic form of cell death called pyroptosis. GSDMD pores facilitate the release of inflammatory cytokines and damage‐associated molecular patterns (DAMPs) that alert the host immune system, while pyroptosis restricts intracellular bacterial replication (Broz & Dixit, 2016). Caspase‐11‐sensing of the lipid A moiety of Gram‐negative bacteria‐derived LPS also results in GSDMD‐mediated pyroptosis that promotes NLRP3 inflammasome activation via potassium ion (K^+^) efflux (Kayagaki *et al*, 2015; Ruhl & Broz, 2015; Shi *et al*, 2015). While programmed cell death can protect mammals from infection, it can also limit the ability of phagocytes to eliminate pathogens in a cell‐autonomous manner. Consistent with this, many pathogens have evolved virulence factors that manipulate host cell death machinery in order to evade antimicrobial responses (Cunha & Zamboni, 2013; Naderer & Fulcher, 2018; Eng *et al*, 2020). This includes intracellular pathogens (e.g., *Legionella*) that evade detection by cytosolic sensors and delay macrophage cell death in order to establish a replicative niche (Speir *et al*, 2016).

Intrinsic, or mitochondrial, apoptosis plays a key role in the non‐immunogenic phagocytic clearance of damaged, superfluous, or infected cells (Jorgensen *et al*, 2017). Permeabilisation of the mitochondrial outer membrane by the essential apoptotic effectors BAX and BAK facilitates cytochrome‐*c* release that nucleates the APAF‐1/caspase‐9 apoptosome to activate the executioner caspases, caspase‐3 and caspase‐7. In healthy cells, BAX/BAK activity is restrained by members of the pro‐survival B cell lymphoma‐2 (BCL‐2) family, including BCL‐2, BCL‐XL, BCL‐W, MCL‐1, and BCL2A1 (A1; BFL‐1 in humans), which are themselves antagonised by the actions of the pro‐apoptotic BH3‐only proteins (Singh *et al*, 2019). Based on the fact that pro‐survival BCL‐2 proteins are frequently linked with cancer progression and chemotherapy resistance, a number of highly specific BH3‐mimetic compounds have been developed, including pan‐BCL‐2‐targeting ABT‐737/ABT‐263 (Oltersdorf *et al*, 2005; van Delft *et al*, 2006; Tse *et al*, 2008), MCL‐1‐targeting S63845 (Kotschy *et al*, 2016), and BCL‐2‐targeting ABT‐199 (Souers *et al*, 2013). While these drugs show broad clinical efficacy in cancer (Lee & Fairlie, 2021), their therapeutic potential in infectious disease is yet to be fully explored (Ohmer *et al*, 2016; Speir *et al*, 2016; Bulanova *et al*, 2017; Suzuki *et al*, 2018; Inde *et al*, 2021).

While mitochondrial apoptosis is generally considered immunologically silent, recent reports have suggested that apoptotic factors and/or mitochondria promote NLRP3 inflammasome activity. In fact, several models propose that mitochondrial damage is essential for NLRP3 inflammasome activation and assembly in response to canonical stimuli, such as the bacterial toxin nigericin or ATP (reviewed in Lawlor & Vince, 2014; Yabal *et al*, 2019; Riley & Tait, 2020). However, a number of these models have now been challenged (Allam *et al*, 2014; Dang *et al*, 2017). For instance, it was proposed that canonical NLRP3 triggers universally induce mitochondrial stress and the release of oxidised mitochondrial DNA (mtDNA) via the BAX/BAK pore or the mitochondrial Permeability Transition Pore (mPTP) to activate NLRP3 (Shimada *et al*, 2012; Xian *et al*, 2022). Yet, even the original report by Shimada *et al* (2012) actually showed that the majority of the inflammasome response was dependent on the DNA sensor AIM2. Other recent studies have further contradicted these models by revealing that mtDNA depletion does not impact NLRP3 responses to ATP (Dang *et al*, 2017) and by showing genetically that BAX/BAK, caspase‐9, and the mPTP component cyclophilin‐D are not required for canonical NLRP3 activation (Allam *et al*, 2014; Vince *et al*, 2018). Despite debate surrounding the actual contribution of the mitochondria to canonical NLRP3 inflammasome activity, a number of studies have revealed that both intrinsic and extrinsic apoptotic cell death modalities can crosstalk with inflammatory signalling pathways, particularly the NLRP3 inflammasome (Lawlor *et al*, 2015; Conos *et al*, 2017; Malireddi *et al*, 2018; Vince *et al*, 2018). In the case of intrinsic apoptosis, BAX/BAK signalling in LPS‐primed macrophages has been shown to trigger caspase‐3/‐7‐dependent NLRP3 inflammasome activation, as well as caspase‐8‐mediated IL‐1β cleavage (Chauhan *et al*, 2018; Vince *et al*, 2018). It has also become apparent that IL‐1β and other inflammatory DAMPs released by apoptotic cells can activate the immune system during infection, akin to pyroptosis (Chauhan *et al*, 2018; Deo *et al*, 2020; Orzalli *et al*, 2021). However, further work is needed to delineate how BAX/BAK activation is regulated during infection and whether these signals can fine‐tune cell death and inflammatory responses in diverse innate immune cells.

Here, we investigate how bacterial sensing alters the ability of myeloid cells to induce mitochondrial apoptosis and inflammation. We show that pro‐survival protein, A1, is dynamically regulated in macrophages after LPS sensing. A1 acts as a key regulator to control the timing of apoptosis in macrophages upon combined loss of BCL‐XL and MCL‐1. Moreover, for the first time, we reveal that inflammatory monocyte survival depends primarily on A1 and MCL‐1 following LPS exposure and, importantly, that A1 upregulation supresses pro‐inflammatory IL‐1β production in response to *Neisseria gonorrhoeae*‐derived outer membrane vesicles (NOMVs) both *in vitro* and *in vivo*.

## Results

### LPS priming delays BAX/BAK‐mediated apoptosis and IL‐1β release in macrophages

**Figure 1:**
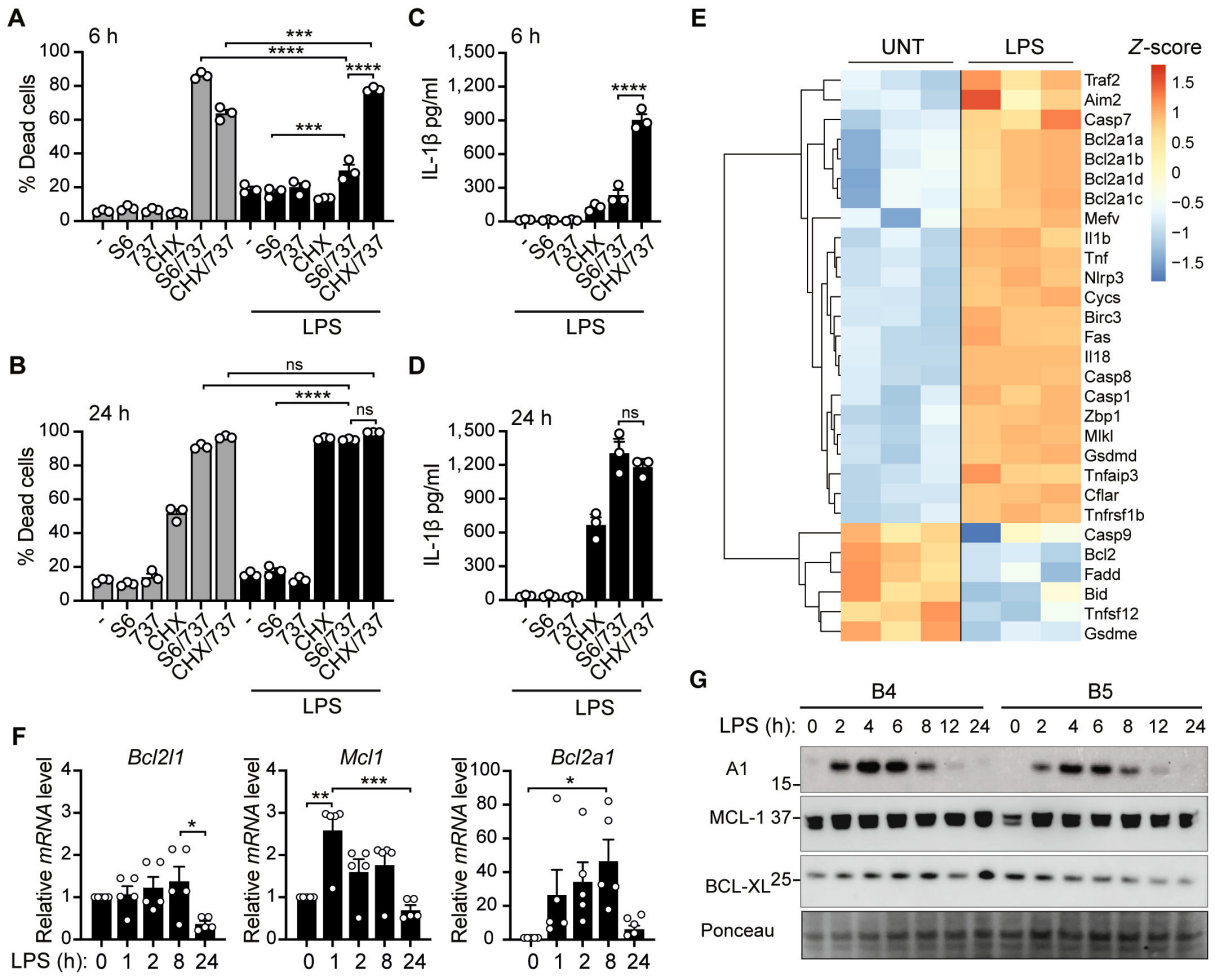
LPS‐primed macrophages exhibit delayed sensitivity to BH3‐mimetics A–DWild‐type (WT) BMDMs were primed, as specified, with B5 LPS (50 ng/ml) for 3 h before addition of BH3‐mimetics S63845 (S6; 10 μM), ABT‐737 (737; 500 nM), and/or cycloheximide (CHX; 20 μg/ml) for 6 or 24 h, as indicated. (A, B) Cell death was measured by flow cytometric analysis of PI uptake. (C, D) IL‐1β levels in the cell supernatants were measured by ELISA.EHeatmap showing significant differentially expressed cell death‐associated genes (derived from apoptosis‐related GO pathways) for untreated (UNT) and LPS‐treated (50 ng/ml) BMDMs at 7 h. Adjusted *P* ≤ 0.05 and cut‐off values logFC ≥ 1 or logFC ≤ −1.FWT BMDMs were treated with LPS (50 ng/ml) for up to 24 h and mRNA transcript levels of *Bcl2l1* (BCL‐XL), *Mcl1* (MCL‐1), and *Bcl2a1* (A1) were examined by qPCR.GWT BMDMs were treated with 50 ng/ml of either B4 or B5 LPS for up to 24 h and cell lysates were interrogated by immunoblot for the indicated proteins. Data information: Each dot represents an individual biological replicate. (A–D, F) Data are representative of at least three independent (A–D), 5 pooled (F) or at least two (G) biological experiments and (A–D, F) presented as the mean + SEM. ns, not significant, *P < 0.05, **P < 0.01, ***P < 0.005, ****P < 0.0001 (one‐way ANOVA with Tukey's multiple comparisons test). (E) Data represent three individual biological replicates per condition. (G) Ponceau stain was used as a control for the loading of total protein. Source data are available online for this figure.

**Figure EV1:**
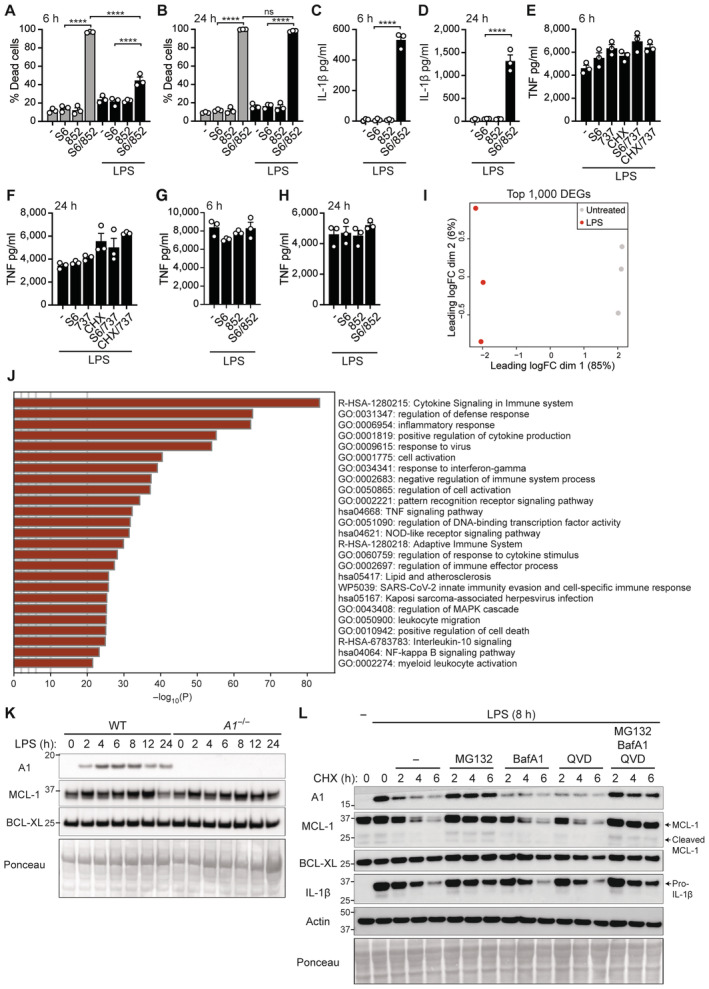
LPS priming delays cell death upon BCL‐XL and MCL‐1 targeting in macrophages A–HWT BMDMs were primed, as specified, with B5 LPS (50 ng/ml) for 3 h before addition of S63845 (S6; 10 μM), ABT‐737 (737; 500 nM), A‐1331852 (852; 1 μM), and/or cycloheximide (CHX; 20 μg/ml) for 6 or 24 h, as indicated. (A, B) Cell death was quantified by flow cytometric analysis of PI uptake. (C, D) IL‐1β and (E–H) TNF levels were measured in the cell supernatants by ELISA.I, JWT BMDMs were treated with B4 LPS for 7 h before RNA isolation and 3′ mRNA sequencing. (I) Multi‐dimensional scaling (MDS) plot of the top 1,000 differentially expressed genes (DEGs) between untreated and LPS‐treated BMDMs. (J) Gene Ontology (GO) analysis showing the top 25 pathways of significant DEGs upregulated in LPS‐treated versus untreated BMDMs. Adjusted *P* ≤ 0.05 and cut‐off values logFC ≥ 1 or logFC ≤ −1.KWT and A1‐deficient (*A1* ^−/−^) BMDMs were primed, as specified, with B5 LPS (50 ng/ml) and cell lysates analysed by immunoblot for up to 24 h for the indicated proteins.LWT BMDMs were primed with B5 LPS (50 ng/ml) for 2–3 h and pre‐treated with MG132 (5 μM), Bafilomycin A1 (BafA1; 100 nM) and/or Q‐VD‐OPh (QVD; 40 μM), as indicated, prior to treatment with and without CHX (20 μg/ml) for the indicated times. Cell lysates were interrogated for the indicated proteins by immunoblot. Data information: Each dot represents an individual biological replicate. (A–H, K, L) Data are representative of at least two independent biological experiments and presented as the mean + SEM. ns, not significant, ****P < 0.0001 (one‐way ANOVA with Tukey's multiple comparisons test). (K, L) Ponceau stains were used as a control for the loading of total protein and *β*‐actin used as an additional loading control. Source data are available online for this figure.

The pro‐survival BCL‐2 family members MCL‐1 and BCL‐XL are reportedly master regulators of intrinsic apoptosis in bone marrow‐derived macrophages (BMDMs) (Speir *et al*, 2016; Vince *et al*, 2018). Here, we confirm that wild‐type (WT) BMDMs undergo rapid apoptotic cell death upon co‐targeting of BCL‐XL and MCL‐1 using the BH3‐mimetic ABT‐737 (737; targets BCL‐2, BCL‐XL and BCL‐w) or the BCL‐XL‐selective inhibitor A‐1331852 (852) in combination with either the MCL‐1‐specific inhibitor S63845 (S6) (Kotschy *et al*, 2016) or the protein synthesis inhibitor cycloheximide (CHX; to deplete MCL‐1; Goodall *et al*, 2016) (Figs 1A and EV1A). In line with previous findings (Chauhan *et al*, 2018; Vince *et al*, 2018), we also observed that dual targeting of BCL‐XL and MCL‐1 with ABT‐737/S63845, A‐1331852/S63845, or ABT‐737/CHX in LPS‐primed macrophages induced cell death (Figs 1A and EV1A) and IL‐1β activation (Figs 1C and EV1C). Surprisingly, upon closer examination of BAX/BAK‐mediated responses at 6 and 24 h, we observed that cell death induced by ABT‐737 or A‐1331852 in combination with S63845 was delayed at 6 h in LPS‐primed cells compared to unprimed macrophages (Figs 1A and B and EV1A and B). Moreover, we observed that both cell death and IL‐1β activation was delayed in LPS‐primed macrophages in response to ABT‐737/S63845 or A‐1331852/S63845 when compared to ABT‐737/CHX treatment, despite efficient priming, as shown by equivalent TNF release (Figs 1A–D and EV1A–H). These findings suggest that, in this context, CHX prevents the early and transient upregulation of an additional pro‐survival factor by LPS.

To identify the short‐lived factor delaying cell death and inflammatory cytokine production, we performed 3′ mRNA sequencing of LPS‐treated versus untreated BMDMs. As expected, we observed significant global changes in gene signatures in BMDMs exposed to LPS (Fig EV1I). Moreover, gene ontology (GO) analyses largely revealed enrichment of processes involved in cytokine and signalling responses, host defence, and cell death regulation in response to LPS (Fig EV1J). Examination of a targeted list of significantly enriched cell death genes from our RNA‐seq dataset revealed significant upregulation of genes involved in inflammasome/pyroptotic (e.g., *Nlrp3*, *Aim2*, *Casp1*, *Gsdmd*, *Il1b*, *Il18*) and extrinsic cell death (e.g., *Fas*, *Tnfrsf1b*, *Birc3*, *Tnfaip3*, *Mlkl*, *Casp8*, *Cflar*) signalling (Fig 1E). In comparison, we observed few significantly enriched genes related to intrinsic apoptotic signalling (e.g., downregulated *Bcl2* and *Bid* expression) in response to LPS treatment, with upregulation of all 4 isoforms of the pro‐survival BCL‐2 family member, *Bcl2a1* (A1), being the most obvious change at 7 h (Fig 1E). Further examination of pro‐survival BCL‐2 family expression over time in response to LPS revealed relatively stable expression of *Bcl2l1* (encodes BCL‐XL) and *Mcl1* mRNA (Figs 1F and G, and EV1K). Aligning with the RNA‐seq data, *Bcl2a1* was absent in unstimulated BMDMs and rapidly, albeit transiently, induced at the mRNA and protein level in response to *Escherichia coli* O111:B4 (B4) and/or O55:B5 (B5) LPS serotypes (Figs 1F and G, and EV1K). In agreement with past reports that A1 is degraded by the proteasome (Kucharczak *et al*, 2005; Herold *et al*, 2006), we observed that the proteasome inhibitor MG132, but not the lysosome inhibitor Bafilomycin A1 (BafA1) nor the pan‐caspase inhibitor Q‐VD‐OPh (QVD), impeded A1 turnover in CHX pulse‐chase experiments performed in LPS‐primed cells (Fig EV1L). As expected, turnover of short‐lived MCL‐1 and pro‐IL‐1β proteins (Vijayaraj *et al*, 2021) was also prevented by MG132 (Fig EV1L). Therefore, transient induction of A1 upon LPS priming may explain why apoptosis is delayed upon selective BCL‐XL and MCL‐1 targeting compared to ABT‐737/CHX treatment, as CHX would rapidly deplete the pool of A1 protein.

### A1 delays BAX/BAK‐mediated cell death, NLRP3 inflammasome and IL‐1β activation in LPS‐primed macrophages

**Figure 2:**
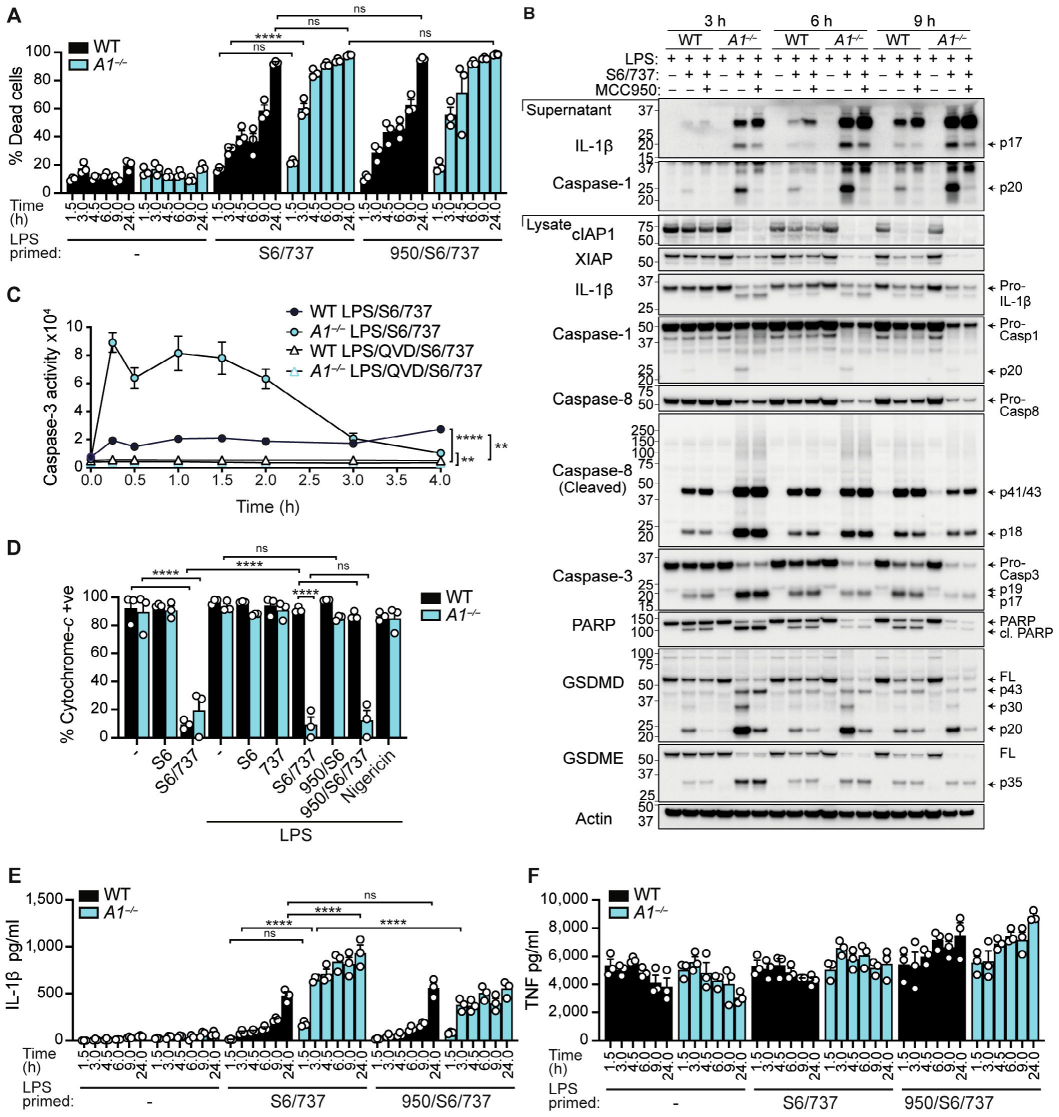
A1 deficiency leads to accelerated apoptotic cell death and inflammasome‐dependent and ‐independent IL‐1β activation in LPS‐primed macrophages A, BWT and A1‐deficient (*A1* ^−/−^) BMDMs were primed with B5 LPS (50 ng/ml) for 3 h, pre‐treated with the NLRP3 inhibitor MCC950 (950; 5 μM) for the last 20–30 min of priming, as specified, prior to the addition of S63845 (S6; 10 μM) and ABT‐737 (737; 500 nM). (A) Cell death was measured over time by PI uptake and flow cytometry. (B) Immunoblot analysis was performed on cell lysates and supernatants for relevant proteins over time.CWT and A1‐deficient (*A1* ^−/−^) BMDMs were primed with B5 LPS (50 ng/ml) for 3 h, pre‐treated with the pan‐apoptotic caspase inhibitor Q‐VD‐OPh (QVD; 40 μM) for the last 30 min of priming, as specified, prior to the addition of S63845 (S6; 10 μM) and ABT‐737 (737; 500 nM). Caspase‐3 activity was measured over time via a DEVDase enzyme assay.DWT and A1‐deficient (*A1* ^−/−^) BMDMs were primed with B5 LPS (50 ng/ml) for 3 h, pre‐treated with the NLRP3 inhibitor MCC950 (950; 5 μM) for the last 20–30 min of priming, as specified, prior to the addition of S63845 (S6; 10 μM) and ABT‐737 (737; 500 nM), as indicated. In some cases, BMDMs were primed with LPS for 3 h and treated with nigericin (10 μM). Cytochrome‐*c* retention, as a measure of MOMP and marker of intrinsic cell death, was assessed by flow cytometric analysis after 3 h of BH3‐mimetic or 1 h of nigericin treatment.E, FWT and A1‐deficient (*A1* ^−/−^) BMDMs were primed with B5 LPS (50 ng/ml) for 3 h, pre‐treated with the NLRP3 inhibitor MCC950 (950; 5 μM) for the last 20–30 min of priming, as specified, prior to the addition of S63845 (S6; 10 μM) and ABT‐737 (737; 500 nM). (E) IL‐1β and (F) TNF levels in the cell supernatants were measured by ELISA. Data information: Each dot represents an individual (A, D–F) or the mean of three (C) biological replicates. Data are representative of at least three (A–C, E, F) or two (D) independent biological experiments and presented as the mean + or ± SEM. ns, not significant, ****P < 0.0001 (A, D–F two‐way ANOVA with Tukey's multiple comparisons test) and (C unpaired two‐tailed Student's *t*‐test on the AUC). (B) *β*‐actin was used as a control for the loading of total protein. Source data are available online for this figure.

**Figure EV2:**
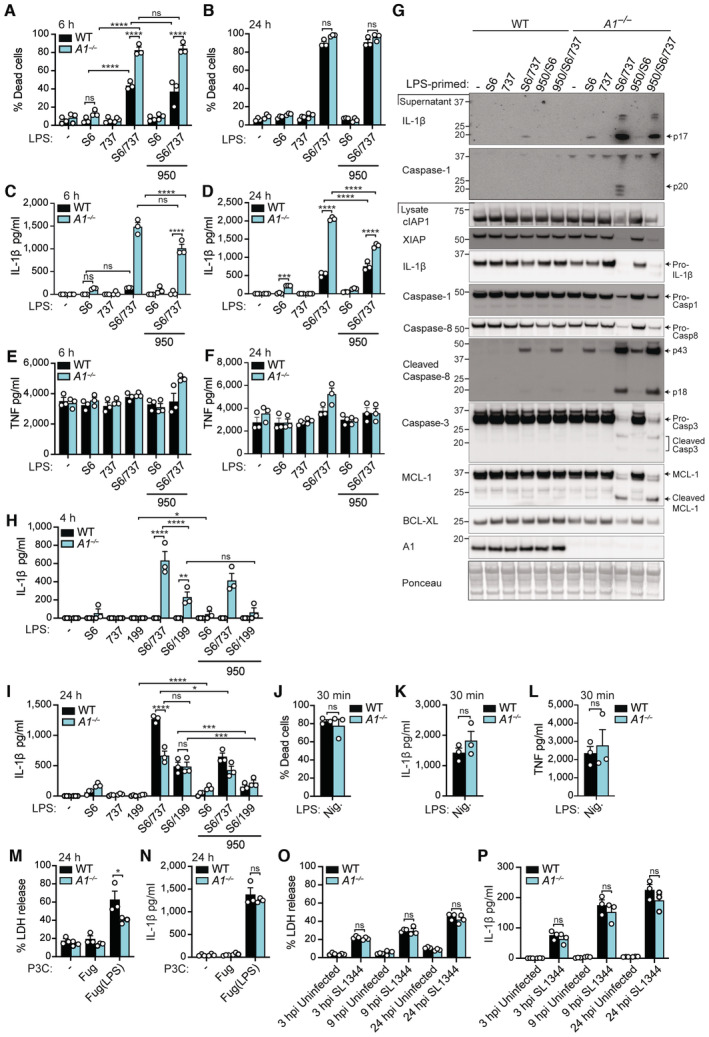
A1 limits intrinsic apoptosis and downstream IL‐1β activation in LPS‐primed macrophages upon BCL‐XL and MCL‐1 targeting A–IWT and A1‐deficient (*A1* ^−/−^) BMDMs were primed with B5 LPS (50 ng/ml) for 3 h, pre‐treated with the NLRP3 inhibitor MCC950 (950; 5 μM) for the final 20–30 min of priming, as indicated, prior to the addition of S63845 (S6; 10 μM), ABT‐737 (737; 500 nM) or ABT‐199 (199; 1 μM), as indicated. (A, B) Cell death was measured by PI uptake and flow cytometric analysis after 6 and 24 h. (C, D, H, I) IL‐1β and (E, F) TNF levels were measured in culture supernatants after 4–6 and 24 h by ELISA. (G) Cell supernatants and lysates were interrogated by immunoblot for the indicated proteins after 6 h.J–LWT and A1‐deficient (*A1* ^−/−^) BMDMs were primed for 3 h with B5 LPS (50 ng/ml) and treated with 10 μM Nigericin for 30 min. (J) Cell death was measured by PI uptake and flow cytometric analysis. (K) IL‐1β and (L) TNF levels were measured in the cell supernatants by ELISA.M, NWT and A1‐deficient (*A1* ^−/−^) BMDMs were primed for 12 h with Pam_3_Cys (500 ng/ml) and then treated with 12.5 μg/ml FuGENE (Fug)‐transfected LPS for 24 h. (M) Cell death was measured using an LDH release assay. (N) IL‐1β levels were measured in the cell supernatants by ELISA.O, PUnprimed WT and A1‐deficient (*A1* ^−/−^) BMDMs were infected with *S*. Typhimurium at MOI 10 for up to 24 h. (O) Cell death was measured using an LDH release assay. (P) IL‐1β levels were measured in the cell supernatants by ELISA. Data information: Each dot represents an individual biological replicate. Data are representative of at least three (A–F, H–L) or two (G, M–P) independent biological experiments and (A–F, H–P) presented as the mean + SEM. ns, not significant, *P < 0.05, ***P < 0.005, ****P < 0.0001 (two‐way ANOVA with Tukey's multiple comparisons test). (G) Ponceau stain was used as a control for the loading of total protein. Source data are available online for this figure.

In order to establish whether A1 limits BAX/BAK‐mediated signalling, we examined cell death responses in LPS‐primed A1‐deficient (*A1* ^−/−^) BMDMs (lacking the functional *Bcl2a1‐a*, *Bcl2a1‐b*, and *Bcl2a1‐d* isoforms) upon BCL‐XL and MCL‐1 targeting (Schenk *et al*, 2017). LPS‐primed A1‐deficient macrophages behaved similarly to WT cells at steady‐state and also remained largely refractory to individual targeting of MCL‐1 (S63845) or BCL‐XL/BCL‐2 (ABT‐737) (Fig EV2A and B). In contrast, co‐targeting of BCL‐XL and MCL‐1 (ABT‐737/S63845) in LPS‐primed A1‐deficient macrophages accelerated cell death (Figs 2A and EV2A and B), which was associated with enhanced apoptotic caspase cleavage and activity, as shown by the rapid and robust detection of caspase‐3‐generated GSDMD (p20 and p43), GSDME (p35), and PARP fragments, and enhanced caspase‐3 activity (that was quenched by the pan‐apoptotic caspase inhibitor Q‐VD‐OPh) (Fig 2B and C). Fittingly, the delayed apoptosis observed in LPS‐primed WT cells coincided with the decline in A1 expression (Figs 1F and G, and EV1K). Importantly, ABT‐737/S63845, but not the canonical NLRP3 inflammasome stimulus nigericin (Fig 2D and Appendix Fig S1), induced the loss of cytochrome‐*c* from LPS‐primed A1‐deficient BMDMs (within ~2–3 h) thereby confirming mitochondrial outer membrane permeabilisation (MOMP) and that cell death was intrinsic in nature.

Correlating with early apoptotic cell death in LPS/ABT‐737/S63845‐treated A1‐deficient BMDMs, we observed elevated bioactive IL‐1β (p17) and inflammasome‐associated caspase‐1 (p20) over time, compared to more delayed secretion in WT cells (Figs 2B and E, and EV2C, D and G). TNF production (marker of inflammasome priming) was not influenced by A1 deficiency nor by BH3‐mimetic treatment (Figs 2F and EV2E and F). Of note, while we did see some inter‐experiment variation in IL‐1β levels detected in WT cell supernatants over time, this likely reflects variation in the magnitude and kinetics of LPS‐induced A1 turnover between experiments, as well as when regulatory events that limit NLRP3 inflammasome activity and pro‐IL‐1β levels are induced (Yabal *et al*, 2019; Vijayaraj *et al*, 2021). Surprisingly, parallel testing of the BCL‐2 inhibitor, ABT‐199, also revealed flexible pro‐survival protein requirements, as ABT‐199/S63845 treatment also induced IL‐1β production in LPS‐primed A1‐deficient BMDMs, albeit at lower levels than with ABT‐737/S63845 (Fig EV2H and I). Importantly, irrespective of whether BCL‐XL or BCL‐2 were targeted alongside MCL‐1, A1 upregulation always delayed early cell death and IL‐1β release to BH3‐mimetic treatment, which would represent the acute phase of an infectious response.

Mechanistically, and mirroring past reports in a WT setting (Vince *et al*, 2018), addition of the NLRP3 inhibitor MCC950 (950) (Coll *et al*, 2015) had no effect on cell death (Figs 2A and EV2A and B) but did block caspase‐1 cleavage and reduce IL‐1β activation in the absence of A1 in LPS/ABT‐737/S63845‐treated BMDMs (Figs 2B and E, and EV2C, D and G), indicating that IL‐1β activation is partly inflammasome mediated. In contrast, A1‐deficient BMDMs responded normally to canonical NLRP3 and noncanonical Caspase‐11 inflammasome stimuli, as well as infection with the intracellular pathogen *Salmonella enterica* Typhimurium (*S*. Typhimurium) (Fig EV2J–P), indicating that A1 only regulates NLRP3 inflammasome activation downstream of BAX/BAK‐dependent apoptosis.

In line with the published BAX/BAK‐signalling model in WT macrophages (Vince *et al*, 2018), enhanced IL‐1β activation in A1‐deficient BMDMs was associated with the rapid loss of extrinsic cellular inhibitor of apoptosis 1 (cIAP1) and X‐linked IAP (XIAP) proteins, and cleavage of caspase‐8 and caspase‐3, which was not abrogated by MCC950 (Figs 2B and EV2G). Altogether, these results suggest that A1 upregulation delays BAX/BAK‐mediated apoptosis and downstream inflammasome‐associated‐caspase‐1‐ and caspase‐8‐mediated IL‐1β activation in macrophages.

### In the absence of apoptotic caspase activity, specific activation of BAX/BAK induces TNF‐ and RIPK3 kinase‐dependent necroptotic signalling and NLRP3 inflammasome activation

**Figure 3:**
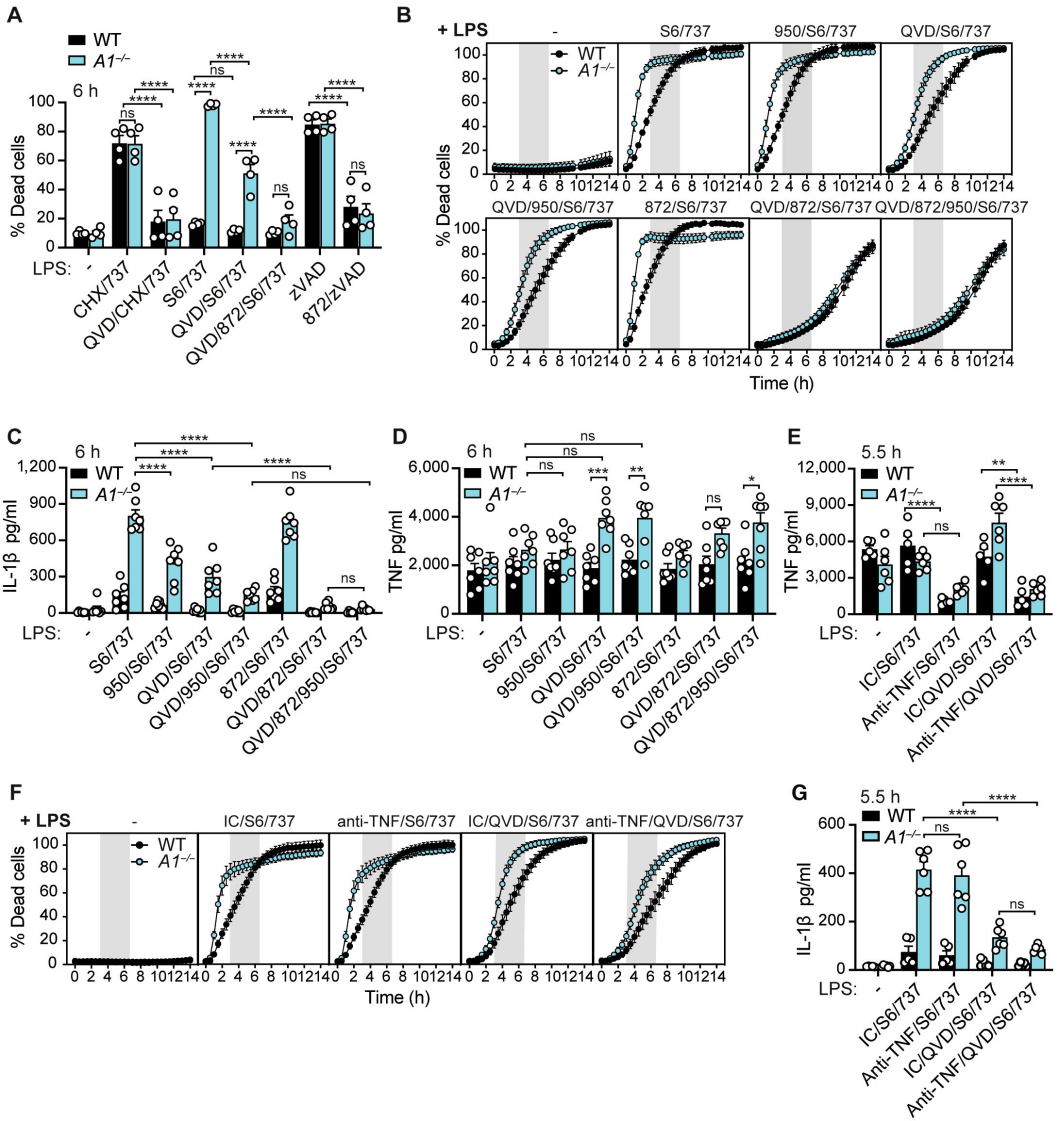
A1 delays BAX/BAK‐mediated necroptosis and NLRP3 inflammasome activation in LPS‐primed macrophages AWT and A1‐deficient (*A1* ^−/−^) BMDMs were primed with LPS for 3 h, then treated with Q‐VD‐OPh (QVD; 40 μM), and GSK'872 (872; 1 μM), as specified, in the last 30 min of priming. Cells were then treated with z‐VAD‐fmk (zVAD 50 μM), cycloheximide (CHX 20 μg/ml), ABT‐737 (737; 500 nM), and S63845 (S6; 10 μM), as indicated. Cell death was measured after 6 h by PI uptake and flow cytometric analysis.B–DWT and A1‐deficient (*A1* ^−/−^) BMDMs were primed with LPS for 3 h, then treated with Q‐VD‐OPh (QVD; 40 μM), MCC950 (950; 5 μM) and GSK'872 (872; 1 μM), as specified, in the last 30 min of priming. (B) Cell death was measured by PI uptake via IncuCyte time‐lapse imaging. (C) IL‐1β and (D) TNF levels were measured in the cell supernatants by ELISA.E–GWT and A1‐deficient (*A1* ^−/−^) BMDMs were treated with 20 μg/ml isotype control (IC; GL113) or anti‐TNF monoclonal antibody (XT22), as indicated. Cells were next treated with LPS (50 ng/ml) for 3 h, incubated with Q‐VD‐OPh (QVD; 40 μM) for the last 30 min of priming, as specified, and treated with S63845 (S6; 10 μM) and ABT‐737 (737; 500 nM). (E) TNF and (G) IL‐1β levels were measured in the cell supernatants by ELISA. (F) Cell death was measured by PI uptake via IncuCyte time‐lapse imaging. Data information: Each dot represents an individual (A, C–E, G) or the mean of at least five (B, F) biological replicates. Data are representative of two pooled biological experiments and presented as the mean + or ± SEM. ns, not significant, *P < 0.05, **P < 0.01, ***P < 0.005, ****P < 0.0001 (two‐way ANOVA with Tukey's multiple comparisons test). Source data are available online for this figure.

**Figure EV3:**
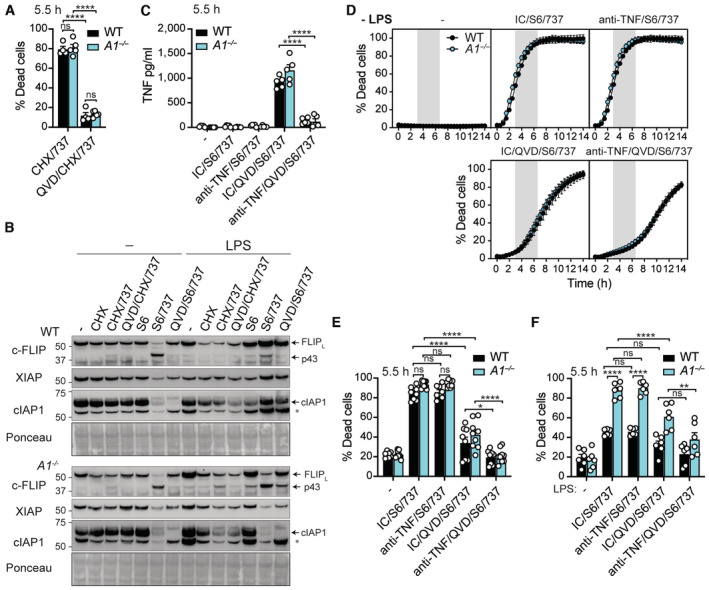
In the presence of caspase inhibition, specific activation of BAX/BAK using S63845 and ABT‐737 triggers TNF‐dependent necroptosis A, BWT and A1‐deficient (*A1* ^−/−^) BMDMs were primed, as indicated, with B5 LPS (50 ng/ml) for 3 h, pre‐treated with the pan‐caspase inhibitor Q‐VD‐OPh (QVD; 40 μM) and/or GSK'872 (872; 1 μM) for the final 20–30 min of priming, as indicated, prior to the addition of S63845 (S6; 10 μM), ABT‐737 (737; 500 nM), CHX (20 μg/ml), and z‐VAD‐fmk (zVAD; 50 μM), as specified, for up to 6 h. (A) Cell death was measured via flow cytometric analysis of PI uptake. (B) Cell lysates were analysed by immunoblot for the indicated proteins after 2 h. *cFLIP re‐probe.C–EWT and A1‐deficient (*A1* ^−/−^) BMDMs were treated with 20 μg/ml isotype control (IC; GL113) or anti‐TNF monoclonal antibody (XT22), as indicated. Cells were next incubated with Q‐VD‐OPh (QVD; 40 μM), as specified, and treated with S63845 (S6; 10 μM) and ABT‐737 (737; 500 nM). (C) TNF levels were measured in the culture supernatants by ELISA. (D) Cell death was measured by PI uptake via time‐lapse IncuCyte imaging. (E) Cell death was measured by PI uptake and flow cytometric analysis after 5.5 h.FWT and A1‐deficient (A1^−/−^) BMDMs were treated with 20 μg/ml isotype control (IC; GL113) or anti‐TNF monoclonal antibody (XT22), as indicated. Cells were next treated with LPS (50 ng/ml) for 3 h, incubated with Q‐VD‐OPh (QVD; 40 μM) for the last 30 min of priming, as specified, and treated with S63845 (S6; 10 μM) and ABT‐737 (737; 500 nM) for 5.5 h. Cell death was measured by PI uptake and flow cytometric analysis. Data information: Each dot represents an individual biological replicate. Data are presented as the mean + or ± SEM from 2 (A, C, D, F) or 3 pooled (E) biological experiments. ns, not significant, *P < 0.05, **P < 0.01, ****P < 0.0001 (two‐way ANOVA with Tukey's multiple comparisons test). (B) Data are representative of two independent biological experiments, and Ponceau stain was used as a control for the loading of total protein. Source data are available online for this figure.

To confirm that BAX/BAK activation in LPS‐primed BMDMs solely triggers apoptosis in the absence of A1, we next examined whether the pan‐apoptotic caspase inhibitor Q‐VD‐OPh could block cell death and associated inflammation. Surprisingly, while ABT‐737/S63845‐treated LPS‐primed A1‐deficient BMDMs clearly displayed reduced cytochrome‐*c* retention within the mitochondria (Fig 2D and Appendix Fig S1), Q‐VD‐OPh pre‐treatment failed to completely block cell death (Fig 3A and B). This is in direct contrast to the complete block in cell death observed in unprimed and LPS‐primed ABT‐737/CHX‐treated WT and *A1* ^−/−^ BMDMs at 6 h (Figs 3A and EV3A) (Vince *et al*, 2018). Since BAX/BAK activity causes IAP loss in macrophages that can promote death‐inducing ripoptosome complex formation (Lawlor *et al*, 2015, 2017), we queried whether, upon caspase‐8 inhibition, RIPK3/MLKL‐dependent necroptosis was being triggered in a proportion of LPS/ABT‐737/S63845‐treated A1‐deficient cells. Strikingly, pre‐treatment with the RIPK3 inhibitor GSK'872 (872) not only blocked necroptosis induced by the classical trigger LPS/z‐VAD‐fmk but also appreciably blocked cell death in LPS/Q‐VD‐OPh/ABT‐737/S63845‐treated A1‐deficient BMDMs at 6 h (Fig 3A and B). Remarkably, matching reports for extrinsic necroptotic RIPK3/MLKL signalling (Conos *et al*, 2017), we found that BAX/BAK‐driven RIPK3 kinase‐dependent necroptotic activity could also engage the NLRP3 inflammasome and activate IL‐1β (i.e., IL‐1β secretion in Q‐VD‐OPh/ABT‐737/S63845 treated cells was dampened by GSK'872 or MCC950) (Fig 3C and D).

In an attempt to clarify how necroptosis occurs following ABT‐737/S63845 but not ABT‐737/CHX treatment upon caspase inhibition, we next interrogated levels of extrinsic cell death regulators, including XIAP, cIAP1, and the caspase‐8 paralogue and inhibitor cellular FLICE inhibitory protein (c‐FLIP, encoded by *Cflar*), whose expression dictates cellular fate (Tummers & Green, 2017). For example, pro‐caspase‐8/c‐FLIP heterodimers can be pro‐apoptotic and limit necroptosis via cleavage of RIPK1, RIPK3, and CYLD (Feng *et al*, 2007; Oberst *et al*, 2011; O'Donnell *et al*, 2011; Legarda *et al*, 2016; Newton *et al*, 2019a; Lalaoui *et al*, 2020). Comparisons between unprimed and LPS‐primed cells lacking A1 expression revealed rapid caspase‐mediated cleavage of XIAP and c‐FLIP (FLIP_L_), as well as caspase‐independent cIAP1 loss (Vince *et al*, 2018), upon ABT‐737/S63845 treatment compared with ABT‐737/CHX (Fig EV3B). Pre‐treatment with Q‐VD‐OPh greatly inhibited XIAP and c‐FLIP_L_ cleavage, although processing was still more evident in LPS/ABT‐737/S63845‐treated A1‐deficient cells, compared to ABT‐737/CHX‐treated controls (Fig EV3B), suggesting that continued protein synthesis promotes necroptosis.

In non‐haematopoietic cells, under caspase‐deficient conditions, BAX/BAK‐driven MOMP has been shown to trigger IAP loss and noncanonical NF‐κB‐associated TNF production (linked to cIAP loss) to induce necroptosis (Giampazolias *et al*, 2017). We therefore queried whether Q‐VD‐OPh/ABT‐737/S63845 treatment in unprimed and LPS‐primed WT and A1‐deficient macrophages promoted TNF‐driven necroptosis. In unprimed macrophages, we observed considerable TNF secretion specifically in response to Q‐VD‐OPh/S63845/ABT‐737 (Fig EV3C). Correspondingly, we observed gradual cell death that was reduced by TNF neutralisation in both WT and A1‐deficient BMDMs (Fig EV3D and E). In contrast, we observed no exaggeration of TNF secretion in LPS‐primed WT cells upon caspase inhibition and BAX/BAK activation, however, we did observe enhanced TNF secretion in LPS‐primed A1‐deficient cells (Fig 3D and E) that display early MOMP (i.e., cytochrome loss). Importantly, we found that, again, TNF neutralisation attenuated necroptotic (Q‐VD‐OPh/S63845/ABT‐737) but not apoptotic (S63845/ABT‐737) cell death, as well as IL‐1β activation in LPS‐primed A1‐deficient BMDMs (Figs 3F and G, and EV3F), albeit to a lesser extent than the RIPK3 inhibitor (Fig 3A, B and C). These data suggest that specific activation of BAX/BAK‐dependent cell death can support TNF‐driven necroptosis and IL‐1β activation in LPS‐primed macrophages when caspase activity is not fully attenuated.

### A1 and MCL‐1 prevent apoptosis and inflammatory signalling in LPS‐primed monocytes

We often observed that loss of MCL‐1 (S63845) and A1 activity could induce modest cleavage of caspase‐8 (p43) and IL‐1β secretion after LPS priming (Fig EV2C, D and G). We therefore considered the possibility that our BMDMs (derived using L929 cell‐conditioned media (LCCM) that contains M‐CSF) might be heterogeneous, akin to other bone marrow‐derived myeloid cell models (Erlich *et al*, 2019), and contain a small percentage of immature monocyte/macrophages sensitive to A1 and MCL‐1 loss. Consequently, we examined responses in day 4 LCCM cultures that predominantly contain CD11b^+^Ly6C^+^F4/80^lo‐int^ inflammatory monocytes (henceforth referred to as BMMo) (Francke *et al*, 2011) (Appendix Fig S2A–C).

**Figure 4:**
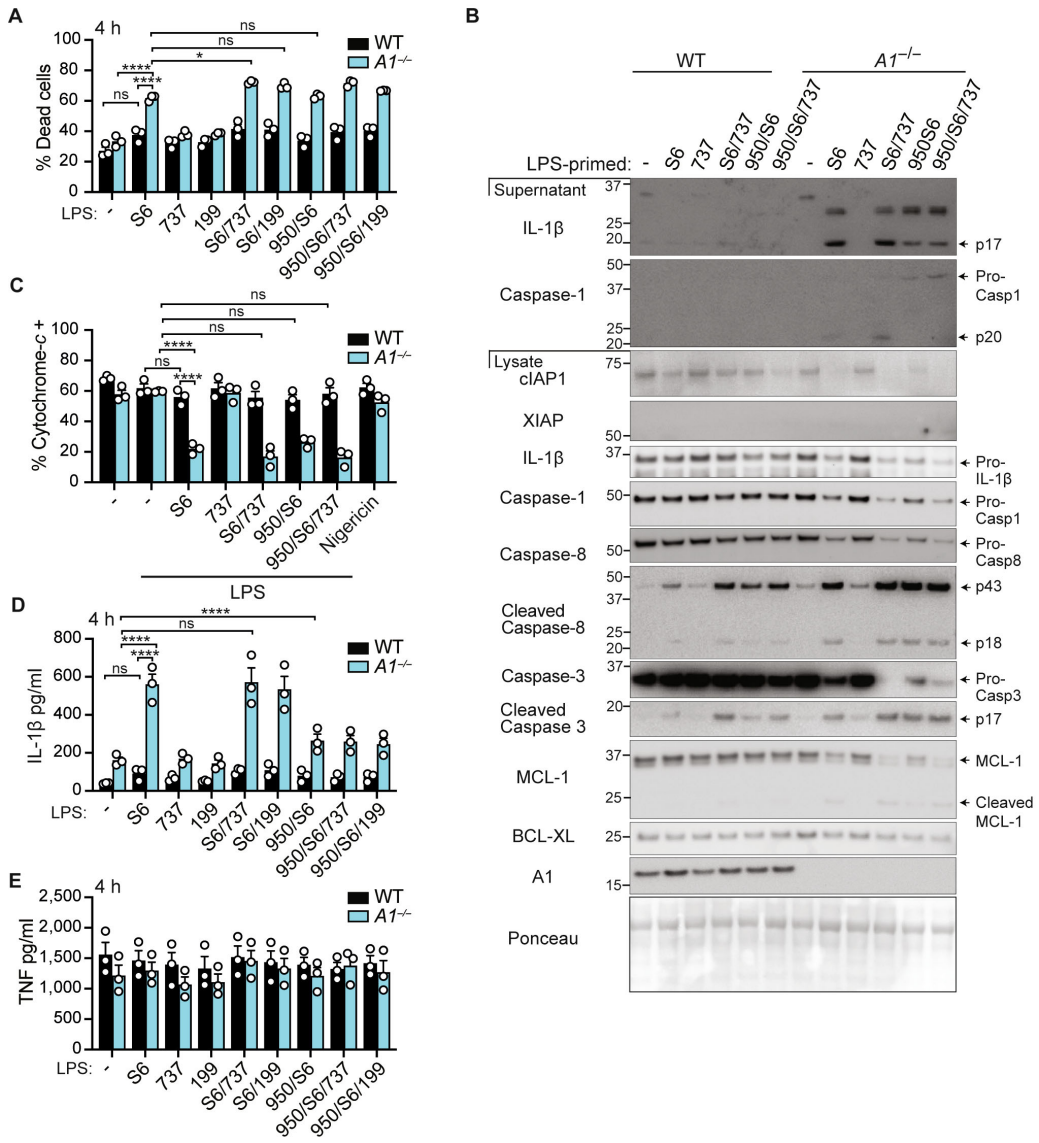
A1 expression limits intrinsic apoptotic signalling in LPS‐primed BMMo upon MCL‐1 targeting alone A, BWT and A1‐deficient (*A1* ^−/−^) bone marrow‐derived monocytes (BMMo) were primed with B5 LPS (50 ng/ml) for 3 h, pre‐treated with the NLRP3 inhibitor MCC950 (950; 5 μM) for the last 20–30 min of priming, as specified, prior to the addition of S63845 (S6; 10 μM), ABT‐737 (737; 500 nM), or ABT‐199 (199; 1 μM), as indicated, for a further 4 h. (A) Cell death was measured by flow cytometric analysis of PI uptake. (B) Immunoblots were performed on cell lysates and supernatants for specified proteins.CWT and A1‐deficient (*A1* ^−/−^) bone marrow‐derived monocytes (BMMo) were primed with B5 LPS (50 ng/ml) for 3 h, pre‐treated with the NLRP3 inhibitor MCC950 (950; 5 μM) for the last 20–30 min of priming, as specified, prior to the addition of S63845 (S6; 10 μM) and ABT‐737 (737; 500 nM) for a further 2 h, as indicated. Alternatively, LPS‐primed BMMo were treated with Nigericin (10 μM) for 1 h. MOMP was evaluated by flow cytometric analysis of cytochrome‐*c* retention.D, EWT and A1‐deficient (*A1* ^−/−^) bone marrow‐derived monocytes (BMMo) were primed with B5 LPS (50 ng/ml) for 3 h, pre‐treated with the NLRP3 inhibitor MCC950 (950; 5 μM) for the last 20–30 min of priming, as specified, prior to the addition of S63845 (S6; 10 μM), ABT‐737 (737; 500 nM), or ABT‐199 (199; 1 μM), as indicated for a further 4 h. (D) IL‐1β and (E) TNF levels were measured in the cell supernatants by ELISA. Data information: Each dot represents an individual biological replicate. Data are representative of at least 3 independent biological experiments and presented as the mean + SEM. ns, not significant, *P < 0.05, ****P < 0.0001 (two‐way ANOVA with Tukey's multiple comparisons test). (B) Ponceau stain was used as a control for the loading of total protein. Source data are available online for this figure.

**Figure EV4:**
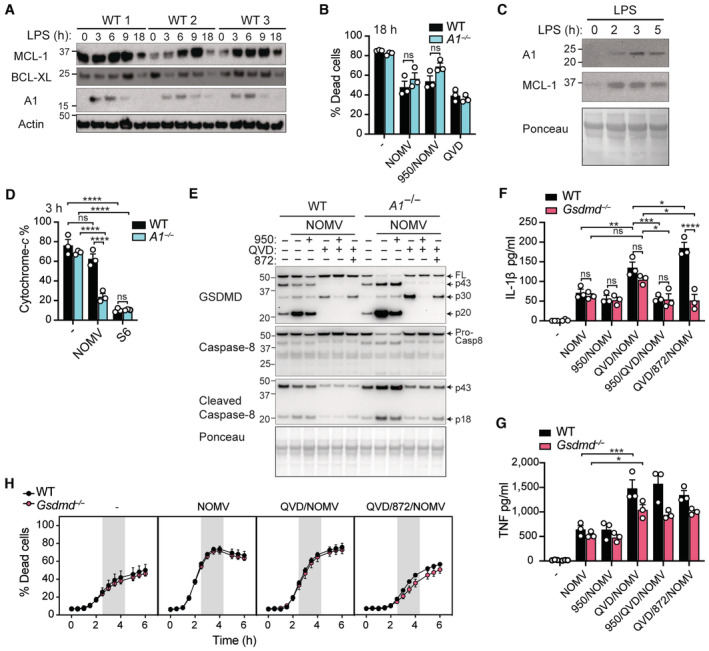
A1 deficiency sensitises inflammatory monocytes to NOMV‐induced BAX/BAK inflammatory signalling AWT BMMo were treated with B5 LPS (50 ng/ml) for up to 18 h before immunoblot analysis of cell lysates for the indicated proteins.BWT and A1‐deficient (*A1* ^−/−^) BMMo were pre‐treated with the NLRP3 inhibitor MCC950 (950; 5 μM) or Q‐VD‐OPh (QVD; 40 μM) for 20–30 min, as indicated, prior to the addition of NOMVs (50 μg/ml), as specified, for a further 18 h. Cell death was measured by flow cytometric analysis of PI uptake.CSorted WT Ly6C^hi^ monocytes were treated with B5 LPS (50 ng/ml) for up to 5 h before immunoblot analysis of cell lysates for the indicated proteins.DWT and A1‐deficient (*A1* ^−/−^) Ly6C^hi^ monocytes were stimulated with NOMVs (50 μg/ml) or S63845 (S6; 10 μM) for 3 h. MOMP‐induced loss of cytochrome‐*c* staining was analysed by flow cytometric analysis.EWT and A1‐deficient (*A1* ^−/−^) Ly6C^hi^ monocytes were pre‐treated with the NLRP3 inhibitor MCC950 (950; 5 μM), pan‐caspase inhibitor Q‐VD‐OPh (QVD; 40 μM) and/or GSK'872 (872; 1 μM), as indicated, for 20–30 min prior to the addition of NOMVs (50 μg/ml) for a further 6 h. Cell supernatants and lysates were interrogated by immunoblot for relevant proteins.F–HWT and Gasdermin D‐deficient (*Gsdmd* ^−/−^) Ly6C^hi^ monocytes were pre‐treated with the NLRP3 inhibitor MCC950 (950; 5 μM), pan‐apoptotic caspase inhibitor Q‐VD‐OPh (QVD; 40 μM) and/or GSK'872 (872; 1 μM), as indicated, for 20–30 min prior to the addition of NOMVs (50 μg/ml) for a further 6 h. (F) IL‐1β and (G) TNF levels were measured in culture supernatants by ELISA. (H) Cell death was measured by PI uptake via time‐lapse IncuCyte imaging. Data information: Each dot represents an individual (B, D, F, G) or the mean of three (H) biological replicates. Data are representative of at least two independent (B, D) or one (F–H) biological experiment and are presented as the mean + SEM or mean ± SEM. ns, not significant, *P < 0.05, **P < 0.01, ***P < 0.005, ****P < 0.0001 (two‐way ANOVA with Tukey's multiple comparisons test). Blots represent three individual biological replicates (A) or at least three (C) or two (E) biological experiments; Ponceau stain was used as a loading control. Source data are available online for this figure.

Reminiscent of BMDMs (Figs 1G and EV1K), WT BMMo exhibited rapid and transient upregulation of A1 in response to LPS with levels diminishing between 9 and 18 h, while BCL‐XL levels remained relatively unchanged (Fig EV4A). Uniquely, BMMo also demonstrated transient MCL‐1 upregulation that also peaked at 9 h (Fig EV4A). Consistent with our theory that A1 and MCL‐1 repress BAX/BAK activation in monocytes, LPS‐primed A1‐deficient BMMo, but not WT cells, underwent rapid apoptosis (~4 h) upon MCL‐1 inhibition (S63845), and additional targeting of BCL‐XL (ABT‐737) or BCL‐2 (ABT‐199) did not further enhance cell death signalling (Fig 4A–C). Evidence of loss of cytochrome‐*c* retention upon MCL‐1 targeting confirmed that LPS‐primed A1‐deficient BMMo were undergoing MOMP (i.e., intrinsic apoptosis) (Fig 4C and Appendix Fig S2D). In addition to triggering maximal BAX/BAK‐mediated cell death, targeting MCL‐1 alone in LPS‐primed A1‐deficient BMMo triggered high levels of bioactive IL‐1β (Fig 4B and D), despite equivalent priming (Fig 4E). Paralleling our BAX/BAK signalling model in macrophages, this IL‐1β activation was associated with robust cIAP1 loss and heightened caspase‐1, caspase‐8, and caspase‐3 cleavage to their active fragments (Fig 4B). XIAP was not detectable in monocytes (Fig 4B), which fits with a previous report that low XIAP levels in CD11b^+^Ly6C^+^F4/80^lo^ monocytes sensitises them to cIAP1/2‐targeting with the Smac‐mimetic, Birinapant (Rijal *et al*, 2018). Importantly, IL‐1β activation was only partially blocked by MCC950, suggesting that both caspase‐1 and caspase‐8 contribute to IL‐1β maturation in monocytic cells. Thus, A1 and MCL‐1 act together to limit intrinsic apoptosis and pro‐inflammatory IL‐1β production in LPS‐primed monocytes.

### A1 deficiency sensitises monocytes to OMVs derived from *Neisseria*

**Figure 5:**
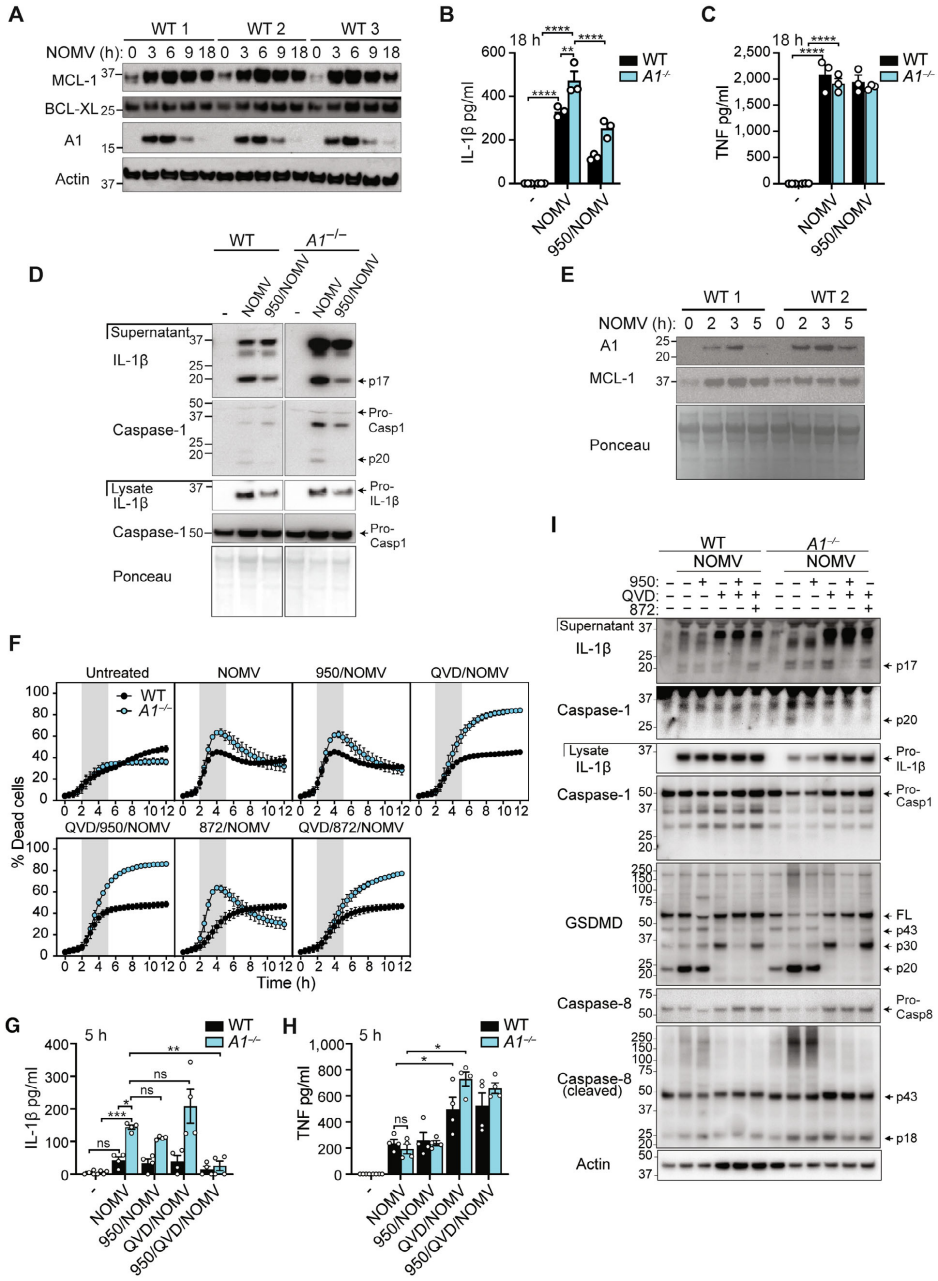
A1 restricts IL‐1β production and cell death in primary Ly6C^hi^ monocytes treated with *Neisseria gonorrhoeae*‐derived OMVs (NOMVs) AWT BMMo were treated with NOMVs for up to 18 h and cell lysates were interrogated for relevant proteins by immunoblot, as indicated.B–DWT and A1‐deficient (*A1* ^−/−^) BMMo were pre‐treated, as indicated, with MCC950 (950) (5 μM) for 20–30 min and cultured with 50 μg/ml of NOMVs for 18 h. (B) IL‐1β and (C) TNF levels were measured in the cell supernatants by ELISA. (D) Cell supernatants and lysates were analysed by immunoblot for specified proteins.EPrimary Ly6C^hi^ sorted monocytes from WT mice were cultured with 50 μg/ml of NOMVs for up to 5 h. Cell lysates were interrogated by immunoblot for specified proteins.F–IPrimary Ly6C^hi^ sorted monocytes from WT and A1‐deficient mice were pre‐treated with MCC950 (950) (5 μM), Q‐VD‐OPh (QVD) (40 μM), or GSK'872 (872) (1 μM) for 15–30 min, as indicated, and cultured with 50 μg/ml of NOMVs for up to 12 h. (F) Cell death was quantified by time‐lapse live cell imaging of PI uptake in cell tracker green (CTG)‐labelled monocytes. (G) IL‐1β and (H) TNF levels in the cell supernatants were measured by ELISA at 5 h. (I) Cell supernatants and lysates were analysed by immunoblot for specified proteins at 5–6 h. Data information: Each dot represents an individual biological replicate (B, C, G, H). Data are representative of at least 3 independent (B, C) or 4 pooled (G, H) biological experiments and presented as the mean + SEM. ns, not significant, *P < 0.05, **P < 0.01, ***P < 0.005, ****P < 0.0001 (two‐way ANOVA with Tukey's multiple comparisons test). (F) Each dot represents the mean of *n* = 2 biological replicates per condition and are representative of 3 independent biological experiments and presented as the mean + SD. (A, D, E, I) Data are representative of at least two biological experiments, Ponceau stain or *β*‐actin was used as a control for the loading of total protein and freshly harvested BMMo lysates were used as the control in (D) to avoid spontaneous cell death in culture. Source data are available online for this figure.

Recently, a number of pathogens have been shown to inhibit host protein synthesis and deplete MCL‐1, rendering cells sensitive to rapid killing upon BCL‐XL loss/inactivation (Speir *et al*, 2016; Suzuki *et al*, 2018; Orzalli *et al*, 2021). In fact, outer membrane vesicles derived from the emerging superbug *Neisseria gonorrhoeae* (NOMVs) have been shown to cause mitochondrial dysfunction, leading to inhibition of host protein translation, and delayed BAX/BAK‐mediated cell death and NLRP3 inflammasome activation in macrophages (Deo *et al*, 2020). Thus, based on the specific requirement for A1 and MCL‐1 in monocyte survival upon LPS exposure, we postulated that LPS contained within NOMVs might transiently upregulate A1 (and MCL‐1) to delay BAX/BAK‐mediated inflammasome activation in the short‐lived inflammatory monocyte population (Yona *et al*, 2013). NOMV treatment induced strong but transient A1 expression in BMMo between 3 and 6 h (Fig 5A). MCL‐1 protein was also markedly upregulated and sustained upon NOMV treatment, although it modestly declined over time, while BCL‐XL was constitutively expressed (Fig 5A). As predicted, A1‐deficient BMMo displayed heightened IL‐1β responses to NOMVs, compared to WT cells (~18 h; Fig 5B and D), which was not associated with altered TNF production (Fig 5C). As seen with S63845 treatment of A1‐deficient monocytes, IL‐1β maturation and release in response to NOMVs was partially blocked by the NLRP3 inhibitor MCC950 (Fig 5B and D). However, unexpectedly, untreated BMMo underwent spontaneous apoptosis upon M‐CSF withdrawal and this confounded examination of NOMV‐induced cell death kinetics (Fig EV4B). Nevertheless, these data indicate that NOMV‐induced A1 expression limits NLRP3 inflammasome and IL‐1β activation in BMMo.

To obtain greater mechanistic insight into cell death responses and to better model the inflammatory infiltrate recruited to the site of *N. gonorrhoeae* infection, we next examined NOMV responses in freshly sorted primary Ly6C^hi^ monocytes (Appendix Fig S3). Strikingly, in response to either LPS or NOMV treatment, WT Ly6C^hi^ monocytes exhibited an even more transient induction of A1 (Figs 5E and EV4C), when compared to BMDMs (Figs 1G and EV1K) or BMMo (Fig EV4A). Correspondingly, A1‐deficient Ly6C^hi^ monocytes were more sensitive than WT cells to NOMV‐induced intrinsic apoptosis (Fig 5F), as highlighted by reduced cytochrome‐*c* retention at 3 h post‐NOMV treatment (Fig EV4D and Appendix Fig S4). Activation of BAX/BAK signalling in A1‐deficient Ly6C^hi^ monocytes also triggered rapid IL‐1β activation (Fig 5G and I) but caused no major perturbation in TNF secretion between genotypes (Fig 5H). Again, cleavage of caspase‐1 and caspase‐8 were both associated with IL‐1β activation, as well as with the cleavage of shared substrate GSDMD at Asp276 to generate the active N‐terminal p30 pore‐forming fragment (Figs 5I and EV4E). However, in line with the exaggerated apoptosis observed in A1‐deficient Ly6C^hi^ monocytes, NOMVs dominantly triggered caspase‐3‐mediated inactivation of GSDMD (to generate p20 and p43 fragments) (Figs 5I and EV4E) (Taabazuing *et al*, 2017; Chen *et al*, 2019). As predicted based on the current BAX/BAK‐mediated inflammatory signalling model (Vince *et al*, 2018), NLRP3 inhibition using MCC950 only partially blocked IL‐1β activation (Fig 5G and I) and did not block cell death in either the WT or A1‐deficient monocytes (Fig 5F). In contrast, apoptotic caspase inhibitor Q‐VD‐OPh delayed cell death by only ~1 to 2 h and, similar to MCC950, did not effectively block IL‐1β activation (Fig 5F, G and I). In fact, Q‐VD‐OPh could actually enhance inflammasome‐associated IL‐1β and GSDMD p30 activity in NOMV‐treated A1‐deficient inflammatory monocytes – an activity that was inhibited by MCC950 (Figs 5F, G and I, and EV4E). Results suggest that Q‐VD‐OPh most likely predisposes NOMV‐treated A1‐deficient Ly6C^hi^ monocytes to either pyroptosis or necroptotic signalling pathways that can also activate the NLRP3 inflammasome (Conos *et al*, 2017). However, pre‐treatment of A1‐deficient cells with Q‐VD‐OPh in combination with MCC950 failed to further delay NOMV‐induced cell death, while the RIPK3 kinase inhibitor GSK'872 (blocks necroptosis) modestly delayed cell death (Fig 5F). Crucially, IL‐1β secretion was almost completely abrogated by combined pre‐treatment with Q‐VD‐OPh and MCC950 (Fig 5G and I), confirming that caspase‐1 and caspase‐8 were both required for full IL‐1β activity. Interestingly, despite the fact GSK'872 was able to delay NOMV‐induced cell death in the absence of apoptotic caspases (Fig 5F), akin to our BH3‐mimetic studies (Fig 3A and B), blocking necroptotic signalling failed to prevent caspase‐1, IL‐1β, and GSDMD (p30) cleavage (Figs 5I and EV4E), suggesting pyroptotic signalling is triggered. Supporting this idea, we found that IL‐1β secretion (and cell death) was comparable in *Gsdmd* ^−/−^ monocytes upon NOMV‐induced apoptotic (NOMV) or necroptotic (QVD/NOMV) signalling but IL‐1β secretion was completely blocked in *Gsdmd* ^−/−^ monocytes when both pathways were inhibited (QVD/872/NOMV) (Fig EV4F–H). Collectively, these results suggest that, while A1 loss in inflammatory monocytes accelerates BAX/BAK‐mediated cell death and inflammatory signalling triggered by NOMVs, in the absence of apoptotic caspases this pathway can default to necroptosis and downstream NLRP3 inflammasome activation. Yet when both apoptosis and necroptosis are limited, NOMV‐derived LPS may be sensed by caspase‐11 to promote NLRP3 activity. It is important to note that, as these cells undergo MOMP (Ekert *et al*, 2004), they will die in a manner independent of apoptotic caspases, RIPK3, and inflammasome activity.

### Enhanced IL‐1β activity in A1‐deficient mice to NOMV injection *in vivo*

**Figure 6:**
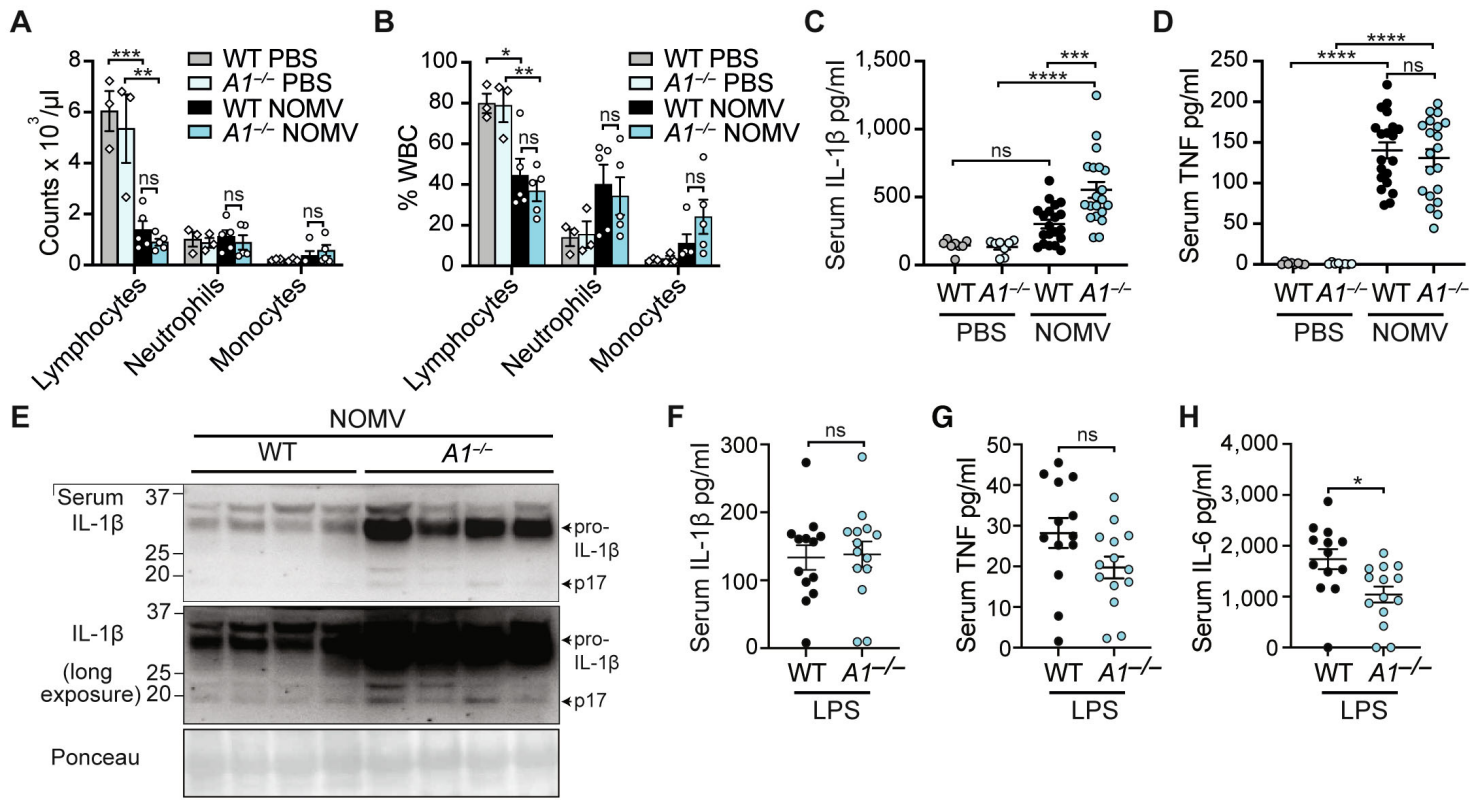
A1‐deficient mice display heightened IL‐1β levels in response to *Neisseria gonorrhoeae*‐derived OMVs A–EWT and A1‐deficient mice were injected intraperitoneally with 100 μg of NOMVs or PBS. At 6 h, mice were sacrificed, and blood was collected for analysis. (A) Peripheral white blood cell (WBC) counts or (B) % WBC were measured using an ADVIA instrument. (C) IL‐1β and (D) TNF levels were measured in the serum by ELISA. (E) Serum IL‐1β activity (p17) was assessed by immunoblot.F–HWT and A1‐deficient mice were injected intraperitoneally with 100 μg of LPS. At 6 h, mice were sacrificed, and blood was collected for analysis. (F) IL‐1β, (G) ΤΝF and (H) IL‐6 levels were measured in the serum by ELISA. Data information: Each dot represents an individual biological replicate. Data are representative of one (A, B) or at least 2 pooled (C, D, F–H) biological experiments and presented as the mean ± SEM. ns, not significant, *P < 0.05, **P < 0.01, ***P < 0.005, ****P < 0.0001 (A–D one‐way ANOVA with Tukey's multiple comparisons test) and (F–H unpaired, two tailed Student's *t*‐test). (E) Immunoblot of *n* = 4 individual biological replicates per genotype, Ponceau stain was used as a control for the loading of total protein. Source data are available online for this figure.

**Figure EV5:**
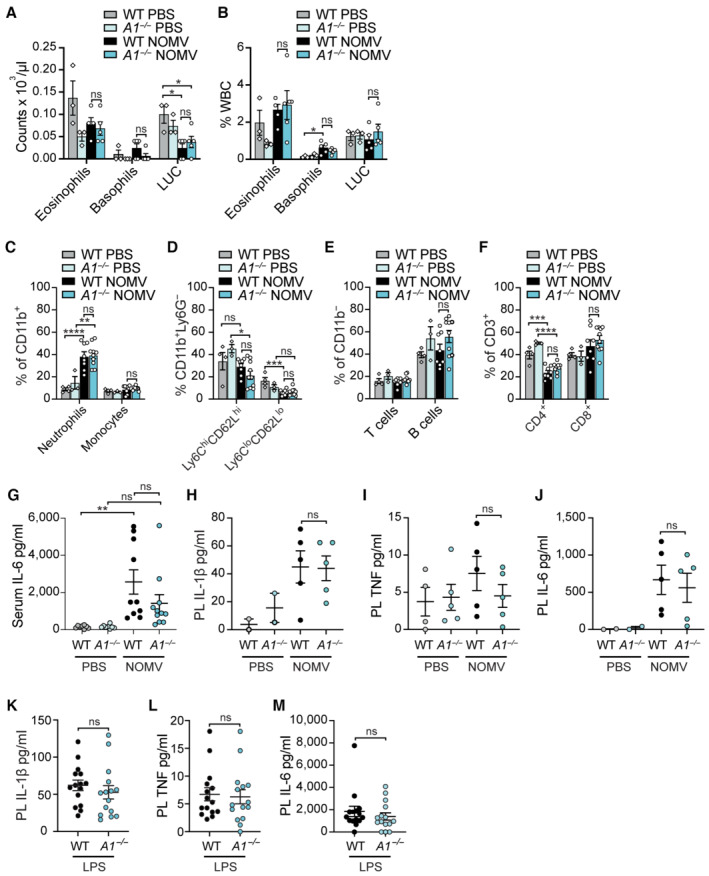
Effect of A1 deficiency on systemic and local inflammatory responses to NOMV or LPS injection A–JWT and A1‐deficient (*A1* ^−/−^) mice were injected intraperitoneally with 100 μg of NOMVs or PBS and peripheral blood and peritoneal lavage fluid harvested after 6 h. Immune cell subsets in the peripheral blood were quantified using (A, B) ADVIA or (C–F) flow cytometry. (A) Leukocyte counts and (B) populations as the % of total white blood cells (WBC). (C–F) Percentage % of peripheral blood leukocytes that are (C) neutrophils (CD11b^+^Ly6G^+^) and monocytes (CD11b^+^Ly6G^−^). (D) % of monocytes (CD11b^+^Ly6G^−^) that are inflammatory (Ly6C^hi^CD62L^hi^) or resident (Ly6C^lo^CD62L^lo^) subsets. (E) % of non‐myeloid (CD11b^−^) cells that are T cells (CD3^+^) and B cells (B220^+^). (F) Proportion % of (CD3^+^) T cells that are CD4^+^ (CD3^+^CD4^+^) or CD8^+^ (CD3^+^CD8^+^) T cells. (G–J) Levels of (G, J) IL‐6, (H) IL‐1β, and (I) TNF were measured in the serum (G) and peritoneal lavage fluid (H–J) by ELISA.K–MWT and A1‐deficient mice (*A1* ^−/−^) were injected intraperitoneally with 100 μg of LPS and peritoneal lavage fluid harvested after 6 h. Levels of (K) IL‐1β, (L) TNF, and (M) IL‐6 were measured in the peritoneal lavage fluid by ELISA. Data information: Each symbol represents an individual biological replicate. Data are expressed as mean ± SEM and are representative of one (A, B, G–J) or two pooled (C–F, K–M) biological experiments. *P < 0.05, **P < 0.01, ***P < 0.005, ****P < 0.0001 (A–J, one‐way ANOVA with Tukey's multiple comparisons test) and (K–M, unpaired, two‐tailed Student's *t*‐test). Source data are available online for this figure.

Having established that A1 restricts NOMV‐induced monocyte cell death and IL‐1β activation *in vitro*, we next examined whether A1 upregulation could dampen the acute inflammatory response to *N. gonorrhoeae* extracellular infection *in vivo*. To do this, we injected WT and A1‐deficient mice intraperitoneally with 100 μg of NOMVs and after 6 h harvested blood for ADVIA differential cell counts and flow cytometric analysis of leukocytes, and serum and peritoneal lavage fluid for cytokine analysis. Analysis of immune cells in the blood revealed that both the WT and *A1* ^−/−^ NOMV‐treated mice exhibited the lymphopenia characteristic of sepsis but showed no meaningful differences in immune cell subsets between genotypes at this time point (Figs 6A and B, and EV5A–F and Appendix Fig S5). Pro‐inflammatory cytokine analysis revealed that serum IL‐1β was significantly increased in NOMV‐challenged *A1* ^−/−^ mice compared to WT (Fig 6C), while serum TNF (Fig 6D) and IL‐6 (Fig EV5G), as well as cytokine levels in the local peritoneal lavage fluid were equivalent (Fig EV5H–J). Crucially, serum immunoblot analysis revealed that, although IL‐1β was mostly released in its immature pro‐form, bioactive IL‐1β (p17) was particularly evident in NOMV‐injected A1‐deficient mice (Fig 6E). In contrast to NOMV‐induced responses, IL‐1β levels in the serum and peritoneal lavage fluid were not perturbed in A1‐deficient mice following low‐dose LPS challenge (Figs 6F and EV5K). Similarly, serum and peritoneal fluid TNF and IL‐6 levels were also largely equivalent between WT mice and A1‐deficient mice, although *A1* ^−/−^ mice did exhibit reduced levels of serum IL‐6 (Figs 6G and H, and EV5L and M). Overall, these data demonstrate that A1 plays a systemic role *in vivo* in limiting inflammation arising from exposure to *N. gonorrhoeae*‐derived OMVs, which is not mediated solely via LPS‐sensing.

## Discussion

It is now becoming clear that apoptotic signalling can crosstalk with inflammatory cell death (e.g., pyroptosis) and promote NLRP3 inflammasome and IL‐1β activation (Yabal *et al*, 2019; Speir & Lawlor, 2021). This work has centred around the activities of extrinsic apoptotic caspase‐8, which, to date, include the regulation of transcriptional responses (Allam *et al*, 2014; Philip *et al*, 2016; Simpson *et al*, 2022), association with ASC inflammasomes (Sagulenko *et al*, 2013; Fritsch *et al*, 2019; Newton *et al*, 2019b), NLRP3 inflammasome activation (Yabal *et al*, 2014; Lawlor *et al*, 2015; Gaidt *et al*, 2016), and direct proteolysis of inflammatory caspase substrates GSDMD, IL‐18, and IL‐1β (Maelfait *et al*, 2008; Bossaller *et al*, 2012; Vince *et al*, 2012; Orning *et al*, 2018; Sarhan *et al*, 2018). However, two recent studies have now shown that BAX/BAK activation in LPS‐primed macrophages promotes NLRP3 inflammasome‐ and caspase‐8‐mediated IL‐1β activation (Chauhan *et al*, 2018; Vince *et al*, 2018). Our data now reveal that the LPS inducible pro‐survival protein A1 is a critical regulator of this pathway, tightly controlling the timing of cell death and IL‐1β release in both macrophages and inflammatory Ly6C^hi^ monocytes. We demonstrate for the first time that, distinct from macrophages, A1 and MCL‐1 are key survival proteins in Ly6C^hi^ monocytes upon LPS sensing. Finally, we show that A1 upregulation is physiologically relevant, as OMVs derived from Gram‐negative *N. gonorrhoeae*, which target the mitochondria and activate BAX/BAK‐dependent signalling, induce exaggerated IL‐1β activation in A1‐deficient monocytes *in vitro* and in A1‐deficient mice *in vivo*.

Loss of MCL‐1 and BCL‐XL (or BCL‐2) activity in LPS‐primed macrophages can trigger BAX/BAK‐dependent inflammation associated with the loss of ripoptosome repressors XIAP and cIAP1, and through effector caspase‐3/−7‐mediated activation of caspase‐8 to directly cleave IL‐1β, and activation of NLRP3 via pannexin‐1 channel‐dependent K^+^ efflux (Chauhan *et al*, 2018; Vince *et al*, 2018; Chen *et al*, 2019). One puzzling element to this proposed model has been the fact that LPS priming significantly delays BAX/BAK‐mediated cell death signalling induced by selective targeting of MCL‐1 and BCL‐XL (Vince *et al*, 2018). We now reveal the explanation for this discrepancy is the transient expression of the pro‐survival protein A1, which not only limits cell death upon MCL‐1 and BCL‐XL targeting but also, unexpectedly, prevents the more protracted BAX/BAK inflammatory signalling observed upon MCL‐1 and BCL‐2 inhibition. Considering the emerging role for BCL‐2 in regulating macrophage survival under pathogen‐induced stress (Chauhan *et al*, 2018; Simpson *et al*, 2022), understanding how short‐lived A1 promotes late‐stage survival dependency on BCL‐2 will be of interest.

Our attempts to verify that specific loss of MCL‐1, BCL‐XL, and A1 activity triggers apoptosis in macrophages uncovered that these cells are poised to die via necroptosis basally and upon TLR4 ligation. This contrasts with the block in cell death achieved with pan‐apoptotic‐caspase inhibition in LPS‐primed BMDMs treated with ABT‐737/CHX (Vince *et al*, 2018). In the case of ABT‐737/CHX treatment, depletion of the LPS‐inducible c‐FLIP by CHX is proposed to promote a pro‐apoptotic caspase‐8 homodimeric complex that is more effectively inhibited by Q‐VD‐OPh (Maelfait *et al*, 2008; Wang *et al*, 2008; Allam *et al*, 2014; Brumatti *et al*, 2016). Why necroptosis occurs in a proportion of LPS/ABT‐737/S68345‐treated A1‐deficient BMDMs upon caspase inhibition is less clear and warrants future investigation. It is tempting to speculate that, due to ineffective caspase‐8 inhibition in these cells (Brumatti *et al*, 2016; Simpson *et al*, 2022), a threshold of c‐FLIP expression and/or reduced IAP levels predisposes them to necroptosis through TNF signalling. Overall, this work shows that, while A1, MCL‐1, and BCL‐XL act chiefly to limit intrinsic apoptosis in LPS‐primed macrophages, these cells are capable of engaging other cell death modalities, akin to the flexibility discovered during extrinsic cell death signalling in innate and non‐immune cells (Newton *et al*, 2014, 2019b; Fritsch *et al*, 2019; Doerflinger *et al*, 2020; Malireddi *et al*, 2020; Simpson *et al*, 2022).

We found that A1 was strongly but transiently induced by LPS in bone marrow monocytes, akin to macrophages. However, BH3‐mimetic screening revealed a distinct pro‐survival protein profile, with MCL‐1 and A1 playing a vital role in limiting intrinsic apoptosis in inflammatory monocytes. Remarkably, loss of MCL‐1 and A1 activity also triggered IL‐1β activation, via caspase‐8 and NLRP3 inflammasome activity, confirming that A1 delays BAX/BAK‐mediated inflammatory signalling in both monocytes and macrophages. Both A1 and MCL‐1 have previously been implicated in limiting neutrophil cell death basally, upon activation and to death receptor FAS ligation (Dzhagalov *et al*, 2007; Vier *et al*, 2016; Csepregi *et al*, 2018; Schenk *et al*, 2020). However, despite these observations, and to our knowledge, this is the first report that A1 and MCL‐1 regulate Ly6C^hi^ monocyte turnover. It is worth acknowledging that, due to the embryonic lethality of MCL‐1 knockout mice (Rinkenberger *et al*, 2000), most studies have examined monocyte homeostasis using mice with conditional deletion of *Mcl‐1* in myeloid cells (LysM Cre transgenic) that renders them neutropenic (Steimer *et al*, 2009; Csepregi *et al*, 2018). A potential caveat to these studies is the possibility that residual MCL‐1 expression maintains monocyte numbers due to ineffective deletion, as seen previously with extrinsic regulators (Kang *et al*, 2013; Wong *et al*, 2014; Lawlor *et al*, 2015; Vince *et al*, 2018), and the potential for compensatory myelopoiesis or pro‐survival protein expression (Dzhagalov *et al*, 2007). Overall, the critical need to maintain MCL‐1 sufficiency is underscored by the relatively normal granulopoiesis observed in heterozygous MCL‐1 mutant mice (Brinkmann *et al*, 2017), and the relative resistance of murine immune cells to S63845 (Vince *et al*, 2018; Rohner *et al*, 2020).

*Neisseria gonorrhoeae*‐derived outer membrane vesicles deliver LPS and a range of effector proteins and toxins to host cells (Pathirana & Kaparakis‐Liaskos, 2016; Dhital *et al*, 2021), including the mitochondria‐targeting PorB protein that disrupts mitochondrial homeostasis and induces MCL‐1 depletion to trigger late‐stage apoptosis in macrophages that we have shown can be amplified by BCL‐XL targeting (Deo *et al*, 2018, 2020). Our data support a role for A1 (and MCL‐1) in limiting NOMV‐induced BAX/BAK‐mediated apoptosis, necroptosis, and possibly pyroptosis in infiltrating inflammatory monocytes that act as bystander cells during extracellular infections. A1 expression also limited NOMV‐induced IL‐1β responses that were partly dependent on caspase‐8 and NLRP3 inflammasome activity. Intriguingly, these results differ from a recent finding in macrophages that NOMV‐induced BAK activation solely triggers K^+^ efflux‐dependent NLRP3 inflammasome responses (Deo *et al*, 2020). Consequently, our results highlight the variance in cell death signalling molecules engaged upon NOMV exposure in individual innate immune cells. This divergence is likely to play a vital role in infectious outcomes, where the rapid turnover and cell death plasticity in infiltrating inflammatory Ly6C^hi^ monocytes will be key to driving acute inflammation, while delayed cell death in longer‐lived macrophages may preserve the replicative niche. Impressively, A1 expression reduced sepsis‐induced IL‐1β activation in mice exposed to NOMVs despite us failing to observe any depletion of innate immune cells and/or signs of local inflammation. Therefore, it remains to be seen whether over time A1 expression impacts select populations. Moreover, it is unclear whether A1 upregulation is beneficial or deleterious for the host during infection, since this may be multi‐factorial depending on the pathogen (Gangoda *et al*, 2021), cell types involved, and the role IL‐1β plays in shaping the immune response. Nevertheless, with a growing number of pathogens reported to deplete MCL‐1 and target BCL‐XL or BCL‐2, and reports defining the potential use of BH3‐mimetic drugs as antimicrobials (Ohmer *et al*, 2016; Speir *et al*, 2016; Chauhan *et al*, 2018; Suzuki *et al*, 2018; Deo *et al*, 2020; Inde *et al*, 2021; Orzalli *et al*, 2021), it will be vital to assess the role of A1 in infectious disease, particularly as it is conceivable that anti‐cancer A1/BFL‐1 inhibitors in development (Li *et al*, 2021) could be re‐purposed as antimicrobial therapeutics.

## Materials and Methods

### Mice

All mice were housed under standard conditions at the Walter + Eliza Hall Institute of Medical Research (WEHI), Australia, and Monash University, Australia. All procedures were performed in accordance with the National Health and Medical Research Council Australian Code of Practice for the Care and Use of Animals and approved by the WEHI (2017.026, 2020.038) or Monash Animal Ethics Committees (MARP/2016/099, MMCB/2019/28‐BC, MMCB/2021/25). Male and female C57BL/6 wild‐type (WT), A1‐deficient (*A1* ^−/−^) (Schenk *et al*, 2017) and GSDMD‐deficient (Kayagaki *et al*, 2015) mice at > 6 weeks of age were used for *in vitro* generation of bone marrow‐derived macrophages (BMDMs) or monocytes. For *in vivo* experiments, 8–12‐week‐old male WT and *A1* ^−/−^ mice were injected intraperitoneally with 100 μg of NOMVs or 100 μg LPS. Mice were euthanised after 5–6 h (h) and cardiac bled for FACS analysis, differential blood counts (ADVIA 120 cell analyser, Bayer Diagnostics), and serum isolation. Peritoneal lavages were performed using ice cold 5 mM EDTA in PBS and fluid collected following centrifugation. Serum and peritoneal lavage fluid were stored at −80°C prior to cytokine analysis by ELISA and immunoblot.

### Purification of outer membrane vesicles (OMVs) from *N. gonorrhoeae*

The *N*. *gonorrhoeae* MS11‐A strain was cultured in gonococcal (GC) liquid media supplemented with Deakin modified isovitalex (DMIV) and 0.01 M NaHCO_3_ at 37°C, shaking at 200 rpm. Bacterial cultures were grown to an optical density (OD_600_) of ~0.5 (mid‐log phase), diluted in fresh media (1:1,000 dilution), and incubated until an OD_600_ ~ 0.8 was reached. Bacteria were removed by centrifugation (12,000 × *g* for 12 min at 4°C), and the spent culture medium was harvested and filtered (using 0.45 μm nitrocellulose MF‐Millipore™ membrane filters) before NOMVs were isolated by density gradient ultracentrifugation, as detailed in (Deo *et al*, 2018). NOMVs were quantified using a bicinchoninic acid assay (Pierce BCA protein assay kit, Thermo Scientific CST #23225).

### Cell culture

To generate BMDM and monocyte cultures, cells were harvested from the tibial and femoral bones. For BMMo cultures, a red cell lysis was performed before the bone marrow was transferred to a T75 flask overnight to remove adherent stromal cells. All cells were cultured in DMEM (Gibco) containing 10% foetal bovine serum (FBS; Bovogen), 15–20% L929 cell‐conditioned media (LCCM), 4 mM L‐glutamine (Life Technologies), 1 mM sodium pyruvate (Thermofisher), and 100 U/ml penicillin/streptomycin (Life Technologies) at 37°C, 10% CO_2_ for 4 days to generate BMMo (Francke *et al*, 2011) or 6 days for BMDMs. LCCM‐derived BMMo and primary sorted Ly6C^hi^ monocytes were routinely plated at 0.75–2.5 × 10^5^ cells/well, in 96‐well flat‐bottom tissue culture‐treated plates (BD Falcon) in DMEM (Gibco) containing 10% FBS, 1 mM sodium pyruvate (Thermofisher), and 100 U/ml penicillin/streptomycin (Life Technologies) for relevant assays. Unless otherwise indicated, macrophages were plated at 4 × 10^5^ cells/well in 24‐well tissue culture‐treated plates or 1 × 10^5^ cells/well in 96‐well flat bottom tissue culture‐treated plates (BD Falcon or Greiner). Alternatively, BMDMs were plated at 3 × 10^5^ in 24‐well non‐tissue culture‐treated plates (Corning or BD Falcon) for flow cytometric analyses. Where indicated, cells were primed with 50 ng/ml B4 (*E. coli* O111:B4) or B5 (*E. coli* O55:B5) LPS (Ultrapure, InvivoGen) for 3–4 h (or up to 24 h for time courses), pre‐treated, as indicated, for the last 15–30 min of priming with MG132 (5 μM, SIGMA‐Aldrich), Bafilomycin A (100 nM, Jomar Life Science), MCC950 (5 μM, CP‐456773 sodium salt, PZ0280), Q‐VD‐OPh (40 μM, *In Vitro* Technologies), GSK'872 (1 μM, MedChemExpress), z‐VAD‐fmk (50 μM, *In Vitro* Technologies), rat anti‐mouse TNF (XT22, 20 μg/ml, WEHI Monoclonal Antibody Facility) and rat anti‐mouse IgG1 (isotype control GL113, 20 μg/ml, WEHI Monoclonal Antibody Facility) monoclonal antibodies (mAb), and incubated with cycloheximide (CHX, 20 μg/ml, SIGMA‐Aldrich), ABT‐737 (500 nM, Active Biochem), ABT‐199 (1 μM, Active Biochem), A‐1331852 (1 μM, Chemgood), S63845 (10 μM, Active Biochem) and nigericin (10 μM) for specified times. Alternatively, BMMo and sorted Ly6C^hi^ monocytes were pre‐treated with specified inhibitors for 15–30 min and NOMVs (50 μg/ml) added for specified times. For activation of the noncanonical Caspase‐11 inflammasome, cells were primed for 12 h with 500 ng/ml Pam_3_Cys before media was removed and replaced with Optimem supplemented with 50 ng/ml M‐CSF (Peprotech). Cells were transfected with 12.5 μg/ml ultrapure K12 *E. coli* LPS (0.313% FuGENE HD Transfection Reagent; Promega) for the indicated times. Supernatants and cells were routinely harvested from assays at specified times for cell death analysis by FACS. Cell supernatants and lysates were also routinely prepared to measure inflammatory cytokine, lactate dehydrogenase (LDH) release or cell death pathway activation by ELISA, colorimetric assay and immunoblotting, respectively.

### Ly6C ^+^ monocyte isolation

To obtain primary Ly6C^hi^ monocytes for cell death and cytokine analysis, as performed previously (Lawlor *et al*, 2015), bone marrow was harvested from the tibial, femoral, and hip bones of 6–16‐week‐old mice and stained with PI and the following antibodies: anti‐CD11b (PE‐Cy7; M1/70; eBioscience), anti‐Ly6C (APC‐Cy7; HK1.4; BioLegend), and anti‐Ly6G (V450: 1A8; eBioscience). Monocytes [PI^−^, CD11b^+^Ly6G^−^Ly6C^hi^] were sorted using a BD FACS Aria Fusion instrument with a 70 μm nozzle. Ly6C^+^ monocytes (~80% purity) for the cytochrome‐*c* release assay and time course westerns were isolated directly from the bone marrow using the EasySep™ Mouse Monocyte Isolation kit (Stem Cell Technologies) according to the manufacturers' instructions.

### *Salmonella* infection

*Salmonella enterica* Typhimurium type strain SL1344 was plated onto Luria Bertani (LB) agar containing 50 μg/ml streptomycin (Sigma) and incubated at 37°C overnight. Single colonies were inoculated into 10 ml LB broth (+50 μg/ml streptomycin) and incubated in a 37°C shaking incubator at ~200 rpm for 16 h, then sub‐cultured 1/50 in 5 ml LB broth, as above. Bacterial cell numbers were determined by absorbance at OD_600_ and standardised to a multiplicity of infection (MOI) of 10 in un‐supplemented DMEM. The infection protocol used is described in detail in (Ingle *et al*, 2021). Briefly, BMDMs were infected with SL1344 (or DMEM alone) at MOI:10 and centrifuged at 525 × *g* to synchronise bacterial uptake, then incubated at 37°C in 5% CO_2_ for 30 min. Infective media was removed, and cells were washed and incubated with DMEM containing 100 μg/ml gentamicin for 1 h to inhibit extracellular bacterial growth. Finally, this media was replaced with DMEM containing 20% LCCM, 10% FBS, and 10 μg/ml gentamicin for the remainder of the experiment. Cell supernatants were harvested at 0, 3, 9 and 24 h post‐infection (h.p.i.) for cytokine and LDH release (i.e., cell death). To quantify intracellular bacteria, the cell culture media was removed and BMDMs were washed 2× with warm PBS to remove extracellular bacteria, then lysed in 0.1% Triton X‐100 by repetitive pipetting. Intracellular lysates were then serially diluted in PBS in duplicate, plated onto LB agar (+50 μg/ml streptomycin), and incubated overnight at 37°C.

### Flow cytometry

All flow cytometric analysis was performed on a BD LSR‐Fortessa X20 instrument with Diva software (BD Biosciences). Data were analysed using FlowJo software version 10.6.1 or Weasel software version 3.4 (purchased from Frank Battye). To assess cell viability, semi‐adherent and adherent cells were harvested using 5 mM EDTA in phosphate buffered saline (PBS) (~3–5 min incubation, room temperature) and pooled in FACS tubes with non‐adherent cells, before staining with 1–2 μg/ml propidium iodide (PI) (Sigma‐Aldrich). For monocyte culture population analysis, cells were washed 2× in PBS, and FcγRIIb/III blocked using anti‐CD16/CD32 (2.4G2; BD Biosciences), and cells stained using the following reagents: Zombie‐NIR fixable viability kit (Biolegend), anti‐B220 (PE; RA3‐6B2; BD Bioscience), anti‐CD11b (PE‐Cy7; M1/70; eBioscience), anti‐Ly6C (APC‐Cy7; HK1.4; BioLegend), anti‐Ly6G (AlexaFluor v450; 1A8; eBioscience), and anti‐F4/80 (FITC; eBioscience). For analysis of blood leukocytes following NOMV challenge, red blood cells from a recorded volume of blood (< 150 μl) collected via cardiac bleed with 2 μl 0.5 M EDTA were lysed in 1 ml 1× red cell removal buffer (made in‐house). Cells were washed in FACS buffer (PBS + 3% FCS + 2.5 mM EDTA), and FcγRIIb/III blocked using anti‐CD16/CD32 (2.4G2; BD Biosciences), before staining with the following antibodies: anti‐B220 (BV711; RA3‐6B2; BD Bioscience), anti‐CD3ε (FITC; 145‐2C11; BD Bioscience), anti‐CD4 (BV605; RM4‐5; BD Bioscience), anti‐CD8α (BV650; 53‐6.7; BD Bioscience), anti‐CD11b (PE‐Cy7; M1/70; eBioscience), anti‐Ly6C (APC‐Cy7; HK1.4; BioLegend), anti‐Ly6G (eFluor450; 1A8; eBioscience). Cells were washed then resuspended in FACS buffer + 1 μg/ml Fluorogold with CountBright Absolute Counting Beads (Life Technologies, C36950) added prior to acquisition.

### Caspase activity assay

To assess caspase‐3 activity, 1 × 10^5^ BMDMs were plated per well in 96‐well flat‐bottom tissue culture‐treated plates (BD Falcon), primed with LPS (50 ng/ml) for 3 h and then treated, as indicated with Q‐VD‐OPh (40 μM), ABT‐737 (500 nM) and S63845 (10 μM). All stimulations were staggered in a reverse time course fashion to ensure identical LPS priming and QVD incubation periods across treatments. At experimental endpoint, supernatants were discarded after cell centrifugation and BMDMs lysed in 70 μl DISC lysis buffer (20 mM Tris, 150 mM NaCl, 2 mM EDTA, 1% TritonX‐100, 10% Glycerol, H_2_O) for 1 h at room temperature with rotation. 10 μl of cell lysate was used for protein quantification (Pierce™ BCA Protein Assay Kit, Thermo Fisher Scientific) according to manufacturer's instruction, and 50 μl was transferred to an opaque‐walled, clear bottom 96‐well flat bottom plate. DEVDase substrate (Ac‐DEVD‐AMC, BD Pharmingen™ Cat) was prepared to working concentration (20 μM) in assay buffer (20 mM HEPES pH 7.5, 10% Glycerol, 2 mM Dithiothreitol (DTT)). 200 μl of prepared DEVDase substrate reagent was added to each well containing lysates and allowed to incubate overnight with rotation in the absence of light. After incubation, caspase AMC fluorescence was detected using a CLARIOstar Plus (BMG Labtech). Protein concentrations obtained from the BCA were used to normalise the AMC fluorescence intensity across samples.

### Cytochrome‐*c* release assay

Cytochrome‐*c* release was measured under relevant specified conditions (see figure legends for details), as performed previously (Waterhouse & Trapani, 2003). Briefly, either unstained BMMo or macrophages, or Ly6C^+^‐stained monocytes were washed 1× in PBS and resuspended in 50 μl MELB buffer (20 mM HEPES pH 7.5, 100 mM sucrose, 2.5 mM MgCl_2_, 100 mM KCl) supplemented with 0.025% w/v digitonin and complete protease inhibitors (Roche) for 10 min on ice to allow permeabilisation of the plasma membrane. Cells were then washed with MELB buffer before fixation in 50 μl eBioscience fixation buffer for 30 min on ice. Cells were washed 1× with eBioscience permeabilization buffer before incubation with an APC‐conjugated anti‐cytochrome‐*c* antibody (REA702; Miltenyi Biotech) or APC‐conjugated isotype control (REA293; Miltenyi Biotech) for 30 min on ice. Cells were washed 1× before data acquisition on a BD LSR‐Fortessa X20 instrument and analysis using FlowJo.

### Live‐Cell imaging

To assess the kinetics and modes of cell death induced in LPS‐primed BMDMs in response to BH3‐mimetics, 5 × 10^4^ macrophages were seeded into wells of a 96‐well Thermo Scientific™ Nunc™ Edge 2.0 plate. After an overnight incubation to allow cell adhesion, culture medium was replaced with fresh DMEM (supplemented with 20% LCCM, 4 mM L‐glutamine, 1 mM sodium pyruvate, and 100 U/ml penicillin/streptomycin) containing PI (200 ng/ml), SPY505‐DNA (1×, Spirochrome) and, where indicated, LPS (50 ng/ml). After 2.5 h cells were pre‐treated, as indicated, with Q‐VD‐OPh (40 μM, *In Vitro* Technologies), MCC950 (5 μM, CP‐456773 sodium salt, PZ0280), and/or GSK'872 (5 μM, MedChemExpress), rat anti‐mouse TNF (XT‐22 20 μg/ml) or rat anti‐mouse IgG1 (GL113 Isotype control) monoclonal antibodies for 30 min prior to treatment with ABT‐737 (500 nM) and S63845 (10 μM). To assess the kinetics of NOMV‐induced monocyte cell death, sorted Ly6C^hi^ monocytes were labelled with 100 nM Cell Tracker Green (CTG) (Invitrogen) for 10 min at 37°C in serum‐free DMEM. Cells were then washed and seeded at 0.5–1 × 10^5^ cells/well in 96‐well flat bottom tissue culture‐treated plates (Greiner). Monocyte cultures were pre‐treated, as indicated, for 15–30 min with Q‐VD‐OPh (40 μM), MCC950 (5 μM), and GSK'872 (1 μM), prior to treatment with 50 μg/ml of NOMVs. Monocytes were then exposed to 200 ng/ml PI and imaged every 30 min on an IncuCyte® S3 or SX5 Live‐Cell Analysis System (Sartorius) at 10× magnification for up to 14 h. BMDM and monocyte images were analysed, and the % of dead cells was calculated by dividing the number of PI‐positive cells by the total cell number (based on CTG^+^ or SPY505‐DNA^+^ cells), as quantified by the IncuCyte® Analysis Software.

### RNA sequencing preparation and analysis

3′ mRNA sequencing of LPS‐treated versus untreated BMDMs was performed as described in (Simpson *et al*, 2022). Total RNA was extracted using the ISOLATE II RNA Mini Kit (Meridian Bioscience). The extracted RNA was analysed on the Agilent 4200 Tapestation prior to library preparation. 3′ mRNA‐sequencing libraries were prepared using the QuantSeq 3′ mRNA‐Seq Library Prep (Lexogen) according to the manufacturer's instructions and sequenced on the NextSeq 500 (Illumina). The single‐end 75 bp were demultiplexed using Casavav1.8.2. Cutadapt (v1.9) was used for read trimming (Martin, 2011). The trimmed reads were subsequently mapped to the mouse genome (mm10) using HISAT2 (Kim *et al*, 2019). FeatureCounts from Rsubread package (version 1.34.7) was used for read counting after which genes with less than 2 counts per million reads (CPM) in at least 3 samples were excluded from downstream analysis (Liao *et al*, 2014, 2019). Count data were normalised using the trimmed mean of M values (TMM) method and differential gene expression analysis was performed using the *limma‐voom* pipeline (limma version 3.40.6) (Robinson & Oshlack, 2010; Law *et al*, 2014; Liao *et al*, 2014). Gene ontology (GO) analysis was performed using *Metascape*. Heatmaps were generated using *pheatmap* (version 1.0.12) (https://rdrr.io/cran/pheatmap/). The datasets generated during this study are available at GEO: GSE214525.

### Quantitative RT‐PCR

2 × 10^6^ BMDMs were treated with 50 ng/ml LPS for up to 24 h before total RNA was extracted and purified using the Isolate II RNA mini kit (Bioline) or RNeasy Mini kit (Qiagen). RNA concentrations were quantified and standardised using a Nanodrop™ spectrophotometer. RNA (1 μg) was transcribed using the high‐capacity cDNA reverse transcription kit (Applied Biosystems). The cDNA was then diluted 1:10 and qRT‐PCR was performed on a QuantStudio 6 Flex PCR system with 2 μl of diluted cDNA, SYBR green reagent (Applied Biosystems), and the primer pairs listed below. Relative mRNA levels were calculated using the comparative delta–delta C_t_ (2^−ΔΔCt^) method (2 − [(dCt LPS given hour) − (dCT LPS 0 h)]) after gene expression was normalised to an internal house‐keeping reference gene *18S*. Results are displayed as the relative fold of mRNA levels compared to no LPS (0 h) treatment. Primer sequences used to assay relevant murine gene expression are as follows: *18S* (For: 5′gtaacccgttgaaccccatt Rev: 3′ccatccaatcggtagtagcg) *Bcl2a1* (For: 5′tccacaagagcagattgccctg Rev: 3′gccagccagatttgggttcaaac) *Mcl1* (For: 5′agcttcatcgaaccattagcagaa Rev: 3′ccttctaggtcctgtacgtgga) *Bcl2l1* (For: 5′gccacctatctgaatgaccacc Rev: 3′aggaaccagcggttgaagcgc). The specificity of each primer set was confirmed by the observation of a single peak in the melt curve graph of each qPCR run.

### Cytokine analysis in supernatants and serum

IL‐1β (R&D), TNF (eBioscience), and IL‐6 (eBioscience) ELISA kits were used according to the manufacturers' instructions. For detection of TNF in cell supernatants, or IL‐6 in serum, samples were diluted 1:10 in assay diluent.

### LDH assay

Supernatants were removed from cells and pelleted at 1,500 rpm to remove any debris. Control wells were left untreated or lysed in 1% Triton X‐100 (Tx, Sigma‐Aldrich, T9284) to establish maximum LDH release. % LDH activity was analysed using the Cytotoxicity Detection Kit (LDH), according to the manufacturer's instructions (Roche; 11644793001). Absorbance was measured at 490 nm and % cytotoxicity calculated as: [(experimental value − untreated control)/(Tx lysed control − untreated control)].

### Immunoblotting

Before loading, supernatants and cell lysates were boiled for 10 min in 1× NuPAGE LDS (Thermofisher) or in‐house (2% w/v SDS, 10% v/v glycerol, 50 mM Tris pH 6.8, 0.01% bromophenol blue) sample buffer containing 5% β‐mercaptoethanol (β‐ME). For serum blots, 4 μl of serum was diluted in sample buffer and run per lane. Cell lysates and supernatants (reduced and denatured) were separated on 4–12% Bis‐Tris gradient gels (Invitrogen) and proteins transferred onto nitrocellulose (Millipore) or PVDF (Thermofisher) membranes. Ponceau staining was used to confirm protein transfer and as a loading control. Membranes were blocked with 5% skim milk in TBS or PBS + 0.1% Tween‐20 for 30 min, and then probed overnight at 4°C with the following primary antibodies (all diluted 1:1,000 + 0.02% sodium azide, unless otherwise stated): A1 (clone 6D6; WEHI antibody facility) (Lang *et al*, 2014), BCL‐xL (CST; 2764S), MCL‐1 (CST; 5453S), pro‐ and cleaved IL‐1β (R&D; AF‐401‐NA), cIAP1 (1:500, Enzo; ALX‐803‐335), XIAP (MBL; M044‐3), pro‐ and cleaved caspase‐1 (Adipogen; AG‐20B‐0042‐C100), pro‐caspase‐8 (WEHI, clone 3B10), cleaved caspase‐8 (CST; 9429S), caspase‐3 (CST; 9662S), cleaved caspase‐3 (CST; 9661S), FLIP (Adipogen; AG‐20B‐0005‐C100), GSDMD (Abcam; ab209845), GSDME (Abcam; ab215191), PARP (CST; 9542) and HRP‐conjugated *β*‐actin (CST; 5125S). The following day, membranes were washed 4–6× in TBS or PBS + 0.1% Tween‐20 before the relevant horseradish peroxidase (HRP)‐conjugated secondary antibodies (all diluted 1:5,000) were applied for 1 h at room temperature. Membranes were washed and then developed using the Immobilon Forte Western HRP substrate (Merck) and imaged with a BioRad ChemiDoc MP or an Amersham Imager 680 Blot and Gel Imager. Images were analysed and processed with BioRad ImageLab software, Adobe Photoshop, and Adobe Illustrator.

### Statistical analysis

Flow cytometric data were analysed using FlowJo version 10 software. Error bars (SEM or SD) were calculated using Prism 8.1, as indicated in the figure legends. The number of times each experiment was repeated, and the number of animals used per experiment, are detailed in the figure legends. For *in vitro* analyses or *in vivo* analyses, a one‐way or two‐way ANOVA with Tukey's multiple comparisons test was performed to calculate significance. Normal distribution with similar variance between groups was assumed. For *in vivo* analyses of serum cytokines between 2 groups, an unpaired, two‐tailed Student's *t*‐test was performed to calculate statistical significance. No samples were excluded from analyses and no statistical methods were used to calculate sample size.

## Author contributions

**Mary Speir:** Data curation; formal analysis; funding acquisition; investigation; methodology; writing – original draft; writing – review and editing. **Hazel Tye:** Data curation; formal analysis; investigation; methodology; writing – review and editing. **Timothy A Gottschalk:** Data curation; formal analysis; investigation; methodology; writing – review and editing. **Daniel S Simpson:** Data curation; formal analysis; investigation; methodology; writing – review and editing. **Tirta M Djajawi:** Data curation; formal analysis; investigation; methodology; writing – review and editing. **Pankaj Deo:** Formal analysis; investigation; methodology; writing – review and editing. **Rebecca L Ambrose:** Data curation; formal analysis; investigation; methodology; writing – review and editing. **Stephanie A Conos:** Formal analysis; funding acquisition; investigation; writing – review and editing. **Jack Emery:** Investigation. **Gilu Abraham:** Investigation. **Ashlyn Pascoe:** Investigation. **Sebastian A Hughes:** Investigation. **Ashley Weir:** Investigation. **Edwin D Hawkins:** Resources; supervision; funding acquisition. **Isabella Kong:** Data curation; formal analysis; investigation; methodology; writing – review and editing. **Marco J Herold:** Resources; funding acquisition; writing – review and editing. **Jaclyn S Pearson:** Resources; supervision; funding acquisition; writing – review and editing. **Najoua Lalaoui:** Data curation; formal analysis; investigation; methodology; writing – review and editing. **Thomas Naderer:** Resources; supervision; funding acquisition; writing – original draft; writing – review and editing. **James E Vince:** Data curation; formal analysis; supervision; investigation; methodology; writing – original draft; writing – review and editing. **Kate E Lawlor:** Conceptualization; resources; data curation; formal analysis; supervision; funding acquisition; investigation; methodology; writing – original draft; project administration; writing – review and editing.

## Disclosure and competing interests statement

The authors declare that HT, DSS, TMD, AW, SAH, EDH, IK, MJH, NL, JEV and KEL are currently or were employed at the Walter + Eliza Hall Medical Institute, which receives milestone payments from Genentech and AbbVie for the development of ABT‐199 for cancer therapy. All other authors declare no competing interests.

## Supporting information



Appendix

Expanded View Figures PDF

Source Data for Expanded View and Appendix

PDF+

Source Data for Figure 1

Source Data for Figure 2

Source Data for Figure 3

Source Data for Figure 4

Source Data for Figure 5

Source Data for Figure 6

## Data Availability

3′ mRNA sequencing datasets generated during this study are available at GEO: GSE214525 (https://www.ncbi.nlm.nih.gov/geo/query/acc.cgi?acc=GSE214525).

## References

[embr202356865-bib-0001] Allam R , Lawlor KE , Yu EC , Mildenhall AL , Moujalled DM , Lewis RS , Ke F , Mason KD , White MJ , Stacey KJ *et al* (2014) Mitochondrial apoptosis is dispensable for NLRP3 inflammasome activation but non‐apoptotic caspase‐8 is required for inflammasome priming. EMBO Rep 15: 982–990 24990442 10.15252/embr.201438463 PMC4198042

[embr202356865-bib-0002] Bossaller L , Chiang PI , Schmidt‐Lauber C , Ganesan S , Kaiser WJ , Rathinam VA , Mocarski ES , Subramanian D , Green DR , Silverman N *et al* (2012) Cutting edge: FAS (CD95) mediates noncanonical IL‐1beta and IL‐18 maturation via caspase‐8 in an RIP3‐independent manner. J Immunol 189: 5508–5512 23144495 10.4049/jimmunol.1202121 PMC3518757

[embr202356865-bib-0003] Brinkmann K , Grabow S , Hyland CD , Teh CE , Alexander WS , Herold MJ , Strasser A (2017) The combination of reduced MCL‐1 and standard chemotherapeutics is tolerable in mice. Cell Death Differ 24: 2032–2043 28800129 10.1038/cdd.2017.125 PMC5686343

[embr202356865-bib-0004] Broz P , Dixit VM (2016) Inflammasomes: mechanism of assembly, regulation and signalling. Nat Rev Immunol 16: 407–420 27291964 10.1038/nri.2016.58

[embr202356865-bib-0005] Brumatti G , Ma C , Lalaoui N , Nguyen NY , Navarro M , Tanzer MC , Richmond J , Ghisi M , Salmon JM , Silke N *et al* (2016) The caspase‐8 inhibitor emricasan combines with the SMAC mimetic birinapant to induce necroptosis and treat acute myeloid leukemia. Sci Transl Med 8: 339ra369 10.1126/scitranslmed.aad3099 27194727

[embr202356865-bib-0006] Bulanova D , Ianevski A , Bugai A , Akimov Y , Kuivanen S , Paavilainen H , Kakkola L , Nandania J , Turunen L , Ohman T *et al* (2017) Antiviral properties of chemical inhibitors of cellular anti‐apoptotic Bcl‐2 proteins. Viruses 9: 271 28946654 10.3390/v9100271 PMC5691623

[embr202356865-bib-0007] Chauhan D , Bartok E , Gaidt MM , Bock FJ , Herrmann J , Seeger JM , Broz P , Beckmann R , Kashkar H , Tait SWG *et al* (2018) BAX/BAK‐induced apoptosis results in caspase‐8‐dependent IL‐1beta maturation in macrophages. Cell Rep 25: 2354–2368 30485805 10.1016/j.celrep.2018.10.087

[embr202356865-bib-0008] Chen KW , Demarco B , Heilig R , Shkarina K , Boettcher A , Farady CJ , Pelczar P , Broz P (2019) Extrinsic and intrinsic apoptosis activate pannexin‐1 to drive NLRP3 inflammasome assembly. EMBO J 38: e101638 30902848 10.15252/embj.2019101638 PMC6517827

[embr202356865-bib-0009] Coll RC , Robertson AA , Chae JJ , Higgins SC , Munoz‐Planillo R , Inserra MC , Vetter I , Dungan LS , Monks BG , Stutz A *et al* (2015) A small‐molecule inhibitor of the NLRP3 inflammasome for the treatment of inflammatory diseases. Nat Med 21: 248–255 25686105 10.1038/nm.3806 PMC4392179

[embr202356865-bib-0010] Conos SA , Chen KW , De Nardo D , Hara H , Whitehead L , Núñez G , Masters SL , Murphy JM , Schroder K , Vaux DL *et al* (2017) Active MLKL triggers the NLRP3 inflammasome in a cell‐intrinsic manner. Proc Natl Acad Sci USA 114: E961–E969 28096356 10.1073/pnas.1613305114 PMC5307433

[embr202356865-bib-0011] Csepregi JZ , Orosz A , Zajta E , Kasa O , Nemeth T , Simon E , Fodor S , Csonka K , Baratki BL , Kovesdi D *et al* (2018) Myeloid‐specific deletion of Mcl‐1 yields severely neutropenic mice that survive and breed in homozygous form. J Immunol 201: 3793–3803 30464050 10.4049/jimmunol.1701803 PMC6287103

[embr202356865-bib-0012] Cunha LD , Zamboni DS (2013) Subversion of inflammasome activation and pyroptosis by pathogenic bacteria. Front Cell Infect Microbiol 3: 76 24324933 10.3389/fcimb.2013.00076 PMC3840304

[embr202356865-bib-0013] Dang EV , McDonald JG , Russell DW , Cyster JG (2017) Oxysterol restraint of cholesterol synthesis prevents AIM2 inflammasome activation. Cell 171: 1057–1071 29033131 10.1016/j.cell.2017.09.029 PMC5693620

[embr202356865-bib-0014] Deo P , Chow SH , Hay ID , Kleifeld O , Costin A , Elgass KD , Jiang JH , Ramm G , Gabriel K , Dougan G *et al* (2018) Outer membrane vesicles from *Neisseria gonorrhoeae* target PorB to mitochondria and induce apoptosis. PLoS Pathog 14: e1006945 29601598 10.1371/journal.ppat.1006945 PMC5877877

[embr202356865-bib-0015] Deo P , Chow SH , Han ML , Speir M , Huang C , Schittenhelm RB , Dhital S , Emery J , Li J , Kile BT *et al* (2020) Mitochondrial dysfunction caused by outer membrane vesicles from Gram‐negative bacteria activates intrinsic apoptosis and inflammation. Nat Microbiol 5: 1418–1427 32807891 10.1038/s41564-020-0773-2

[embr202356865-bib-0016] Dhital S , Deo P , Stuart I , Naderer T (2021) Bacterial outer membrane vesicles and host cell death signaling. Trends Microbiol 29: 1106–1116 34001418 10.1016/j.tim.2021.04.003

[embr202356865-bib-0017] Doerflinger M , Deng Y , Whitney P , Salvamoser R , Engel S , Kueh AJ , Tai L , Bachem A , Gressier E , Geoghegan ND *et al* (2020) Flexible usage and interconnectivity of diverse cell death pathways protect against intracellular infection. Immunity 53: 533–547 32735843 10.1016/j.immuni.2020.07.004 PMC7500851

[embr202356865-bib-0018] Dzhagalov I , St John A , He YW (2007) The antiapoptotic protein Mcl‐1 is essential for the survival of neutrophils but not macrophages. Blood 109: 1620–1626 17062731 10.1182/blood-2006-03-013771 PMC1794052

[embr202356865-bib-0019] Ekert PG , Read SH , Silke J , Marsden VS , Kaufmann H , Hawkins CJ , Gerl R , Kumar S , Vaux DL (2004) Apaf‐1 and caspase‐9 accelerate apoptosis, but do not determine whether factor‐deprived or drug‐treated cells die. J Cell Biol 165: 835–842 15210730 10.1083/jcb.200312031 PMC2172390

[embr202356865-bib-0020] Eng VV , Wemyss MA , Pearson JS (2020) The diverse roles of RIP kinases in host‐pathogen interactions. Semin Cell Dev Biol 109: 125–143 32859501 10.1016/j.semcdb.2020.08.005 PMC7448748

[embr202356865-bib-0021] Erlich Z , Shlomovitz I , Edry‐Botzer L , Cohen H , Frank D , Wang H , Lew AM , Lawlor KE , Zhan Y , Vince JE *et al* (2019) Macrophages, rather than DCs, are responsible for inflammasome activity in the GM‐CSF BMDC model. Nat Immunol 20: 397–406 30742078 10.1038/s41590-019-0313-5

[embr202356865-bib-0022] Feng S , Yang Y , Mei Y , Ma L , Zhu DE , Hoti N , Castanares M , Wu M (2007) Cleavage of RIP3 inactivates its caspase‐independent apoptosis pathway by removal of kinase domain. Cell Signal 19: 2056–2067 17644308 10.1016/j.cellsig.2007.05.016

[embr202356865-bib-0023] Francke A , Herold J , Weinert S , Strasser RH , Braun‐Dullaeus RC (2011) Generation of mature murine monocytes from heterogeneous bone marrow and description of their properties. J Histochem Cytochem 59: 813–825 21705645 10.1369/0022155411416007 PMC3201167

[embr202356865-bib-0024] Fritsch M , Gunther SD , Schwarzer R , Albert MC , Schorn F , Werthenbach JP , Schiffmann LM , Stair N , Stocks H , Seeger JM *et al* (2019) Caspase‐8 is the molecular switch for apoptosis, necroptosis and pyroptosis. Nature 575: 683–687 31748744 10.1038/s41586-019-1770-6

[embr202356865-bib-0025] Gaidt MM , Ebert TS , Chauhan D , Schmidt T , Schmid‐Burgk JL , Rapino F , Robertson AA , Cooper MA , Graf T , Hornung V (2016) Human monocytes engage an alternative inflammasome pathway. Immunity 44: 833–846 27037191 10.1016/j.immuni.2016.01.012

[embr202356865-bib-0026] Gangoda L , Schenk RL , Best SA , Nedeva C , Louis C , D'Silva DB , Fairfax K , Jarnicki AG , Puthalakath H , Sutherland KD *et al* (2021) Absence of pro‐survival A1 has no impact on inflammatory cell survival *in vivo* during acute lung inflammation and peritonitis. Cell Death Differ 1: 96–104 10.1038/s41418-021-00839-3 PMC8738744 34304242

[embr202356865-bib-0027] Giampazolias E , Zunino B , Dhayade S , Bock F , Cloix C , Cao K , Roca A , Lopez J , Ichim G , Proics E *et al* (2017) Mitochondrial permeabilization engages NF‐kappaB‐dependent anti‐tumour activity under caspase deficiency. Nat Cell Biol 19: 1116–1129 28846096 10.1038/ncb3596 PMC5624512

[embr202356865-bib-0028] Goodall KJ , Finch‐Edmondson ML , van Vuuren J , Yeoh GC , Gentle IE , Vince JE , Ekert PG , Vaux DL , Callus BA (2016) Cycloheximide can induce Bax/Bak dependent myeloid cell death independently of multiple BH3‐only proteins. PLoS One 11: e0164003 27806040 10.1371/journal.pone.0164003 PMC5091851

[embr202356865-bib-0029] Herold MJ , Zeitz J , Pelzer C , Kraus C , Peters A , Wohlleben G , Berberich I (2006) The stability and anti‐apoptotic function of A1 are controlled by its C terminus. J Biol Chem 281: 13663–13671 16551634 10.1074/jbc.M600266200

[embr202356865-bib-0030] Inde Z , Croker BA , Yapp C , Joshi GN , Spetz J , Fraser C , Qin X , Xu L , Deskin B , Ghelfi E *et al* (2021) Age‐dependent regulation of SARS‐CoV‐2 cell entry genes and cell death programs correlates with COVID‐19 severity. Sci Adv 7: eabf8609 34407940 10.1126/sciadv.abf8609 PMC8373124

[embr202356865-bib-0031] Ingle DJ , Ambrose RL , Baines SL , Duchene S , Goncalves da Silva A , Lee DYJ , Jones M , Valcanis M , Taiaroa G , Ballard SA *et al* (2021) Evolutionary dynamics of multidrug resistant *Salmonella enterica* serovar 4,[5],12:i:‐ in Australia. Nat Commun 12: 4786 34373455 10.1038/s41467-021-25073-w PMC8352879

[embr202356865-bib-0032] Jorgensen I , Rayamajhi M , Miao EA (2017) Programmed cell death as a defence against infection. Nat Rev Immunol 17: 151–164 28138137 10.1038/nri.2016.147 PMC5328506

[embr202356865-bib-0033] Kang TB , Yang SH , Toth B , Kovalenko A , Wallach D (2013) Caspase‐8 blocks kinase RIPK3‐mediated activation of the NLRP3 inflammasome. Immunity 38: 27–40 23260196 10.1016/j.immuni.2012.09.015

[embr202356865-bib-0034] Kayagaki N , Stowe IB , Lee BL , O'Rourke K , Anderson K , Warming S , Cuellar T , Haley B , Roose‐Girma M , Phung QT *et al* (2015) Caspase‐11 cleaves gasdermin D for non‐canonical inflammasome signalling. Nature 526: 666–671 26375259 10.1038/nature15541

[embr202356865-bib-0035] Kim D , Paggi JM , Park C , Bennett C , Salzberg SL (2019) Graph‐based genome alignment and genotyping with HISAT2 and HISAT‐genotype. Nat Biotechnol 37: 907–915 31375807 10.1038/s41587-019-0201-4 PMC7605509

[embr202356865-bib-0036] Kotschy A , Szlavik Z , Murray J , Davidson J , Maragno AL , Le Toumelin‐Braizat G , Chanrion M , Kelly GL , Gong JN , Moujalled DM *et al* (2016) The MCL1 inhibitor S63845 is tolerable and effective in diverse cancer models. Nature 538: 477–482 27760111 10.1038/nature19830

[embr202356865-bib-0037] Kucharczak JF , Simmons MJ , Duckett CS , Gelinas C (2005) Constitutive proteasome‐mediated turnover of Bfl‐1/A1 and its processing in response to TNF receptor activation in FL5.12 pro‐B cells convert it into a prodeath factor. Cell Death Differ 12: 1225–1239 16094403 10.1038/sj.cdd.4401684

[embr202356865-bib-0038] Lalaoui N , Boyden SE , Oda H , Wood GM , Stone DL , Chau D , Liu L , Stoffels M , Kratina T , Lawlor KE *et al* (2020) Mutations that prevent caspase cleavage of RIPK1 cause autoinflammatory disease. Nature 577: 103–108 31827281 10.1038/s41586-019-1828-5 PMC6930849

[embr202356865-bib-0039] Lang MJ , Brennan MS , O'Reilly LA , Ottina E , Czabotar PE , Whitlock E , Fairlie WD , Tai L , Strasser A , Herold MJ (2014) Characterisation of a novel A1‐specific monoclonal antibody. Cell Death Dis 5: e1553 25476901 10.1038/cddis.2014.519 PMC4649835

[embr202356865-bib-0040] Law CW , Chen Y , Shi W , Smyth GK (2014) voom: precision weights unlock linear model analysis tools for RNA‐seq read counts. Genome Biol 15: R29 24485249 10.1186/gb-2014-15-2-r29 PMC4053721

[embr202356865-bib-0041] Lawlor KE , Vince JE (2014) Ambiguities in NLRP3 inflammasome regulation: is there a role for mitochondria? Biochim Biophys Acta 1840: 1433–1440 23994495 10.1016/j.bbagen.2013.08.014

[embr202356865-bib-0042] Lawlor KE , Khan N , Mildenhall A , Gerlic M , Croker BA , D'Cruz AA , Hall C , Kaur Spall S , Anderton H , Masters SL *et al* (2015) RIPK3 promotes cell death and NLRP3 inflammasome activation in the absence of MLKL. Nat Commun 6: 6282 25693118 10.1038/ncomms7282 PMC4346630

[embr202356865-bib-0043] Lawlor KE , Feltham R , Yabal M , Conos SA , Chen KW , Ziehe S , Grass C , Zhan Y , Nguyen TA , Hall C *et al* (2017) XIAP loss triggers RIPK3‐ and caspase‐8‐driven IL‐1beta activation and cell death as a consequence of TLR‐MyD88‐induced cIAP1‐TRAF2 degradation. Cell Rep 20: 668–682 28723569 10.1016/j.celrep.2017.06.073

[embr202356865-bib-0044] Lee EF , Fairlie WD (2021) Discovery, development and application of drugs targeting BCL‐2 pro‐survival proteins in cancer. Biochem Soc Trans 49: 2381–2395 34515749 10.1042/BST20210749 PMC8589430

[embr202356865-bib-0045] Legarda D , Justus SJ , Ang RL , Rikhi N , Li W , Moran TM , Zhang J , Mizoguchi E , Zelic M , Kelliher MA *et al* (2016) CYLD proteolysis protects macrophages from TNF‐mediated auto‐necroptosis induced by LPS and licensed by type I IFN. Cell Rep 15: 2449–2461 27264187 10.1016/j.celrep.2016.05.032 PMC4909532

[embr202356865-bib-0046] Li X , Dou J , You Q , Jiang Z (2021) Inhibitors of BCL2A1/Bfl‐1 protein: potential stock in cancer therapy. Eur J Med Chem 220: 113539 34034128 10.1016/j.ejmech.2021.113539

[embr202356865-bib-0047] Liao Y , Smyth GK , Shi W (2014) featureCounts: an efficient general purpose program for assigning sequence reads to genomic features. Bioinformatics 30: 923–930 24227677 10.1093/bioinformatics/btt656

[embr202356865-bib-0048] Liao Y , Smyth GK , Shi W (2019) The R package Rsubread is easier, faster, cheaper and better for alignment and quantification of RNA sequencing reads. Nucleic Acids Res 47: e47 30783653 10.1093/nar/gkz114 PMC6486549

[embr202356865-bib-0049] Maelfait J , Vercammen E , Janssens S , Schotte P , Haegman M , Magez S , Beyaert R (2008) Stimulation of Toll‐like receptor 3 and 4 induces interleukin‐1beta maturation by caspase‐8. J Exp Med 205: 1967–1973 18725521 10.1084/jem.20071632 PMC2526192

[embr202356865-bib-0050] Malireddi RKS , Gurung P , Mavuluri J , Dasari TK , Klco JM , Chi H , Kanneganti TD (2018) TAK1 restricts spontaneous NLRP3 activation and cell death to control myeloid proliferation. J Exp Med 215: 1023–1034 29500178 10.1084/jem.20171922 PMC5881469

[embr202356865-bib-0051] Malireddi RKS , Gurung P , Kesavardhana S , Samir P , Burton A , Mummareddy H , Vogel P , Pelletier S , Burgula S , Kanneganti TD (2020) Innate immune priming in the absence of TAK1 drives RIPK1 kinase activity‐independent pyroptosis, apoptosis, necroptosis, and inflammatory disease. J Exp Med 217: jem.20191644 31869420 10.1084/jem.20191644 PMC7062518

[embr202356865-bib-0052] Martin M (2011) Cutadapt removes adapter sequences from high‐throughput sequencing reads. EMBnet J 17: 10–12

[embr202356865-bib-0053] Naderer T , Fulcher MC (2018) Targeting apoptosis pathways in infections. J Leukoc Biol 103: 275–285 29372933 10.1189/JLB.4MR0717-286R

[embr202356865-bib-0054] Newton K , Dugger DL , Wickliffe KE , Kapoor N (2014) Activity of protein kinase RIPK3 determines whether cells die by necroptosis or apoptosis. Science 343: 1357–1360 24557836 10.1126/science.1249361

[embr202356865-bib-0055] Newton K , Wickliffe KE , Dugger DL , Maltzman A , Roose‐Girma M , Dohse M , Kőműves L , Webster JD , Dixit VM (2019a) Cleavage of RIPK1 by caspase‐8 is crucial for limiting apoptosis and necroptosis. Nature 574: 428–431 31511692 10.1038/s41586-019-1548-x

[embr202356865-bib-0056] Newton K , Wickliffe KE , Maltzman A , Dugger DL , Reja R , Zhang Y , Roose‐Girma M , Modrusan Z , Sagolla MS , Webster JD *et al* (2019b) Activity of caspase‐8 determines plasticity between cell death pathways. Nature 575: 679–682 31723262 10.1038/s41586-019-1752-8

[embr202356865-bib-0057] Oberst A , Dillon CP , Weinlich R , McCormick LL , Fitzgerald P , Pop C , Hakem R , Salvesen GS , Green DR (2011) Catalytic activity of the caspase‐8‐FLIP_L_ complex inhibits RIPK3‐dependent necrosis. Nature 471: 363–367 21368763 10.1038/nature09852 PMC3077893

[embr202356865-bib-0058] O'Donnell MA , Perez‐Jimenez E , Oberst A , Ng A , Massoumi R , Xavier R , Green DR , Ting AT (2011) Caspase 8 inhibits programmed necrosis by processing CYLD. Nat Cell Biol 13: 1437–1442 22037414 10.1038/ncb2362 PMC3229661

[embr202356865-bib-0059] Ohmer M , Weber A , Sutter G , Ehrhardt K , Zimmermann A , Hacker G (2016) Anti‐apoptotic Bcl‐XL but not Mcl‐1 contributes to protection against virus‐induced apoptosis. Cell Death Dis 7: e2340 27537523 10.1038/cddis.2016.242 PMC5108327

[embr202356865-bib-0060] Oltersdorf T , Elmore SW , Shoemaker AR , Armstrong RC , Augeri DJ , Belli BA , Bruncko M , Deckwerth TL , Dinges J , Hajduk PJ *et al* (2005) An inhibitor of Bcl‐2 family proteins induces regression of solid tumours. Nature 435: 677–681 15902208 10.1038/nature03579

[embr202356865-bib-0061] Orning P , Weng D , Starheim K , Ratner D , Best Z , Lee B , Brooks A , Xia S , Wu H , Kelliher MA *et al* (2018) Pathogen blockade of TAK1 triggers caspase‐8‐dependent cleavage of gasdermin D and cell death. Science 362: 1064–1069 30361383 10.1126/science.aau2818 PMC6522129

[embr202356865-bib-0062] Orzalli MH , Prochera A , Payne L , Smith A , Garlick JA , Kagan JC (2021) Virus‐mediated inactivation of anti‐apoptotic Bcl‐2 family members promotes Gasdermin‐E‐dependent pyroptosis in barrier epithelial cells. Immunity 54: 1447–1462 33979579 10.1016/j.immuni.2021.04.012 PMC8594743

[embr202356865-bib-0063] Pathirana RD , Kaparakis‐Liaskos M (2016) Bacterial membrane vesicles: biogenesis, immune regulation and pathogenesis. Cell Microbiol 18: 1518–1524 27564529 10.1111/cmi.12658

[embr202356865-bib-0064] Philip NH , DeLaney A , Peterson LW , Santos‐Marrero M , Grier JT , Sun Y , Wynosky‐Dolfi MA , Zwack EE , Hu B , Olsen TM *et al* (2016) Activity of uncleaved caspase‐8 controls anti‐bacterial immune defense and TLR‐induced cytokine production independent of cell death. PLoS Pathog 12: e1005910 27737018 10.1371/journal.ppat.1005910 PMC5063320

[embr202356865-bib-0065] Rijal D , Ariana A , Wight A , Kim K , Alturki NA , Aamir Z , Ametepe ES , Korneluk RG , Tiedje C , Menon MB *et al* (2018) Differentiated macrophages acquire a pro‐inflammatory and cell death‐resistant phenotype due to increasing XIAP and p38‐mediated inhibition of RipK1. J Biol Chem 293: 11913–11927 29899110 10.1074/jbc.RA118.003614 PMC6066316

[embr202356865-bib-0066] Riley JS , Tait SW (2020) Mitochondrial DNA in inflammation and immunity. EMBO Rep 21: e49799 32202065 10.15252/embr.201949799 PMC7132203

[embr202356865-bib-0067] Rinkenberger JL , Horning S , Klocke B , Roth K , Korsmeyer SJ (2000) Mcl‐1 deficiency results in peri‐implantation embryonic lethality. Genes Dev 14: 23–27 10640272 PMC316347

[embr202356865-bib-0068] Robinson MD , Oshlack A (2010) A scaling normalization method for differential expression analysis of RNA‐seq data. Genome Biol 11: R25 20196867 10.1186/gb-2010-11-3-r25 PMC2864565

[embr202356865-bib-0069] Rohner L , Reinhart R , Iype J , Bachmann S , Kaufmann T , Fux M (2020) Impact of BH3‐mimetics on human and mouse blood leukocytes: a comparative study. Sci Rep 10: 222 31937836 10.1038/s41598-019-57000-x PMC6959258

[embr202356865-bib-0070] Ruhl S , Broz P (2015) Caspase‐11 activates a canonical NLRP3 inflammasome by promoting K^+^ efflux. Eur J Immunol 45: 2927–2936 26173909 10.1002/eji.201545772

[embr202356865-bib-0071] Sagulenko V , Thygesen SJ , Sester DP , Idris A , Cridland JA , Vajjhala PR , Roberts TL , Schroder K , Vince JE , Hill JM *et al* (2013) AIM2 and NLRP3 inflammasomes activate both apoptotic and pyroptotic death pathways via ASC. Cell Death Differ 20: 1149–1160 23645208 10.1038/cdd.2013.37 PMC3741496

[embr202356865-bib-0072] Sarhan J , Liu BC , Muendlein HI , Li P , Nilson R , Tang AY , Rongvaux A , Bunnell SC , Shao F , Green DR *et al* (2018) Caspase‐8 induces cleavage of gasdermin D to elicit pyroptosis during *Yersinia* infection. Proc Natl Acad Sci USA 115: E10888–E10897 30381458 10.1073/pnas.1809548115 PMC6243247

[embr202356865-bib-0073] Schenk RL , Tuzlak S , Carrington EM , Zhan Y , Heinzel S , Teh CE , Gray DH , Tai L , Lew AM , Villunger A *et al* (2017) Characterisation of mice lacking all functional isoforms of the pro‐survival BCL‐2 family member A1 reveals minor defects in the haematopoietic compartment. Cell Death Differ 24: 534–545 28085150 10.1038/cdd.2016.156 PMC5344213

[embr202356865-bib-0074] Schenk RL , Gangoda L , Lawlor KE , O'Reilly LA , Strasser A , Herold MJ (2020) The pro‐survival Bcl‐2 family member A1 delays spontaneous and FAS ligand‐induced apoptosis of activated neutrophils. Cell Death Dis 11: 474 32555150 10.1038/s41419-020-2676-9 PMC7303176

[embr202356865-bib-0075] Shi J , Zhao Y , Wang K , Shi X , Wang Y , Huang H , Zhuang Y , Cai T , Wang F , Shao F (2015) Cleavage of GSDMD by inflammatory caspases determines pyroptotic cell death. Nature 526: 660–665 26375003 10.1038/nature15514

[embr202356865-bib-0076] Shimada K , Crother TR , Karlin J , Dagvadorj J , Chiba N , Chen S , Ramanujan VK , Wolf AJ , Vergnes L , Ojcius DM *et al* (2012) Oxidized mitochondrial DNA activates the NLRP3 inflammasome during apoptosis. Immunity 36: 401–414 22342844 10.1016/j.immuni.2012.01.009 PMC3312986

[embr202356865-bib-0077] Simpson DS , Pang J , Weir A , Kong IY , Fritsch M , Rashidi M , Cooney JP , Davidson KC , Speir M , Djajawi TM *et al* (2022) Interferon‐γ primes macrophages for pathogen ligand‐induced killing via a caspase‐8 and mitochondrial cell death pathway. Immunity 55: 423–441 35139355 10.1016/j.immuni.2022.01.003 PMC8822620

[embr202356865-bib-0078] Singh R , Letai A , Sarosiek K (2019) Regulation of apoptosis in health and disease: the balancing act of BCL‐2 family proteins. Nat Rev Mol Cell Biol 20: 175–193 30655609 10.1038/s41580-018-0089-8 PMC7325303

[embr202356865-bib-0079] Souers AJ , Leverson JD , Boghaert ER , Ackler SL , Catron ND , Chen J , Dayton BD , Ding H , Enschede SH , Fairbrother WJ *et al* (2013) ABT‐199, a potent and selective BCL‐2 inhibitor, achieves antitumor activity while sparing platelets. Nat Med 19: 202–208 23291630 10.1038/nm.3048

[embr202356865-bib-0080] Speir M , Lawlor KE (2021) RIP‐roaring inflammation: RIPK1 and RIPK3 driven NLRP3 inflammasome activation and autoinflammatory disease. Semin Cell Dev Biol 109: 114–124 32771377 10.1016/j.semcdb.2020.07.011

[embr202356865-bib-0081] Speir M , Lawlor KE , Glaser SP , Abraham G , Chow S , Vogrin A , Schulze KE , Schuelein R , O'Reilly LA , Mason K *et al* (2016) Eliminating *Legionella* by inhibiting BCL‐XL to induce macrophage apoptosis. Nat Microbiol 1: 15034 27572165 10.1038/nmicrobiol.2015.34

[embr202356865-bib-0082] Steimer DA , Boyd K , Takeuchi O , Fisher JK , Zambetti GP , Opferman JT (2009) Selective roles for antiapoptotic MCL‐1 during granulocyte development and macrophage effector function. Blood 113: 2805–2815 19064728 10.1182/blood-2008-05-159145 PMC2661864

[embr202356865-bib-0083] Suzuki T , Okamoto T , Katoh H , Sugiyama Y , Kusakabe S , Tokunaga M , Hirano J , Miyata Y , Fukuhara T , Ikawa M *et al* (2018) Infection with flaviviruses requires BCLXL for cell survival. PLoS Pathog 14: e1007299 30261081 10.1371/journal.ppat.1007299 PMC6177207

[embr202356865-bib-0084] Taabazuing CY , Okondo MC , Bachovchin DA (2017) Pyroptosis and apoptosis pathways engage in bidirectional crosstalk in monocytes and macrophages. Cell Chem Biol 24: 507–514 28392147 10.1016/j.chembiol.2017.03.009 PMC5467448

[embr202356865-bib-0085] Tse C , Shoemaker AR , Adickes J , Anderson MG , Chen J , Jin S , Johnson EF , Marsh KC , Mitten MJ , Nimmer P *et al* (2008) ABT‐263: a potent and orally bioavailable Bcl‐2 family inhibitor. Cancer Res 68: 3421–3428 18451170 10.1158/0008-5472.CAN-07-5836

[embr202356865-bib-0086] Tummers B , Green DR (2017) Caspase‐8: regulating life and death. Immunol Rev 277: 76–89 28462525 10.1111/imr.12541 PMC5417704

[embr202356865-bib-0087] van Delft MF , Wei AH , Mason KD , Vandenberg CJ , Chen L , Czabotar PE , Willis SN , Scott CL , Day CL , Cory S *et al* (2006) The BH3 mimetic ABT‐737 targets selective Bcl‐2 proteins and efficiently induces apoptosis via Bak/Bax if Mcl‐1 is neutralized. Cancer Cell 10: 389–399 17097561 10.1016/j.ccr.2006.08.027 PMC2953559

[embr202356865-bib-0088] Vier J , Groth M , Sochalska M , Kirschnek S (2016) The anti‐apoptotic Bcl‐2 family protein A1/Bfl‐1 regulates neutrophil survival and homeostasis and is controlled via PI3K and JAK/STAT signaling. Cell Death Dis 7: e2103 26890142 10.1038/cddis.2016.23 PMC5399193

[embr202356865-bib-0089] Vijayaraj SL , Feltham R , Rashidi M , Frank D , Liu Z , Simpson DS , Ebert G , Vince A , Herold MJ , Kueh A *et al* (2021) The ubiquitylation of IL‐1beta limits its cleavage by caspase‐1 and targets it for proteasomal degradation. Nat Commun 12: 2713 33976225 10.1038/s41467-021-22979-3 PMC8113568

[embr202356865-bib-0090] Vince JE , Wong WW , Gentle I , Lawlor KE , Allam R , O'Reilly L , Mason K , Gross O , Ma S , Guarda G *et al* (2012) Inhibitor of apoptosis proteins limit RIP3 kinase‐dependent interleukin‐1 activation. Immunity 36: 215–227 22365665 10.1016/j.immuni.2012.01.012

[embr202356865-bib-0091] Vince JE , De Nardo D , Gao W , Vince AJ , Hall C , McArthur K , Simpson D , Vijayaraj S , Lindqvist LM , Bouillet P *et al* (2018) The mitochondrial apoptotic effectors BAX/BAK activate caspase‐3 and ‐7 to trigger NLRP3 inflammasome and caspase‐8 driven IL‐1beta activation. Cell Rep 25: 2339–2353 30485804 10.1016/j.celrep.2018.10.103

[embr202356865-bib-0092] Wang L , Du F , Wang X (2008) TNF‐alpha induces two distinct caspase‐8 activation pathways. Cell 133: 693–703 18485876 10.1016/j.cell.2008.03.036

[embr202356865-bib-0093] Waterhouse NJ , Trapani JA (2003) A new quantitative assay for cytochrome c release in apoptotic cells. Cell Death Differ 10: 853–855 12815469 10.1038/sj.cdd.4401263

[embr202356865-bib-0094] Wong WW , Vince JE , Lalaoui N , Lawlor KE , Chau D , Bankovacki A , Anderton H , Metcalf D , O'Reilly L , Jost PJ *et al* (2014) cIAPs and XIAP regulate myelopoiesis through cytokine production in an RIPK1‐ and RIPK3‐dependent manner. Blood 123: 2562–2572 24497535 10.1182/blood-2013-06-510743

[embr202356865-bib-0095] Xian H , Watari K , Sanchez‐Lopez E , Offenberger J , Onyuru J , Sampath H , Ying W , Hoffman HM , Shadel GS , Karin M (2022) Oxidized DNA fragments exit mitochondria via mPTP‐ and VDAC‐dependent channels to activate NLRP3 inflammasome and interferon signaling. Immunity 55: 1370–1385 35835107 10.1016/j.immuni.2022.06.007 PMC9378606

[embr202356865-bib-0096] Yabal M , Muller N , Adler H , Knies N , Gross CJ , Damgaard RB , Kanegane H , Ringelhan M , Kaufmann T , Heikenwalder M *et al* (2014) XIAP restricts TNF‐ and RIP3‐dependent cell death and inflammasome activation. Cell Rep 7: 1796–1808 24882010 10.1016/j.celrep.2014.05.008

[embr202356865-bib-0097] Yabal M , Calleja DJ , Simpson DS , Lawlor KE (2019) Stressing out the mitochondria: mechanistic insights into NLRP3 inflammasome activation. J Leukoc Biol 105: 377–399 30589456 10.1002/JLB.MR0318-124R

[embr202356865-bib-0098] Yona S , Kim KW , Wolf Y , Mildner A , Varol D , Breker M , Strauss‐Ayali D , Viukov S , Guilliams M , Misharin A *et al* (2013) Fate mapping reveals origins and dynamics of monocytes and tissue macrophages under homeostasis. Immunity 38: 79–91 23273845 10.1016/j.immuni.2012.12.001 PMC3908543

