# Author Correction: A1 is induced by pathogen ligands to limit myeloid cell death and NLRP3 inflammasome activation

**DOI:** 10.1038/s44319-025-00609-z

**Published:** 2025-12-22

**Authors:** Mary Speir, Hazel Tye, Timothy A Gottschalk, Daniel S Simpson, Tirta M Djajawi, Pankaj Deo, Rebecca L Ambrose, Stephanie A Conos, Jack Emery, Gilu Abraham, Ashlyn Pascoe, Sebastian A Hughes, Ashley Weir, Edwin D Hawkins, Isabella Kong, Marco J Herold, Jaclyn S Pearson, Najoua Lalaoui, Thomas Naderer, James E Vince, Kate E Lawlor

**Affiliations:** 1 Centre for Innate Immunity and Infectious Diseases, Hudson Institute of Medical Research, Clayton, VIC Australia; 2 Department of Molecular and Translational Science, Monash University, Clayton, VIC Australia; 3 The Walter and Eliza Hall Institute of Medical Research, Parkville, VIC Australia; 4 Department of Medical Biology, University of Melbourne, Parkville, VIC Australia; 5 Department of Biochemistry and Molecular Biology, Monash Biomedicine Discovery Institute, Monash University, Clayton, VIC Australia; 6 Department of Microbiology, Monash University, Clayton, VIC Australia

## Abstract

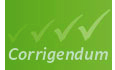

**Correction to:** *EMBO Reports* (2023) 24:e56865. 10.15252/embr.202356865 | Published online 17 October 2023

The authors contacted the journal after identifying an error in Figure 5F of the published manuscript.

**Figure 5F is withdrawn and replaced**.

**Figure 5F:**
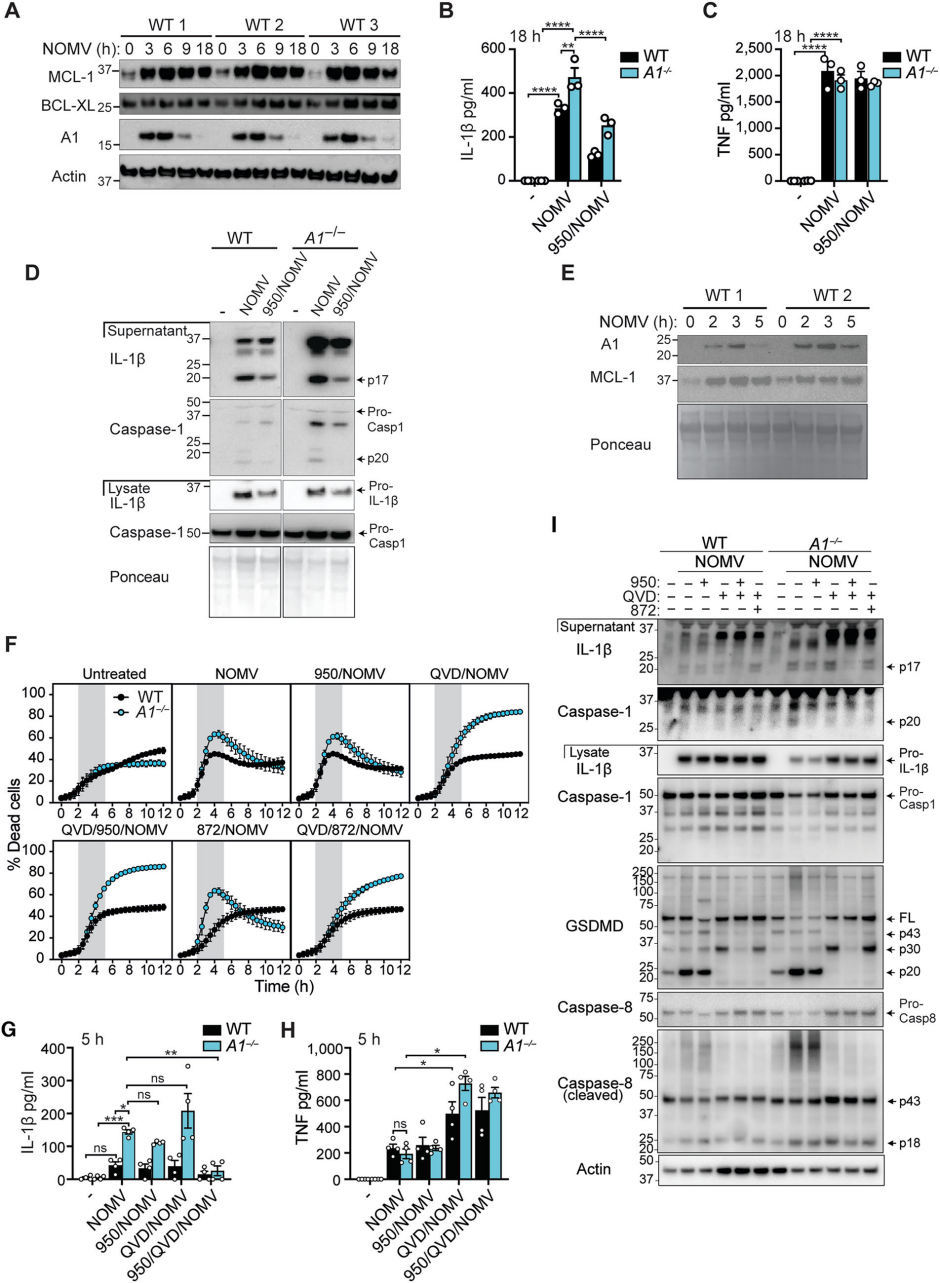
Original.

**Figure 5F:**
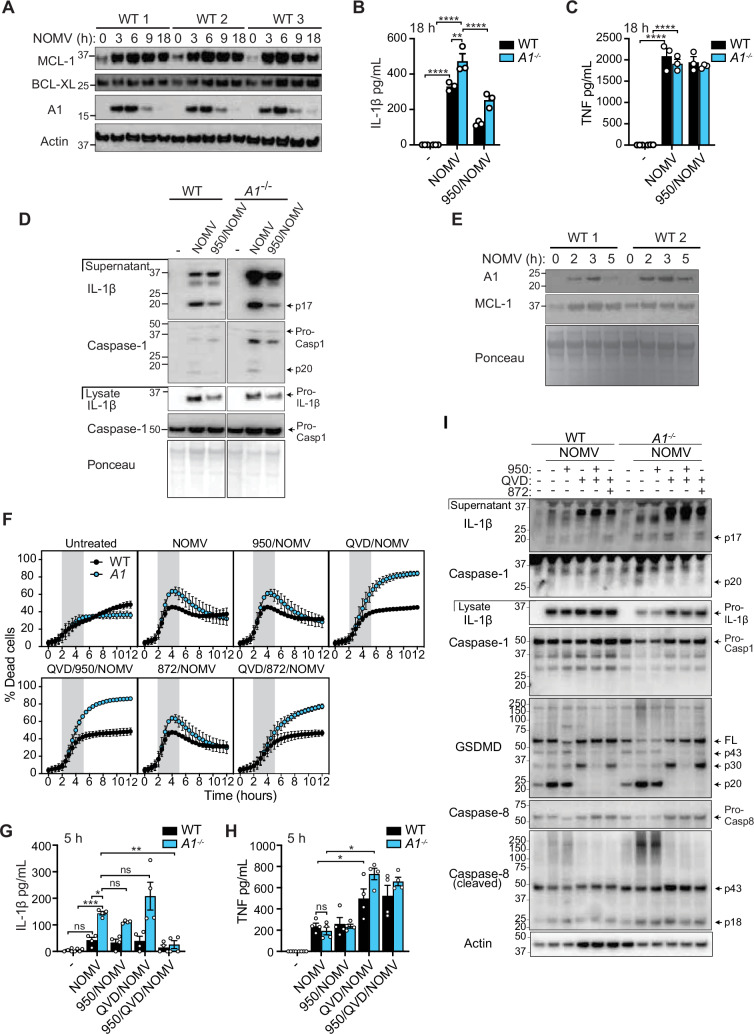
Corrected.

Author Statement:

During the revision process, when separating the different stimuli to make the death curves easier to follow, an error occurred in Figure 5F. The WT QVD/872/NOMV panel was mistakenly duplicated in place of the WT 872/NOMV panel and data was presented as SEM rather than SD. Figure 5F is now corrected. We have confirmed that the original source data associated with this figure are correct and were not affected by this error.

This error does not affect the text, interpretation, or overall conclusions of the manuscript, as the results were analysed and discussed based on the correct dataset.

All authors agree to this author correction.

