# Supplementary material for: A1 is induced by pathogen ligands to limit myeloid cell death and NLRP3 inflammasome activation
Source: EMBO Rep. 2023 Oct 17;24(11):e56865. doi: 10.15252/embr.202356865 (PMC10626451; doi:10.15252/embr.202356865)
Supplement: Supplementary file 1 — Appendix [file EMBR-24-e56865-s002.pdf]

Appendix Data

A1 is induced by pathogen ligands to limit myeloid cell death and NLRP3 inflammasome activation

Table of contents

Appendix Figure S1. A1 deficiency leads to the rapid loss of cytochrome-*c* in LPS-primed macrophages upon BCL-XL and MCL-1 targeting……………………………………………….2

Appendix Figure S2. LPS-primed A1-deficient Ly6C^{hi} BMMo display accelerated cytochrome-*c* loss upon MCL-1 targeting………………………………………………………………………..3

Appendix Figure S3. Gating strategy for sorting primary Ly6C^{hi} inflammatory monocytes from the bone marrow. ………………………………………………………………………….………4

Appendix Figure S4. *Neisseria Gonorrhoeae*-derived OMVs trigger rapid loss of cytochrome-*c* in primary bone marrow Ly6C^{hi} monocytes lacking A1…………….……………………...….5

Appendix Figure S5. A1 deficiency does not perturb peripheral blood immune cell responses upon intraperitoneal NOMV injection.….. ………………………..………………………..…6

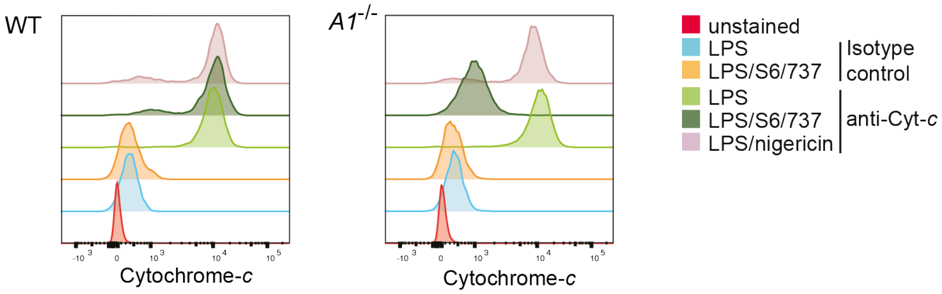

Appendix Figure S1. A1 deficiency leads to the rapid loss of cytochrome-*c* in LPS-primed macrophages upon BCL-XL and MCL-1 targeting. WT and A1-deficient (*A1*$^{-/-}$) BMDMs were primed with B5 LPS (50 ng/ml) for 3 h, prior to the addition of S63845 (S6; 10 µM) and ABT-737 (737; 500 nM) for 2-3 h. Alternatively, LPS-primed cells were treated with Nigericin (10 µM) for 1 h. MOMP-dependent loss of cytochrome-*c* staining was analysed by flow cytometry. Data are representative of two biological experiments.

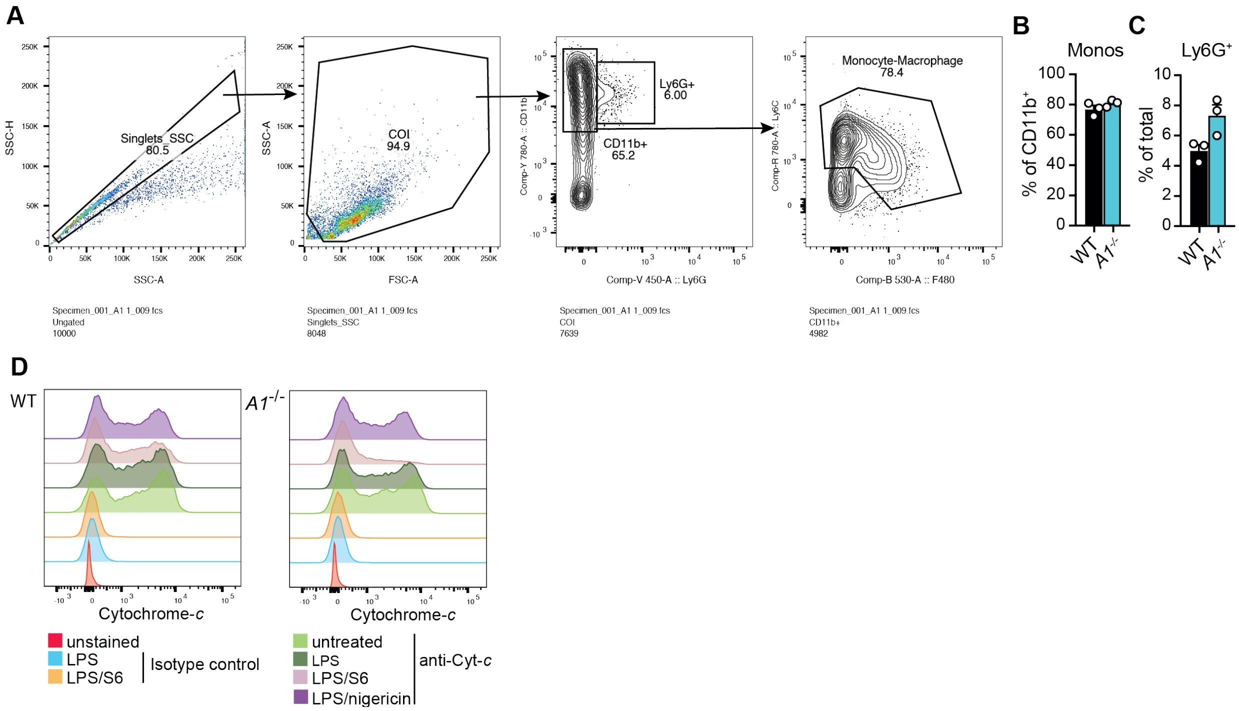

Appendix Figure S2. LPS-primed A1-deficient Ly6C^{hi} BMMo display accelerated cytochrome-*c* loss upon MCL-1 targeting

A-C Purity of WT and A1-deficient (*A1*$^{-/-}$) BMMo cultures after 4 days of LCCM culture was analysed by flow cytometry. **(A)** Gating strategy: Doublets and cell debris were removed and monocytes/immature macrophage-like cells were analysed as CD11b^{+}Ly6C^{+} (and CD11b^{+}Ly6C^{int}F4/80$^{lo\text{-}int}$) cells. **(B)** % of CD11b^{+} cells that were monocyte/immature macrophage-like cells. **(C)** % of total cells that were Ly6G^{+} neutrophils.

D WT and A1-deficient (*A1*$^{-/-}$) BMMo were primed with B5 LPS (50 ng/ml) for 3 h and treated with S63845 (S6; 10 µM) or nigericin (10 µM) for 2 h or 1 h, respectively. MOMP was evaluated by flow cytometric analysis of loss of cytochrome-*c* retention from monocyte/immature macrophage-like cells.

Data information: Each dot represents an individual biological replicate (B, C). Data are representative of at least two independent biological experiments and presented (B, C) as the mean + SEM.

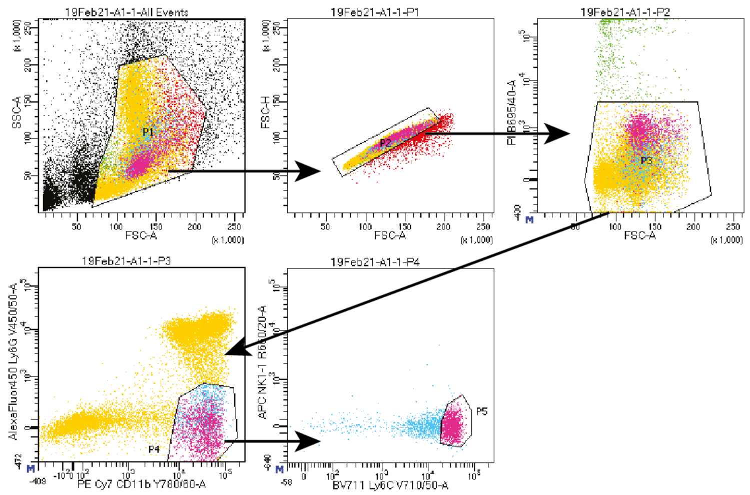

Tube: A1-1

Population	#Events	%Parent	%Total
All Events	34,148	####	100.0
P1	22,378	65.5	65.5
P2	20,631	92.2	60.4
P3	20,000	96.9	58.6
P4	2,950	14.8	8.6
P5	1,811	61.4	5.3

19Feb21
Sample A1.1

P5: 949K

Appendix Figure S3. Gating strategy for sorting primary Ly6C^{hi} inflammatory monocytes from the bone marrow. Bone marrow was isolated from WT and A1-deficient mice and stained with fluorescently labelled anti-mouse mAb against CD11b, Ly6G, NK1.1 and Ly6C. Shown is the flow cytometry gating strategy used to isolate Ly6C^{hi} monocytes from the bone marrow.

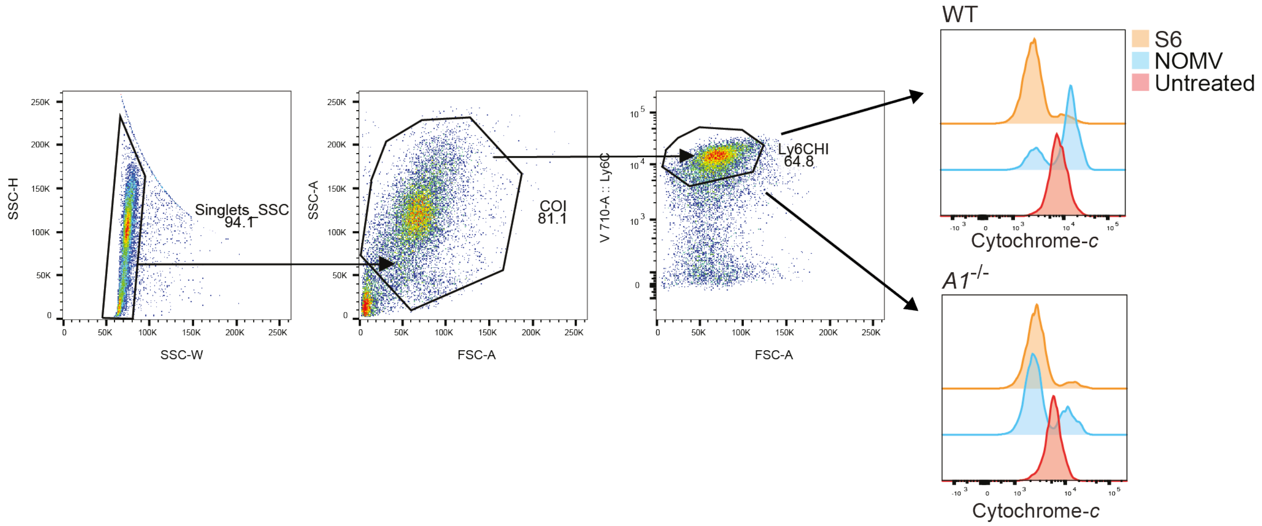

Appendix Figure S4. ***Neisseria Gonorrhoeae*****-derived OMVs trigger rapid loss of cytochrome-*c* in primary bone marrow Ly6C^{hi} monocytes lacking A1**

Monocytes were isolated from WT and A1-deficient (*A1*$^{-/-}$) bone marrow using magnetic separation and stimulated with NOMVs (50 µg/ml) or S63845 (S6; 10 µM) for 3 h. Flow cytometry gating strategy showing the proportion of Ly6C^{hi} cell that were then analysed for MOMP-induced loss of cytochrome-*c* staining.

Data information: Data are representative of at least two independent biological experiments.

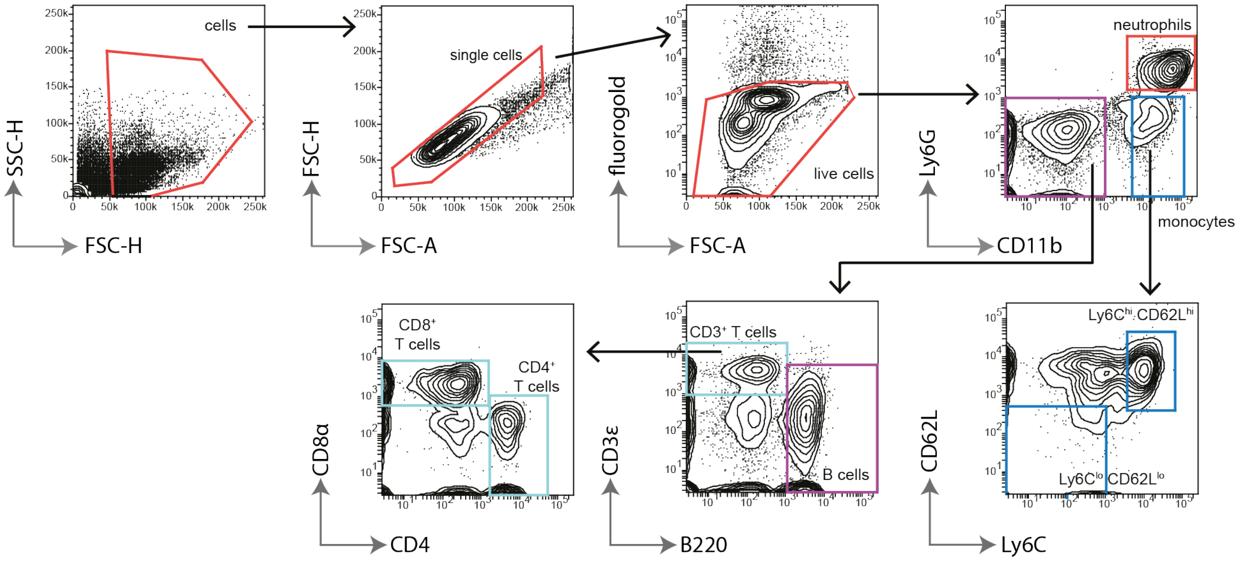

Appendix Figure S5. A1 deficiency does not perturb peripheral blood immune cell responses upon intraperitoneal NOMV injection. WT and A1-deficient mice were injected intraperitoneally with 100 μg of NOMVs (or PBS) and peripheral blood and peritoneal lavage fluid harvested after 6 h. Flow cytometry gating strategy for identifying immune cell populations in peripheral blood of PBS or NOMV treated mice.
