# Supplementary material for: A1 is induced by pathogen ligands to limit myeloid cell death and NLRP3 inflammasome activation
Source: EMBO Rep. 2023 Oct 17;24(11):e56865. doi: 10.15252/embr.202356865 (PMC10626451; doi:10.15252/embr.202356865)
Supplement: Supplementary file 2 — Expanded View Figures PDF [file EMBR-24-e56865-s007.pdf]

Expanded View Figures

Figure EV1. LPS priming delays cell death upon BCL-XL and MCL-1 targeting in macrophages.

A–H WT BMDMs were primed, as specified, with B5 LPS (50 ng/ml) for 3 h before addition of S63845 (S6; 10 μM), ABT-737 (737; 500 nM), A-1331852 (852; 1 μM), and/or cycloheximide (CHX; 20 μg/ml) for 6 or 24 h, as indicated. (A, B) Cell death was quantified by flow cytometric analysis of PI uptake. (C, D) IL-1β and (E–H) TNF levels were measured in the cell supernatants by ELISA.

I, J WT BMDMs were treated with B4 LPS for 7 h before RNA isolation and 3′ mRNA sequencing. (I) Multi-dimensional scaling (MDS) plot of the top 1,000 differentially expressed genes (DEGs) between untreated and LPS-treated BMDMs. (J) Gene Ontology (GO) analysis showing the top 25 pathways of significant DEGs upregulated in LPS-treated versus untreated BMDMs. Adjusted $P \leq 0.05$ and cut-off values logFC ≥ 1 or logFC ≤ -1.

K WT and A1-deficient ($A1^{-/-}$) BMDMs were primed, as specified, with B5 LPS (50 ng/ml) and cell lysates analysed by immunoblot for up to 24 h for the indicated proteins.

L WT BMDMs were primed with B5 LPS (50 ng/ml) for 2–3 h and pre-treated with MG132 (5 μM), Bafilomycin A1 (BafA1; 100 nM) and/or Q-VD-OPh (QVD; 40 μM), as indicated, prior to treatment with and without CHX (20 μg/ml) for the indicated times. Cell lysates were interrogated for the indicated proteins by immunoblot.

Data information: Each dot represents an individual biological replicate. (A–H, K, L) Data are representative of at least two independent biological experiments and presented as the mean + SEM. ns, not significant, ****$P < 0.0001$ (one-way ANOVA with Tukey's multiple comparisons test). (K, L) Ponceau stains were used as a control for the loading of total protein and β-actin used as an additional loading control.
Source data are available online for this figure.

 © 2023 The Authors

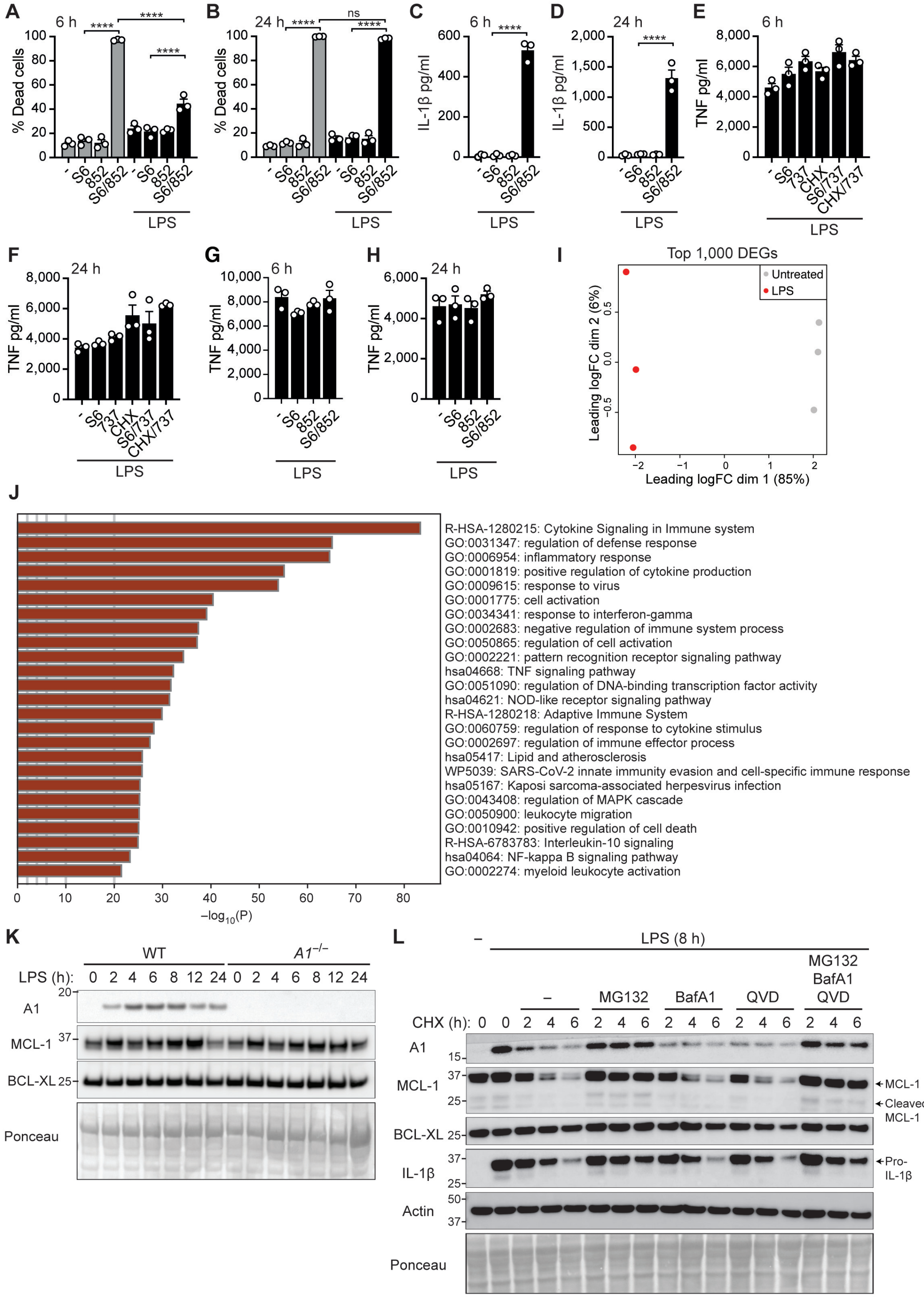
A
6 h
% Dead cells
S6
852
S6/852
LPS
B
24 h
ns
C
IL-1β pg/ml
D
E
TNF pg/ml
737
CHX
S6/737
CHX/737
F
G
H
I
Top 1,000 DEGs
Untreated
LPS
Leading logFC dim 2 (6%)
Leading logFC dim 1 (85%)
J
R-HSA-1280215: Cytokine Signaling in Immune system
GO:0031347: regulation of defense response
GO:0006954: inflammatory response
GO:0001819: positive regulation of cytokine production
GO:0009615: response to virus
GO:0001775: cell activation
GO:0034341: response to interferon-gamma
GO:0002683: negative regulation of immune system process
GO:0050865: regulation of cell activation
GO:0002221: pattern recognition receptor signaling pathway
hsa04668: TNF signaling pathway
GO:0051090: regulation of DNA-binding transcription factor activity
hsa04621: NOD-like receptor signaling pathway
R-HSA-1280218: Adaptive Immune System
GO:0060759: regulation of response to cytokine stimulus
GO:0002697: regulation of immune effector process
hsa05417: Lipid and atherosclerosis
WP5039: SARS-CoV-2 innate immunity evasion and cell-specific immune response
hsa05167: Kaposi sarcoma-associated herpesvirus infection
GO:0043408: regulation of MAPK cascade
GO:0050900: leukocyte migration
GO:0010942: positive regulation of cell death
R-HSA-6783783: Interleukin-10 signaling
hsa04064: NF-kappa B signaling pathway
GO:0002274: myeloid leukocyte activation
−log10(P)
K
WT
A1−/−
LPS (h): 0 2 4 6 8 12 24 0 2 4 6 8 12 24
A1
MCL-1
BCL-XL
Ponceau
L
LPS (8 h)
MG132
BafA1
QVD
CHX (h): 0 0 2 4 6 2 4 6 2 4 6 2 4 6 2 4 6
A1
MCL-1
Cleaved MCL-1
BCL-XL
IL-1β
Pro-IL-1β
Actin
Ponceau

Figure EV1.

Figure EV2. A1 limits intrinsic apoptosis and downstream IL-1β activation in LPS-primed macrophages upon BCL-XL and MCL-1 targeting.

A–I WT and A1-deficient (*A1*$^{-/-}$) BMDMs were primed with B5 LPS (50 ng/ml) for 3 h, pre-treated with the NLRP3 inhibitor MCC950 (950; 5 μM) for the final 20–30 min of priming, as indicated, prior to the addition of S63845 (S6; 10 μM), ABT-737 (737; 500 nM) or ABT-199 (199; 1 μM), as indicated. (A, B) Cell death was measured by PI uptake and flow cytometric analysis after 6 and 24 h. (C, D, H, I) IL-1β and (E, F) TNF levels were measured in culture supernatants after 4–6 and 24 h by ELISA. (G) Cell supernatants and lysates were interrogated by immunoblot for the indicated proteins after 6 h.

J–L WT and A1-deficient (*A1*$^{-/-}$) BMDMs were primed for 3 h with B5 LPS (50 ng/ml) and treated with 10 μM Nigericin for 30 min. (J) Cell death was measured by PI uptake and flow cytometric analysis. (K) IL-1β and (L) TNF levels were measured in the cell supernatants by ELISA.

M, N WT and A1-deficient (*A1*$^{-/-}$) BMDMs were primed for 12 h with Pam_3Cys (500 ng/ml) and then treated with 12.5 μg/ml FuGENE (Fug)-transfected LPS for 24 h. (M) Cell death was measured using an LDH release assay. (N) IL-1β levels were measured in the cell supernatants by ELISA.

O, P Unprimed WT and A1-deficient (*A1*$^{-/-}$) BMDMs were infected with *S.* Typhimurium at MOI 10 for up to 24 h. (O) Cell death was measured using an LDH release assay. (P) IL-1β levels were measured in the cell supernatants by ELISA.

Data information: Each dot represents an individual biological replicate. Data are representative of at least three (A–F, H–L) or two (G, M–P) independent biological experiments and (A–F, H–P) presented as the mean + SEM. ns, not significant, *$P < 0.05$, ***$P < 0.005$, ****$P < 0.0001$ (two-way ANOVA with Tukey's multiple comparisons test). (G) Ponceau stain was used as a control for the loading of total protein.
Source data are available online for this figure.

© 2023 The Authors

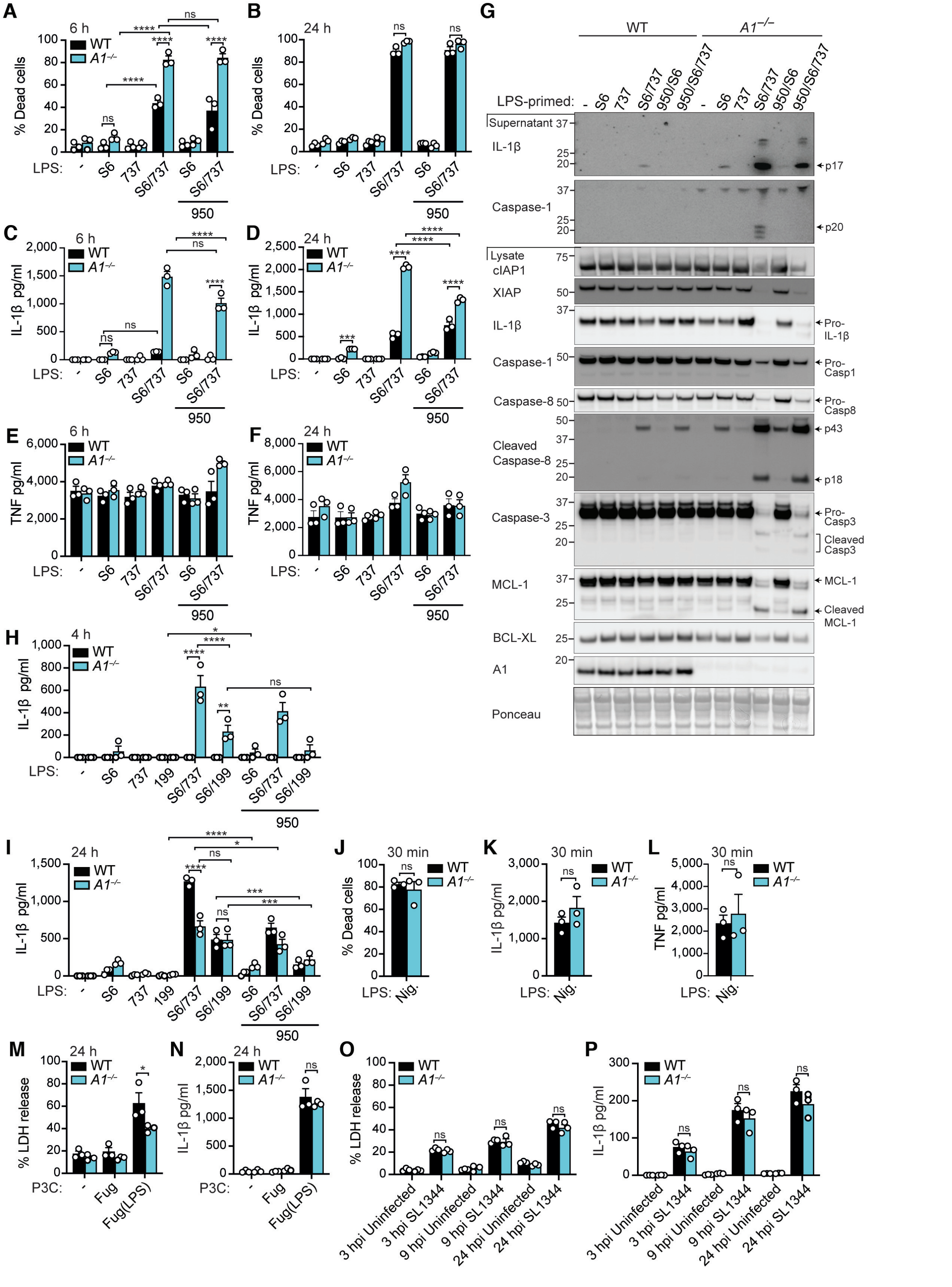

Figure EV2.

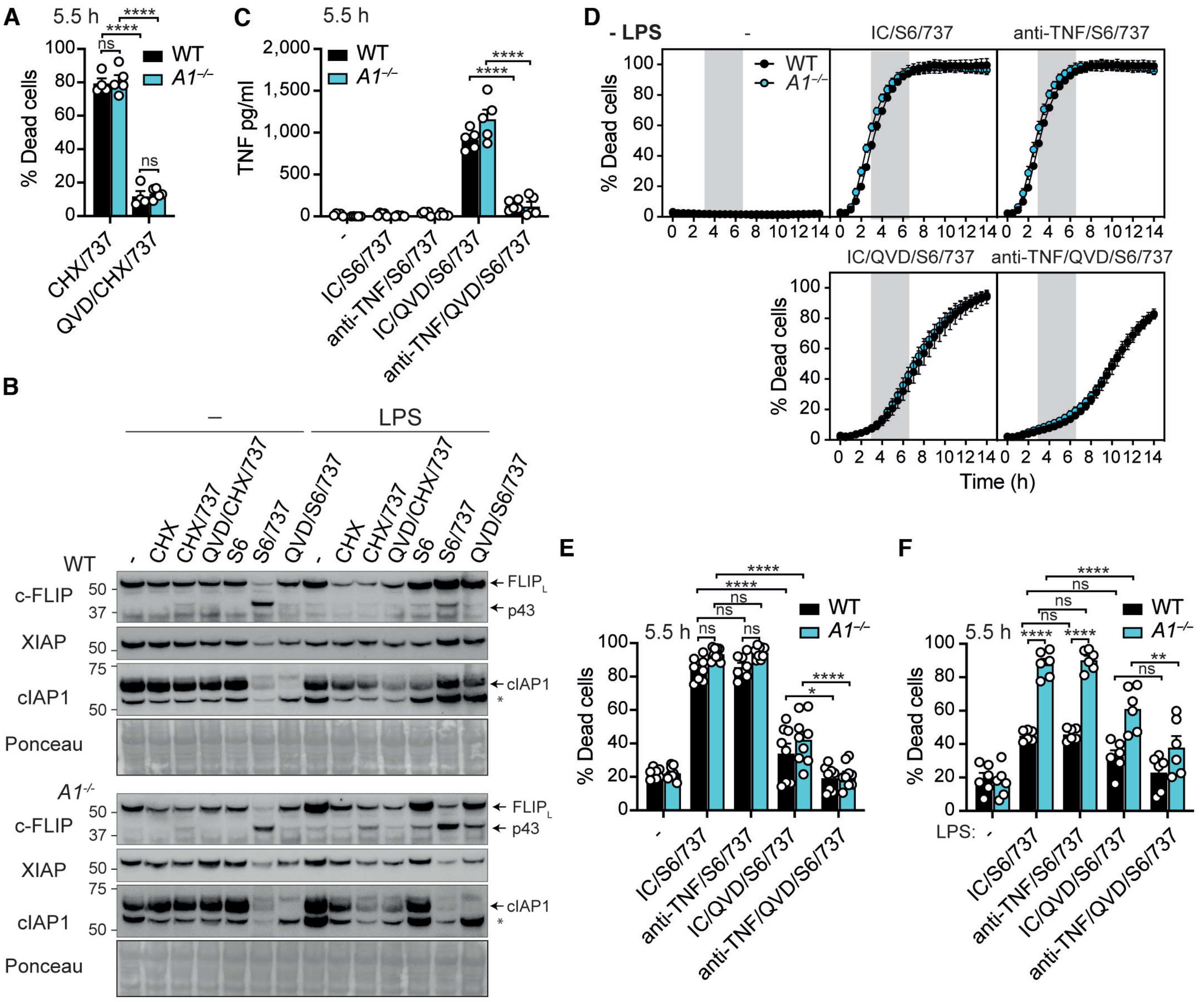

Figure EV3. In the presence of caspase inhibition, specific activation of BAX/BAK using S63845 and ABT-737 triggers TNF-dependent necroptosis.

A, B WT and A1-deficient ($A1^{-/-}$) BMDMs were primed, as indicated, with B5 LPS (50 ng/ml) for 3 h, pre-treated with the pan-caspase inhibitor Q-VD-OPh (QVD; 40 μM) and/or GSK'872 (872; 1 μM) for the final 20–30 min of priming, as indicated, prior to the addition of S63845 (S6; 10 μM), ABT-737 (737; 500 nM), CHX (20 μg/ml), and z-VAD-fmk (zVAD; 50 μM), as specified, for up to 6 h. (A) Cell death was measured via flow cytometric analysis of PI uptake. (B) Cell lysates were analysed by immunoblot for the indicated proteins after 2 h. *cFLIP re-probe.

C–E WT and A1-deficient ($A1^{-/-}$) BMDMs were treated with 20 μg/ml isotype control (IC; GL113) or anti-TNF monoclonal antibody (XT22), as indicated. Cells were next incubated with Q-VD-OPh (QVD; 40 μM), as specified, and treated with S63845 (S6; 10 μM) and ABT-737 (737; 500 nM). (C) TNF levels were measured in the culture supernatants by ELISA. (D) Cell death was measured by PI uptake via time-lapse IncuCyte imaging. (E) Cell death was measured by PI uptake and flow cytometric analysis after 5.5 h.

F WT and A1-deficient ($A1^{-/-}$) BMDMs were treated with 20 μg/ml isotype control (IC; GL113) or anti-TNF monoclonal antibody (XT22), as indicated. Cells were next treated with LPS (50 ng/ml) for 3 h, incubated with Q-VD-OPh (QVD; 40 μM) for the last 30 min of priming, as specified, and treated with S63845 (S6; 10 μM) and ABT-737 (737; 500 nM) for 5.5 h. Cell death was measured by PI uptake and flow cytometric analysis.

Data information: Each dot represents an individual biological replicate. Data are presented as the mean + or ± SEM from 2 (A, C, D, F) or 3 pooled (E) biological experiments. ns, not significant, $^*P < 0.05$, $^{**}P < 0.01$, $^{****}P < 0.0001$ (two-way ANOVA with Tukey's multiple comparisons test). (B) Data are representative of two independent biological experiments, and Ponceau stain was used as a control for the loading of total protein.
Source data are available online for this figure.

 © 2023 The Authors

Figure EV4. A1 deficiency sensitises inflammatory monocytes to NOMV-induced BAX/BAK inflammatory signalling.

A WT BMMo were treated with B5 LPS (50 ng/ml) for up to 18 h before immunoblot analysis of cell lysates for the indicated proteins.

B WT and A1-deficient ($A1^{-/-}$) BMMo were pre-treated with the NLRP3 inhibitor MCC950 (950; 5 μM) or Q-VD-OPh (QVD; 40 μM) for 20–30 min, as indicated, prior to the addition of NOMVs (50 μg/ml), as specified, for a further 18 h. Cell death was measured by flow cytometric analysis of PI uptake.

C Sorted WT Ly6C^{hi} monocytes were treated with B5 LPS (50 ng/ml) for up to 5 h before immunoblot analysis of cell lysates for the indicated proteins.

D WT and A1-deficient ($A1^{-/-}$) Ly6C^{hi} monocytes were stimulated with NOMVs (50 μg/ml) or S63845 (S6; 10 μM) for 3 h. MOMP-induced loss of cytochrome-*c* staining was analysed by flow cytometric analysis.

E WT and A1-deficient ($A1^{-/-}$) Ly6C^{hi} monocytes were pre-treated with the NLRP3 inhibitor MCC950 (950; 5 μM), pan-caspase inhibitor Q-VD-OPh (QVD; 40 μM) and/or GSK'872 (872; 1 μM), as indicated, for 20–30 min prior to the addition of NOMVs (50 μg/ml) for a further 6 h. Cell supernatants and lysates were interrogated by immunoblot for relevant proteins.

F–H WT and Gasdermin D-deficient ($Gsdmd^{-/-}$) Ly6C^{hi} monocytes were pre-treated with the NLRP3 inhibitor MCC950 (950; 5 μM), pan-apoptotic caspase inhibitor Q-VD-OPh (QVD; 40 μM) and/or GSK'872 (872; 1 μM), as indicated, for 20–30 min prior to the addition of NOMVs (50 μg/ml) for a further 6 h. (F) IL-1β and (G) TNF levels were measured in culture supernatants by ELISA. (H) Cell death was measured by PI uptake via time-lapse IncuCyte imaging.

Data information: Each dot represents an individual (B, D, F, G) or the mean of three (H) biological replicates. Data are representative of at least two independent (B, D) or one (F–H) biological experiment and are presented as the mean + SEM or mean ± SEM. ns, not significant, *$P < 0.05$, **$P < 0.01$, ***$P < 0.005$, ****$P < 0.0001$ (two-way ANOVA with Tukey's multiple comparisons test). Blots represent three individual biological replicates (A) or at least three (C) or two (E) biological experiments; Ponceau stain was used as a loading control.
Source data are available online for this figure.

© 2023 The Authors

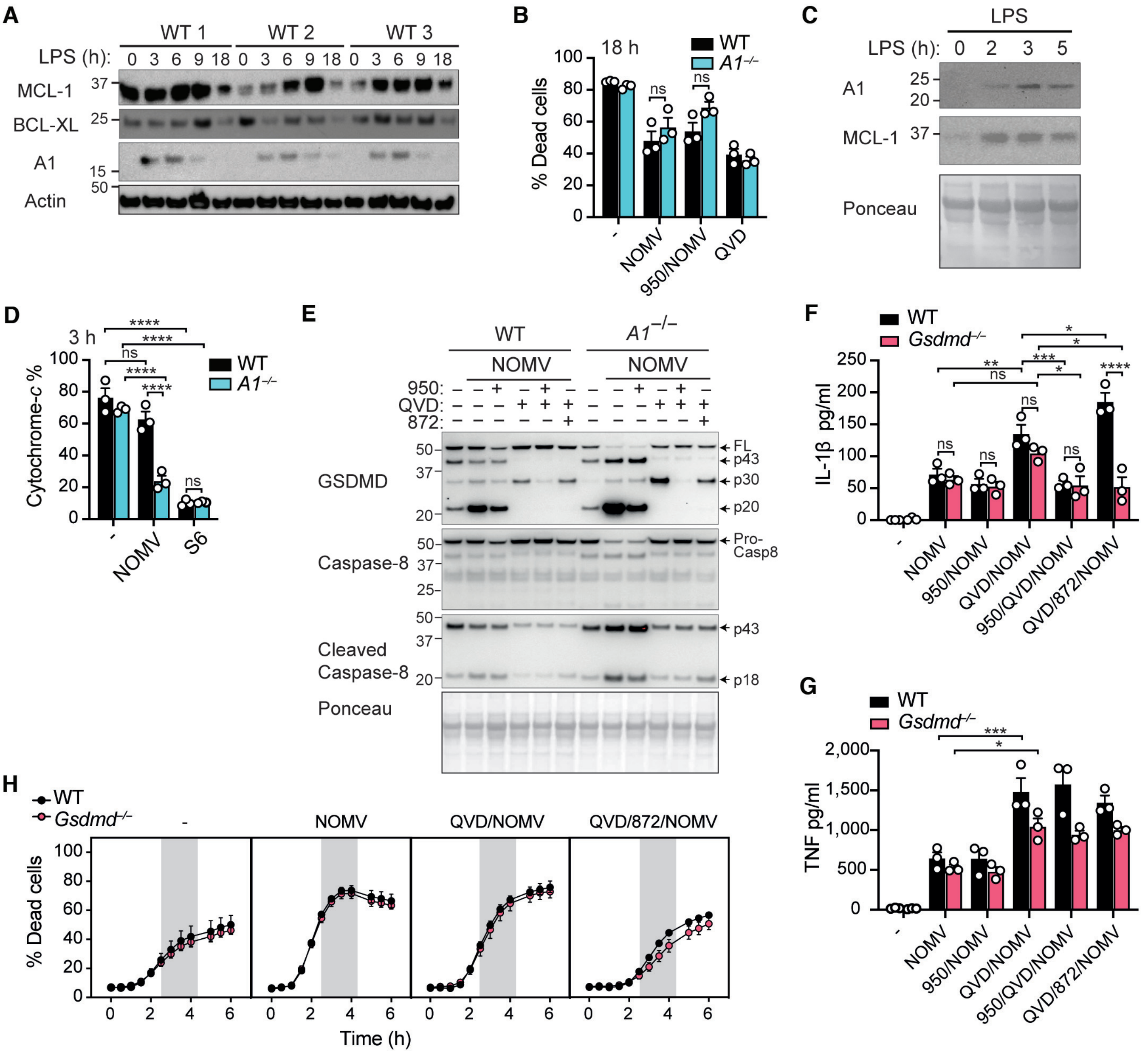

Figure EV4.

© 2023 The Authors

Figure EV5. Effect of A1 deficiency on systemic and local inflammatory responses to NOMV or LPS injection.

A–J WT and A1-deficient (*A1*$^{-/-}$) mice were injected intraperitoneally with 100 μg of NOMVs or PBS and peripheral blood and peritoneal lavage fluid harvested after 6 h. Immune cell subsets in the peripheral blood were quantified using (A, B) ADVIA or (C–F) flow cytometry. (A) Leukocyte counts and (B) populations as the % of total white blood cells (WBC). (C–F) Percentage % of peripheral blood leukocytes that are (C) neutrophils (CD11b^{+}Ly6G^{+}) and monocytes (CD11b^{+}Ly6G^{-}). (D) % of monocytes (CD11b^{+}Ly6G^{-}) that are inflammatory (Ly6C^{hi}CD62L^{hi}) or resident (Ly6C^{lo}CD62L^{lo}) subsets. (E) % of non-myeloid (CD11b^{-}) cells that are T cells (CD3^{+}) and B cells (B220^{+}). (F) Proportion % of (CD3^{+}) T cells that are CD4^{+} (CD3^{+}CD4^{+}) or CD8^{+} (CD3^{+}CD8^{+}) T cells. (G–J) Levels of (G, J) IL-6, (H) IL-1β, and (I) TNF were measured in the serum (G) and peritoneal lavage fluid (H–J) by ELISA.

K–M WT and A1-deficient mice (*A1*$^{-/-}$) were injected intraperitoneally with 100 μg of LPS and peritoneal lavage fluid harvested after 6 h. Levels of (K) IL-1β, (L) TNF, and (M) IL-6 were measured in the peritoneal lavage fluid by ELISA.

Data information: Each symbol represents an individual biological replicate. Data are expressed as mean ± SEM and are representative of one (A, B, G–J) or two pooled (C–F, K–M) biological experiments. *$P < 0.05$, **$P < 0.01$, ***$P < 0.005$, ****$P < 0.0001$ (A–J, one-way ANOVA with Tukey's multiple comparisons test) and (K–M, unpaired, two-tailed Student's *t*-test).
Source data are available online for this figure.

© 2023 The Authors

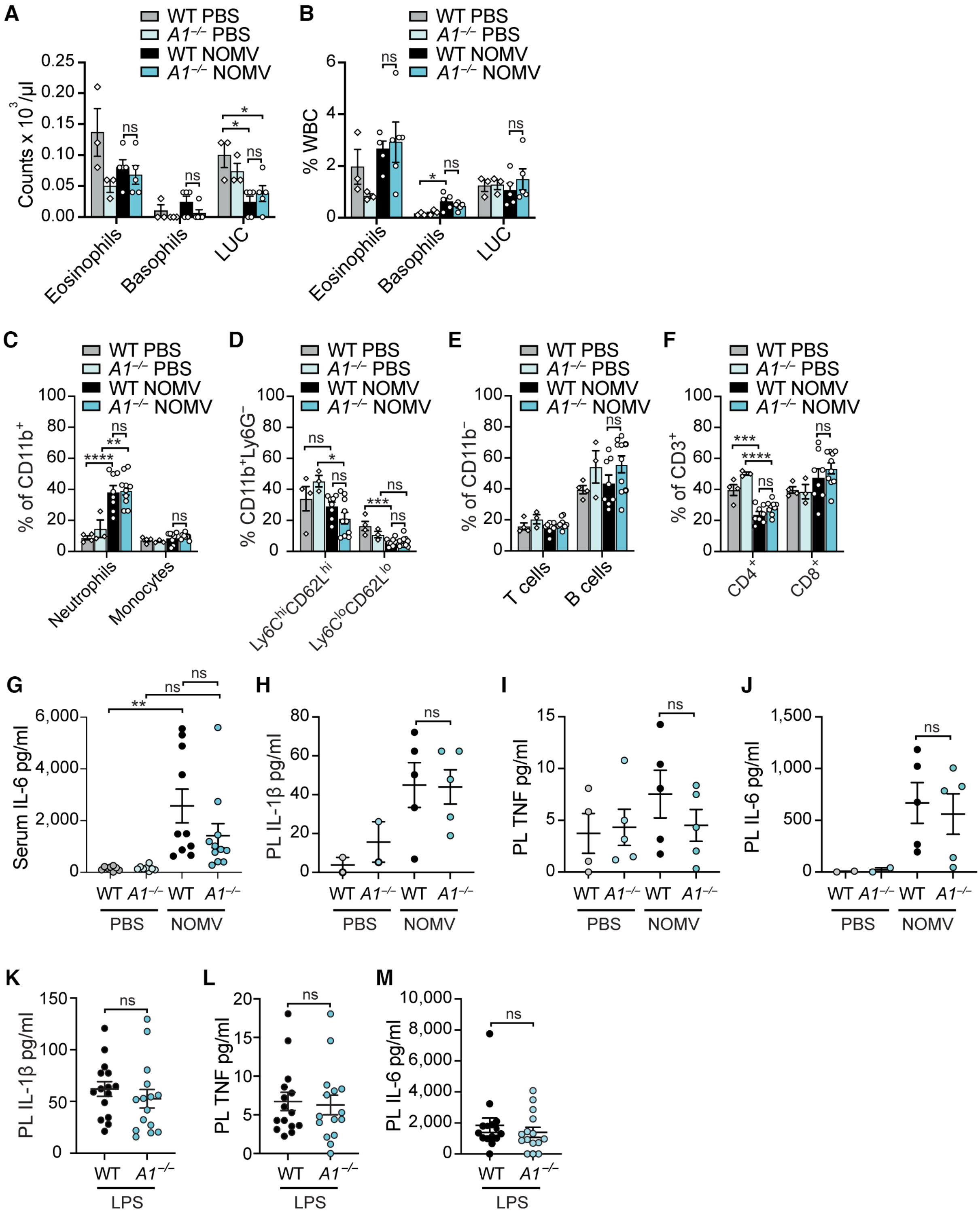

Figure EV5.

© 2023 The Authors
