# Supplementary figures and images for: A1 is induced by pathogen ligands to limit myeloid cell death and NLRP3 inflammasome activation
Source: EMBO Rep. 2023 Oct 17;24(11):e56865. doi: 10.15252/embr.202356865 (PMC10626451; doi:10.15252/embr.202356865)

Fig. EV1K

- WT and A1-/- BMDM
- Media, LPS

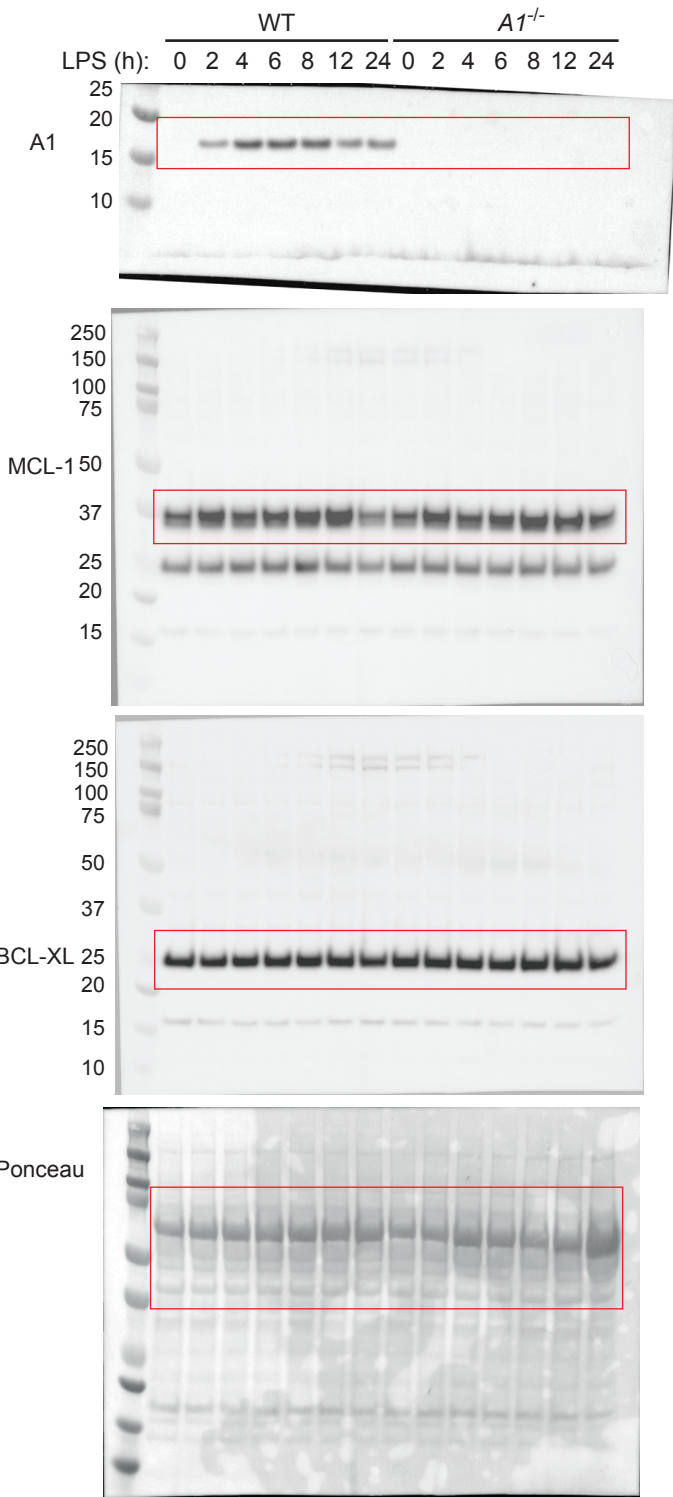

Supplement: Supplementary file 3 — Source Data for Expanded View and Appendix [file EMBR-24-e56865-s004.zip › EV and Appendix Figure Source Data/Figure EV1/Figure EV1K western blot.pdf]

**Fig. EV1L**

- WT and A1-/- BMDM
- Media, Cycloheximide (CHX) CHX, MG132, Bafilomycin A1 (BafA1) Q-VD-OPh (QVD)

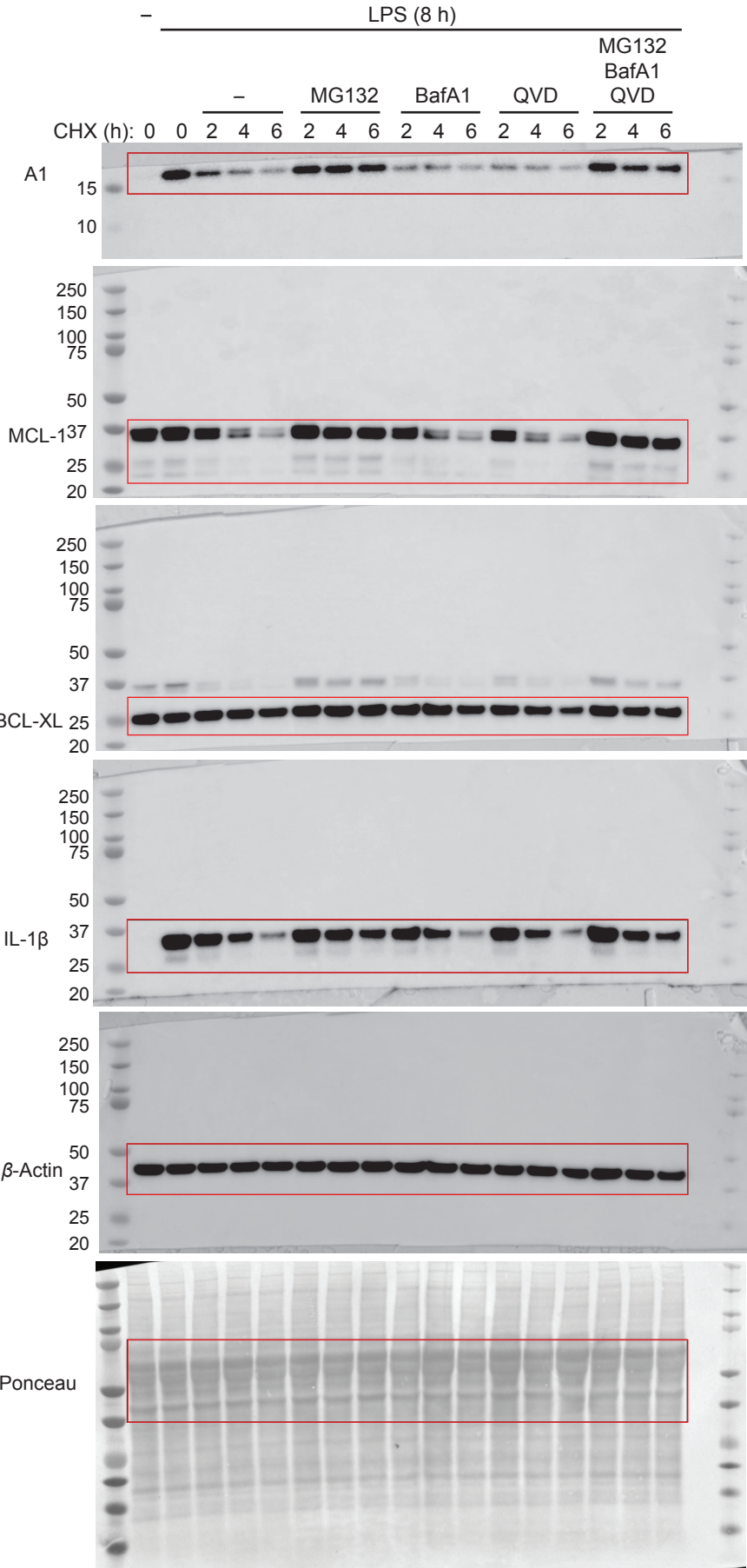

Supplement: Supplementary file 3 — Source Data for Expanded View and Appendix [file EMBR-24-e56865-s004.zip › EV and Appendix Figure Source Data/Figure EV1/Figure EV1L western blot.pdf]

**Fig. EV2G**

- WT and A1<sup>-/-</sup> BMDM
- Media, LPS, ABT-737 (737), S63845 (S6), MCC950 (950)

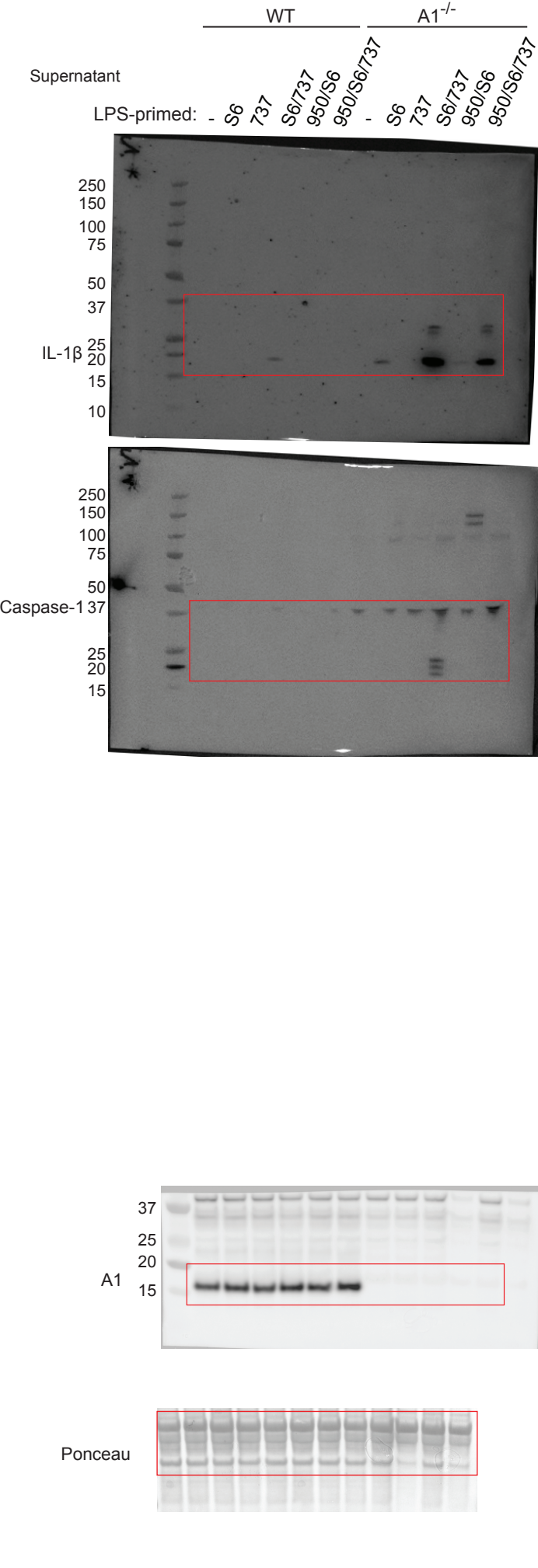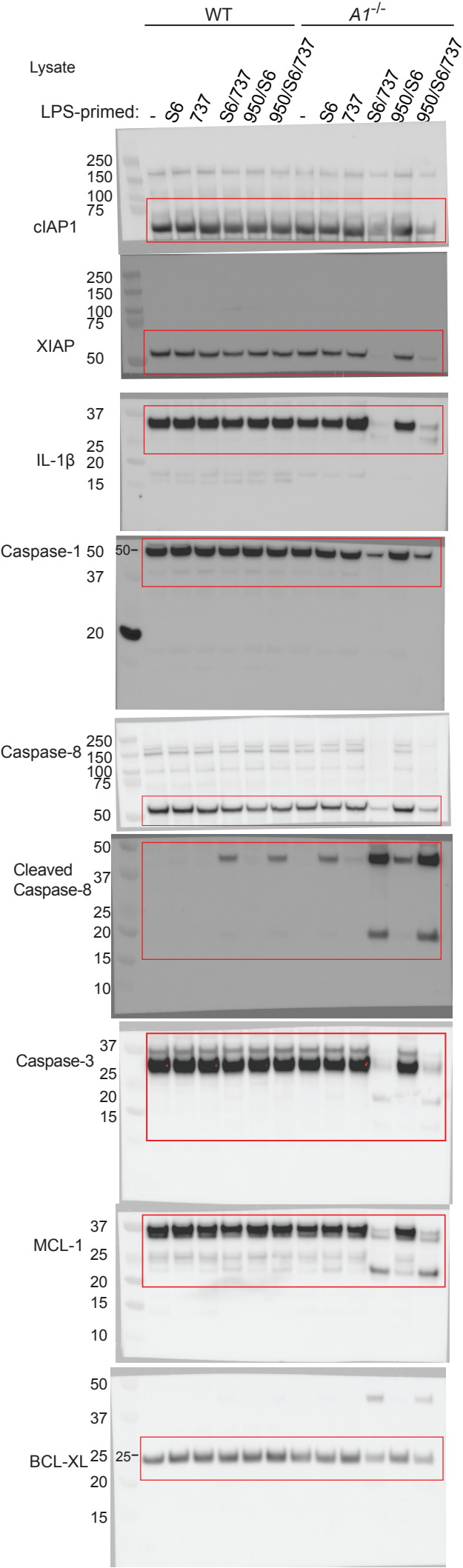

Supplement: Supplementary file 3 — Source Data for Expanded View and Appendix [file EMBR-24-e56865-s004.zip › EV and Appendix Figure Source Data/Figure EV2/Figure EV2G western blot.pdf]

Fig. EV3B

- WT and A1-/- BMDM
- Media, LPS, ABT-737 (737), S63845 (S6), Cycloheximide (CHX), Q-VD-OPh (QVD)

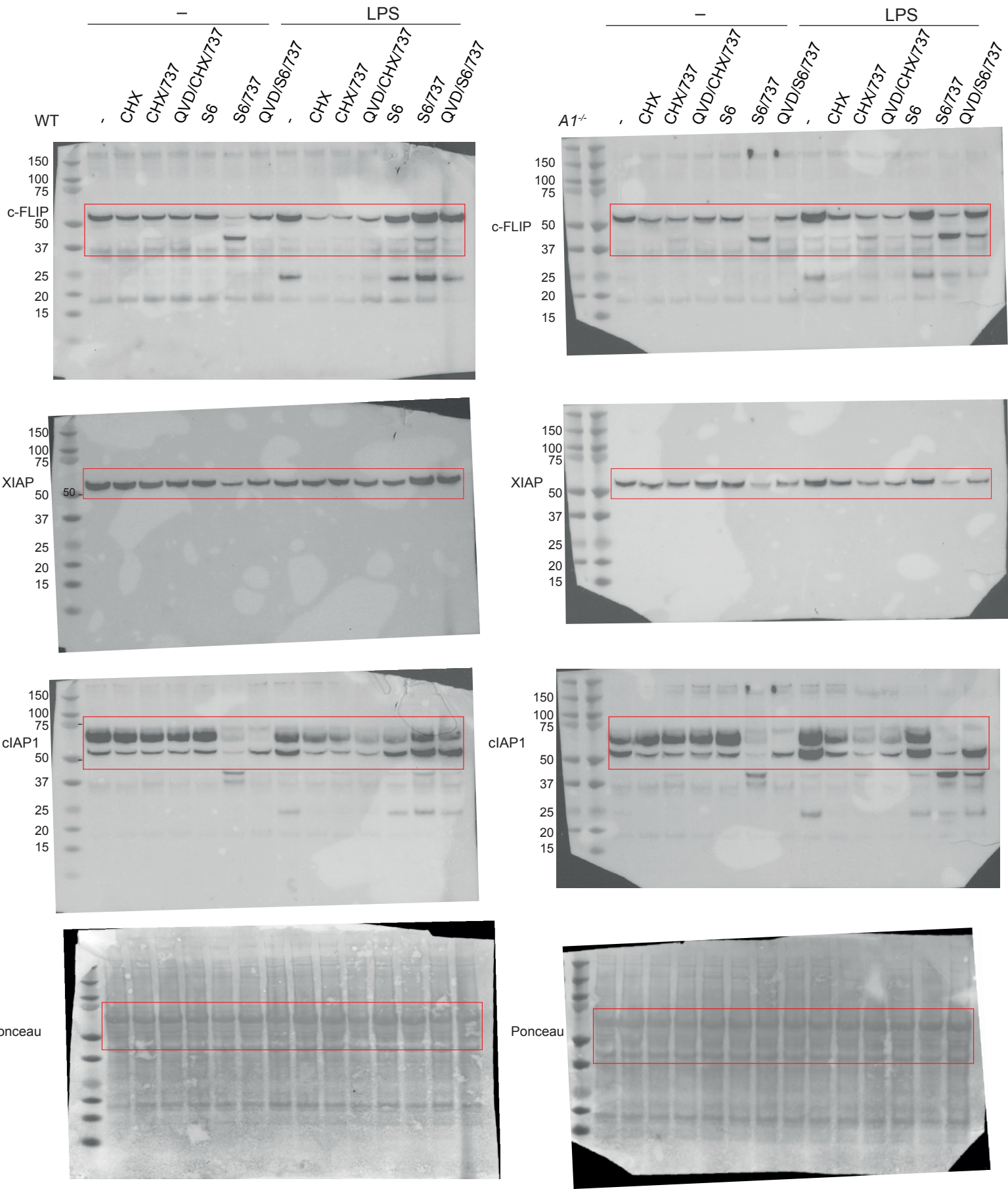

Supplement: Supplementary file 3 — Source Data for Expanded View and Appendix [file EMBR-24-e56865-s004.zip › EV and Appendix Figure Source Data/Figure EV3/Figure EV3B western blot.pdf]

**Fig. EV4A**

- WT BMMo
- Media, LPS

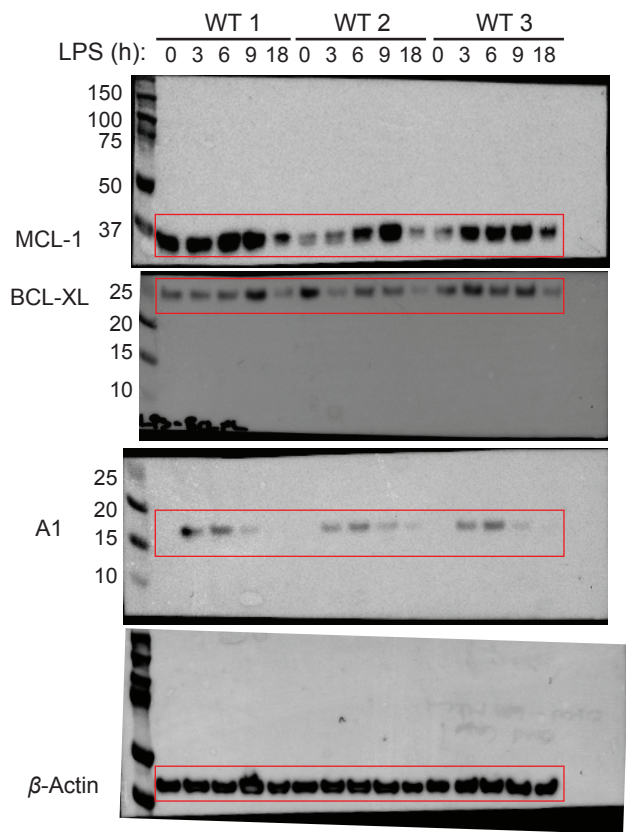

Supplement: Supplementary file 3 — Source Data for Expanded View and Appendix [file EMBR-24-e56865-s004.zip › EV and Appendix Figure Source Data/Figure EV4/Figure EV4A western blot.pdf]

**Fig. EV4C**

- WT Ly6C<sup>hi</sup> monocytes
- Media, LPS

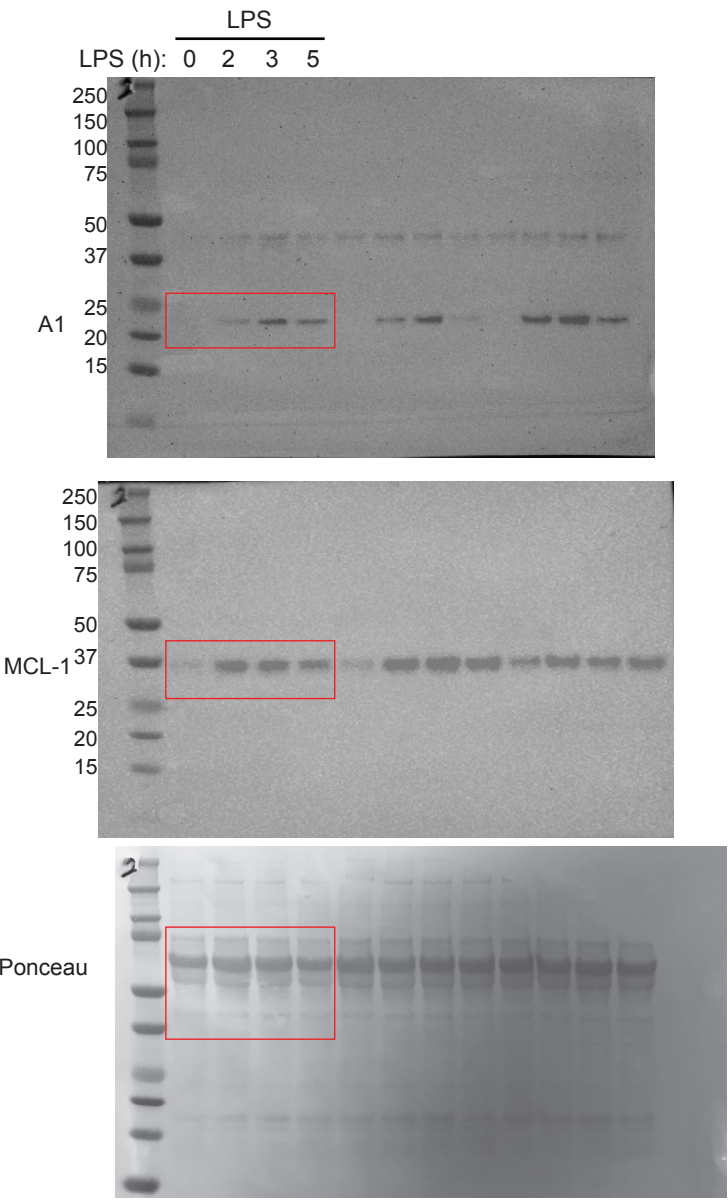

Supplement: Supplementary file 3 — Source Data for Expanded View and Appendix [file EMBR-24-e56865-s004.zip › EV and Appendix Figure Source Data/Figure EV4/Figure EV4C western blot .pdf]

Fig. EV4F

- WT Ly6C<sup>hi</sup> monocytes
- Media, NOMV, MCC950 (950), Q-VD-OPh (QVD), GSK'872 (872)

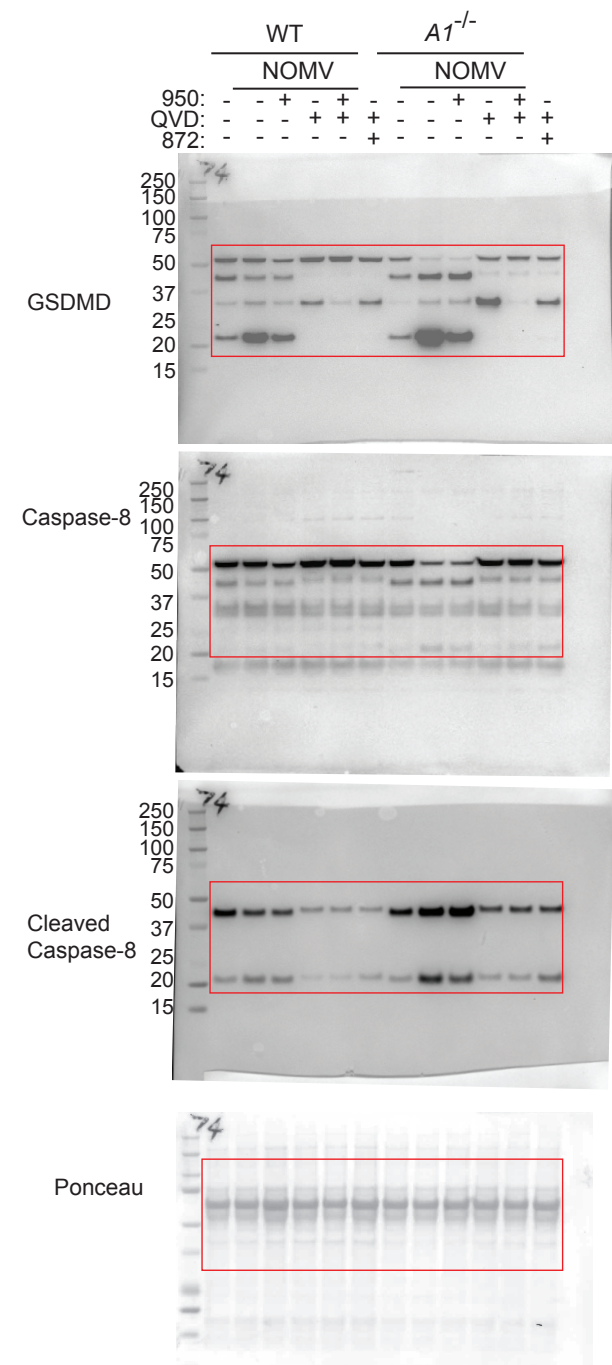

Supplement: Supplementary file 3 — Source Data for Expanded View and Appendix [file EMBR-24-e56865-s004.zip › EV and Appendix Figure Source Data/Figure EV4/Figure EV4E western blot.pdf]

- WT BMDMs
- Media, *Escherichia coli* O111:B4 (B4) and O55:B5 (B5) LPS serotypes

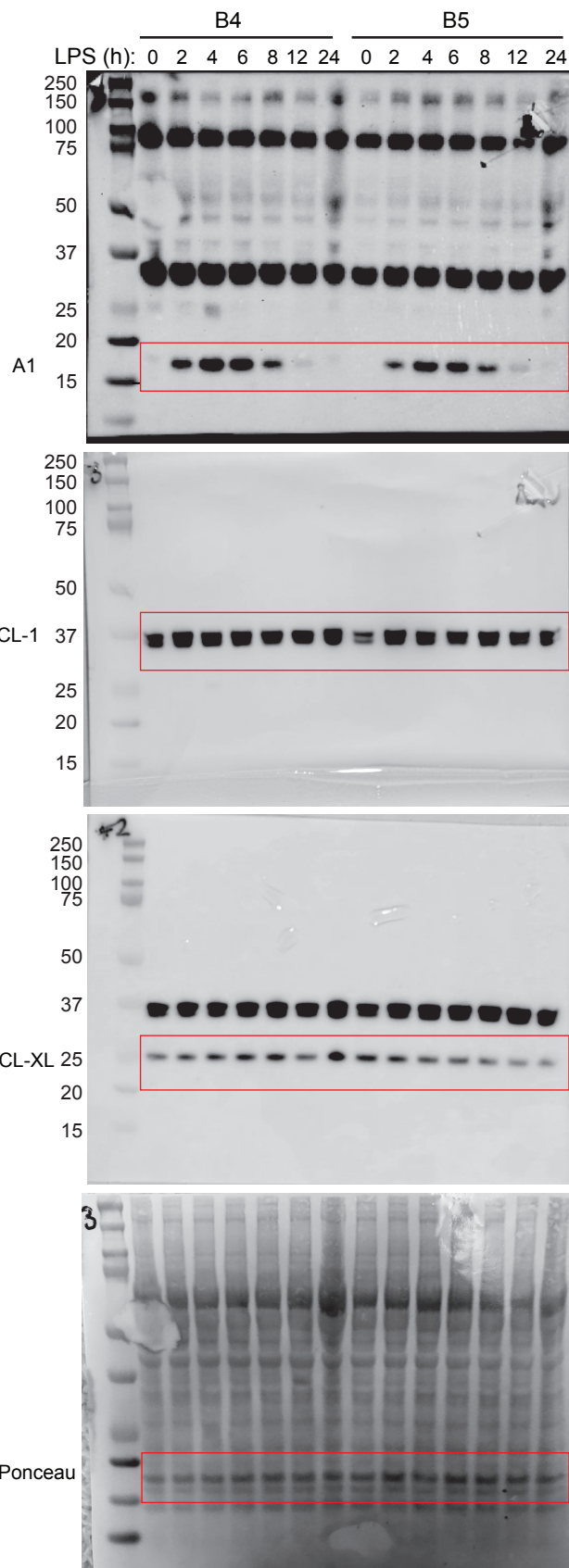

Supplement: Supplementary file 5 — Source Data for Figure 1 [file EMBR-24-e56865-s011.zip › Figure 1 /Figure 1G western blot.pdf]

**Figure 2B** • WT and A1-/- BMDMs  
• LPS, S63845 (S6), ABT-737 (737), MCC950 (950)

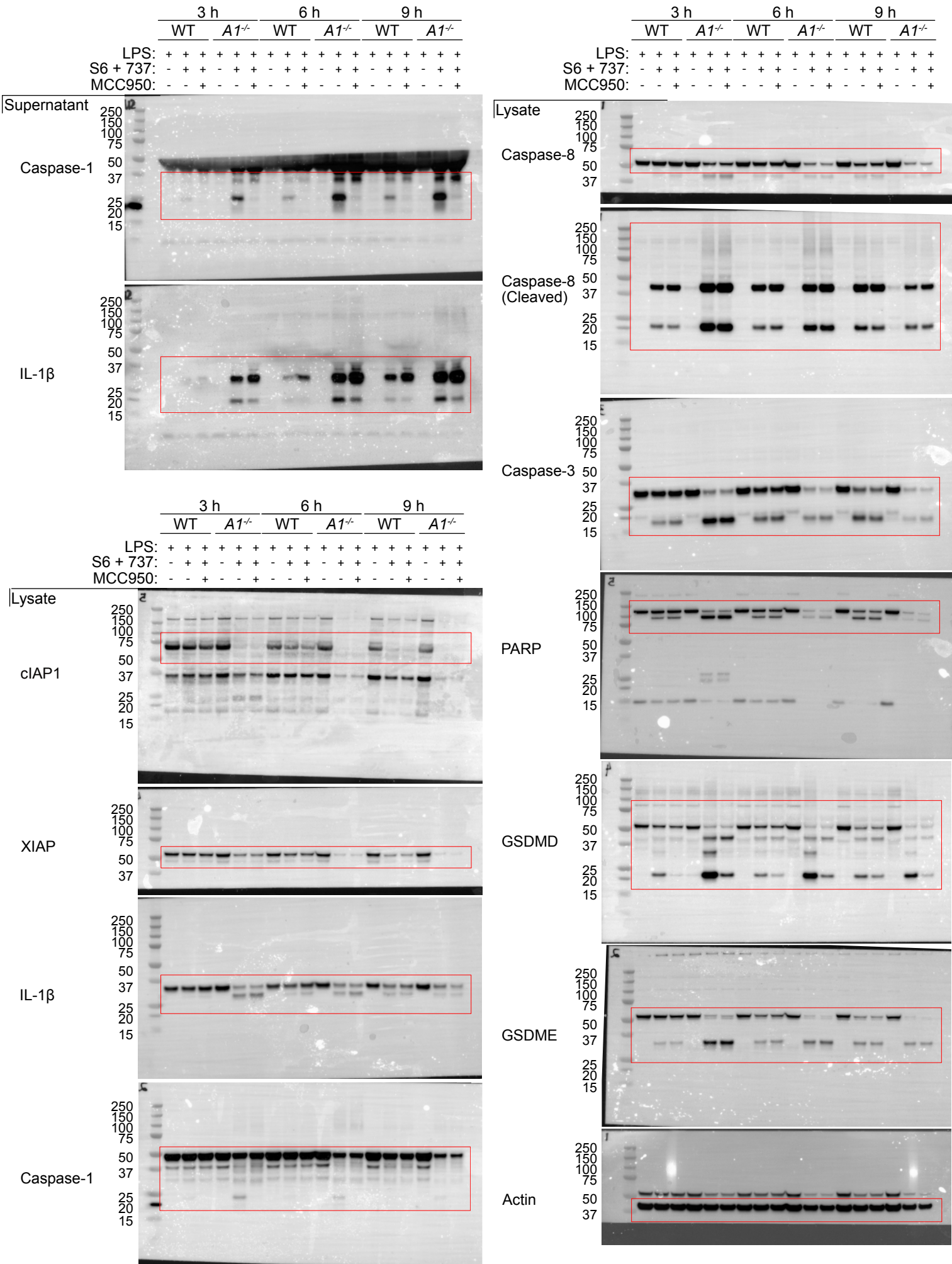

Supplement: Supplementary file 6 — Source Data for Figure 2 [file EMBR-24-e56865-s008.zip › Figure 2/Fig2B western blot.pdf]

**Figure 5D**

- WT and A1<sup>-/-</sup> BMMo
- Media, NOMV, MCC950 (950)

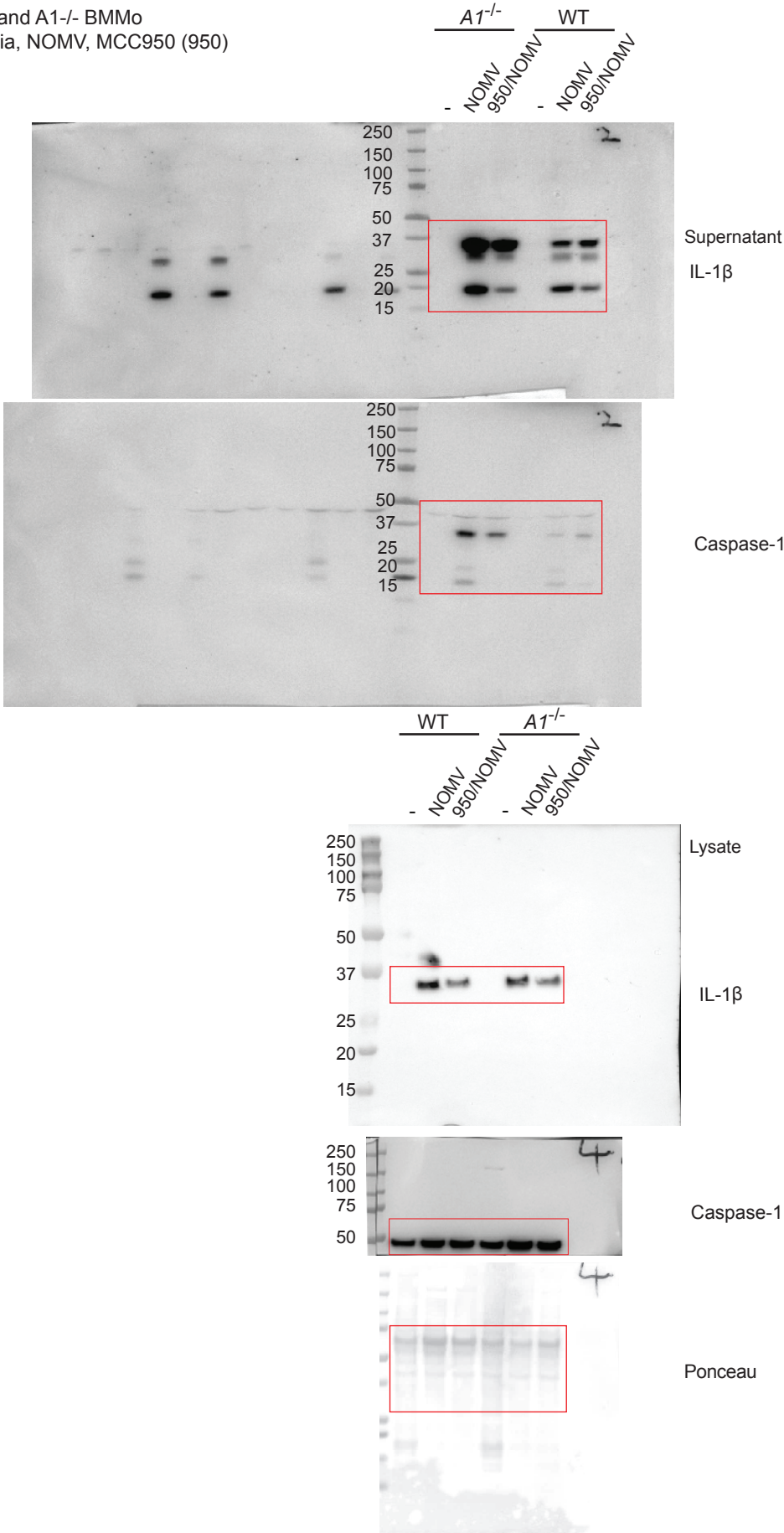

Supplement: Supplementary file 9 — Source Data for Figure 5 [file EMBR-24-e56865-s010.zip › Figure 5/Figure 5D western blot.pdf]

**Figure 5E**

- WT and A1-/- Ly6C<sup>hi</sup> monocytes
- Media, NOMV

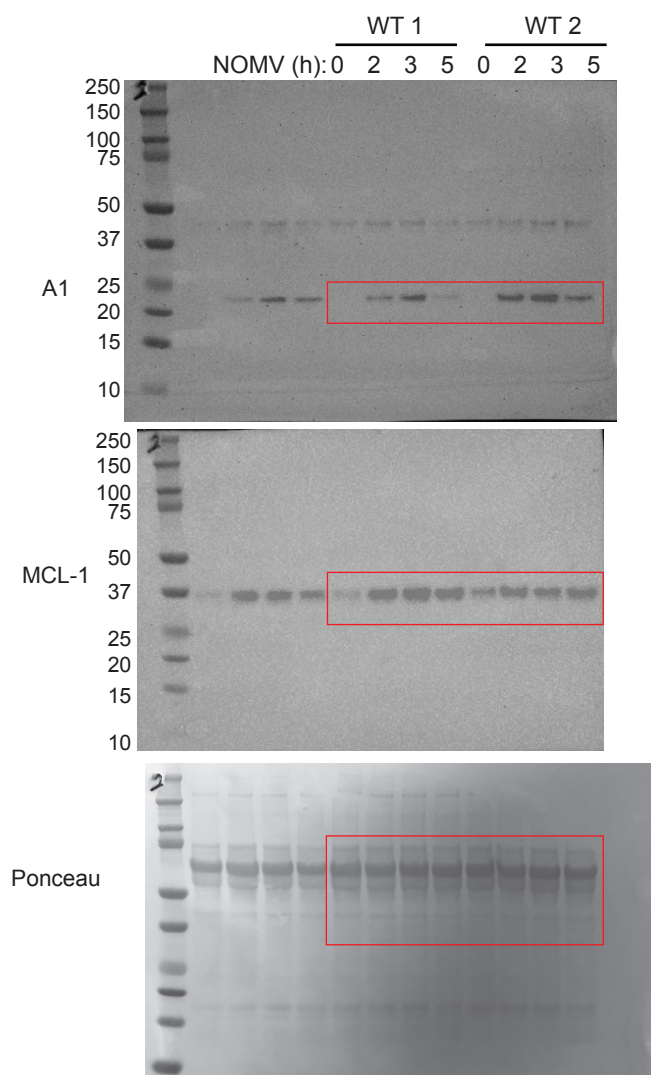

Supplement: Supplementary file 9 — Source Data for Figure 5 [file EMBR-24-e56865-s010.zip › Figure 5/Figure 5E western blot.pdf]

• WT and A1<sup>-/-</sup> Ly6C<sup>hi</sup> monocytes  
 • Media, NOMVs, MCC950 (950), Q-VD-OPh (QVD), GSK'872 (872)

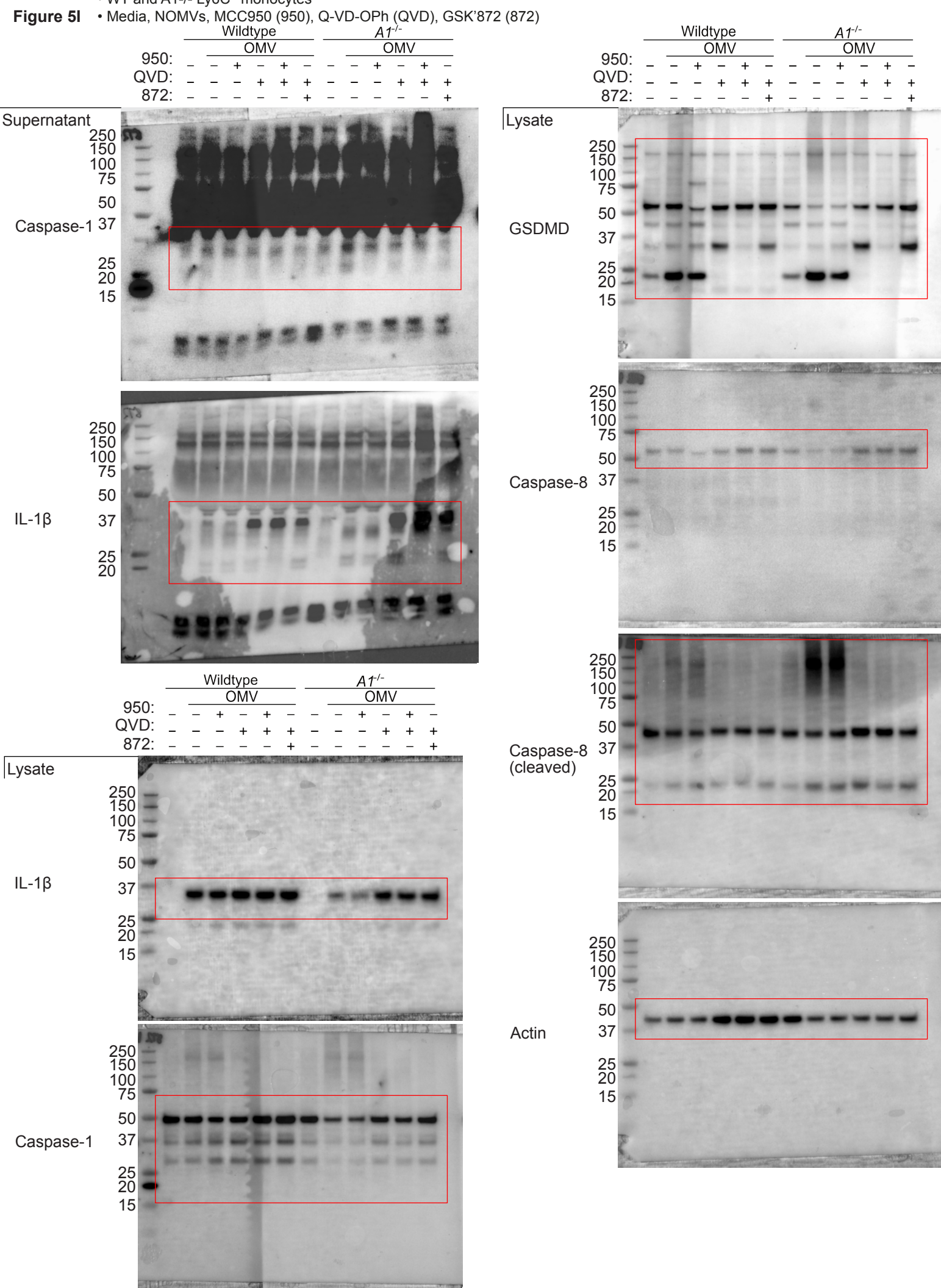

Supplement: Supplementary file 9 — Source Data for Figure 5 [file EMBR-24-e56865-s010.zip › Figure 5/Figure 5J western blot.pdf]

**Figure 5A**

- WT BMMo
- Media, NOMVs

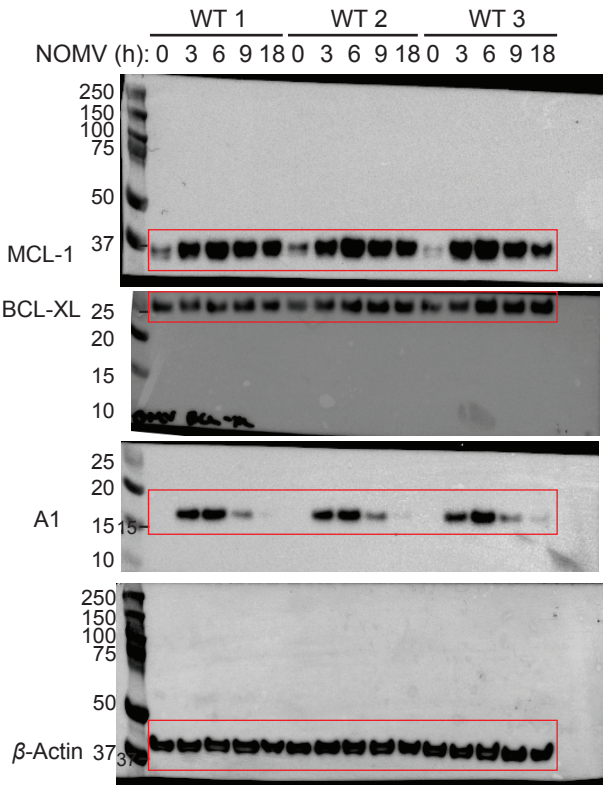

Supplement: Supplementary file 9 — Source Data for Figure 5 [file EMBR-24-e56865-s010.zip › Figure 5/Figure 5A western blot.pdf]

**Figure 6E**

- WT and A1<sup>-/-</sup> mice
- IP NOMV 100 µg

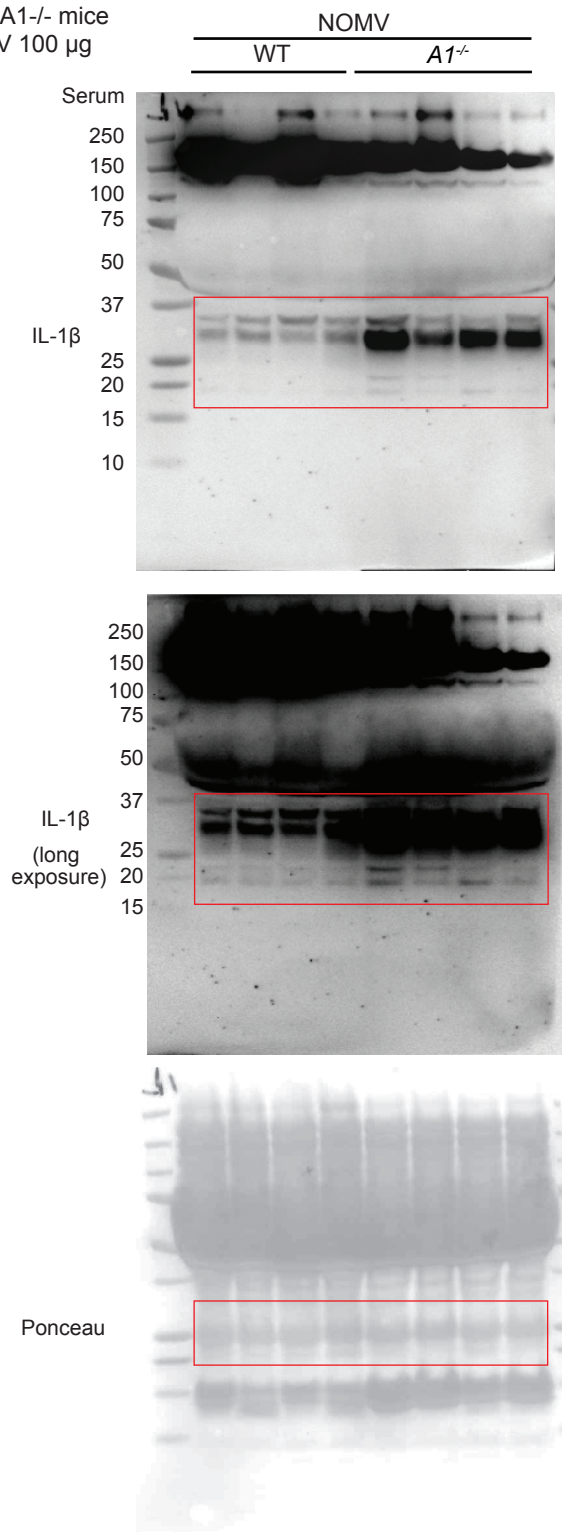

Supplement: Supplementary file 10 — Source Data for Figure 6 [file EMBR-24-e56865-s001.zip › Figure 6/Figure 6E western blot.pdf]
