# Supplementary material for: A1 is induced by pathogen ligands to limit myeloid cell death and NLRP3 inflammasome activation
Source: EMBO Rep. 2023 Oct 17;24(11):e56865. doi: 10.15252/embr.202356865 (PMC10626451; doi:10.15252/embr.202356865)
Supplement: Supplementary file 8 — Source Data for Figure 4 [file EMBR-24-e56865-s005.zip › Figure 4/Figure 4B western blot.pdf]

Figure 4B

- WT and A1-/- BMMo
- Media, LPS, ABT-737 (737)
ABT-199 (199), S63845 (S6)
MCC950 (950)

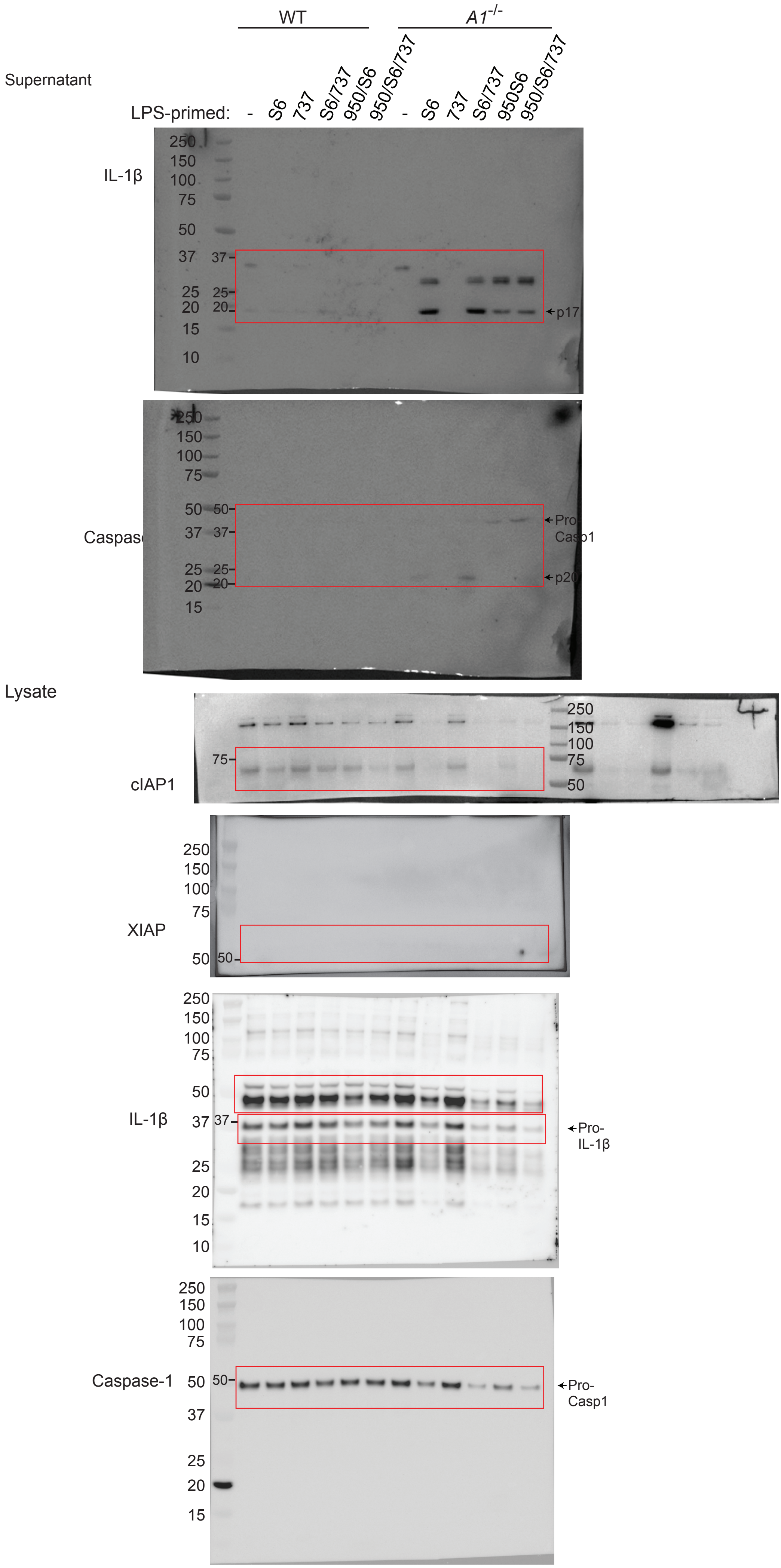

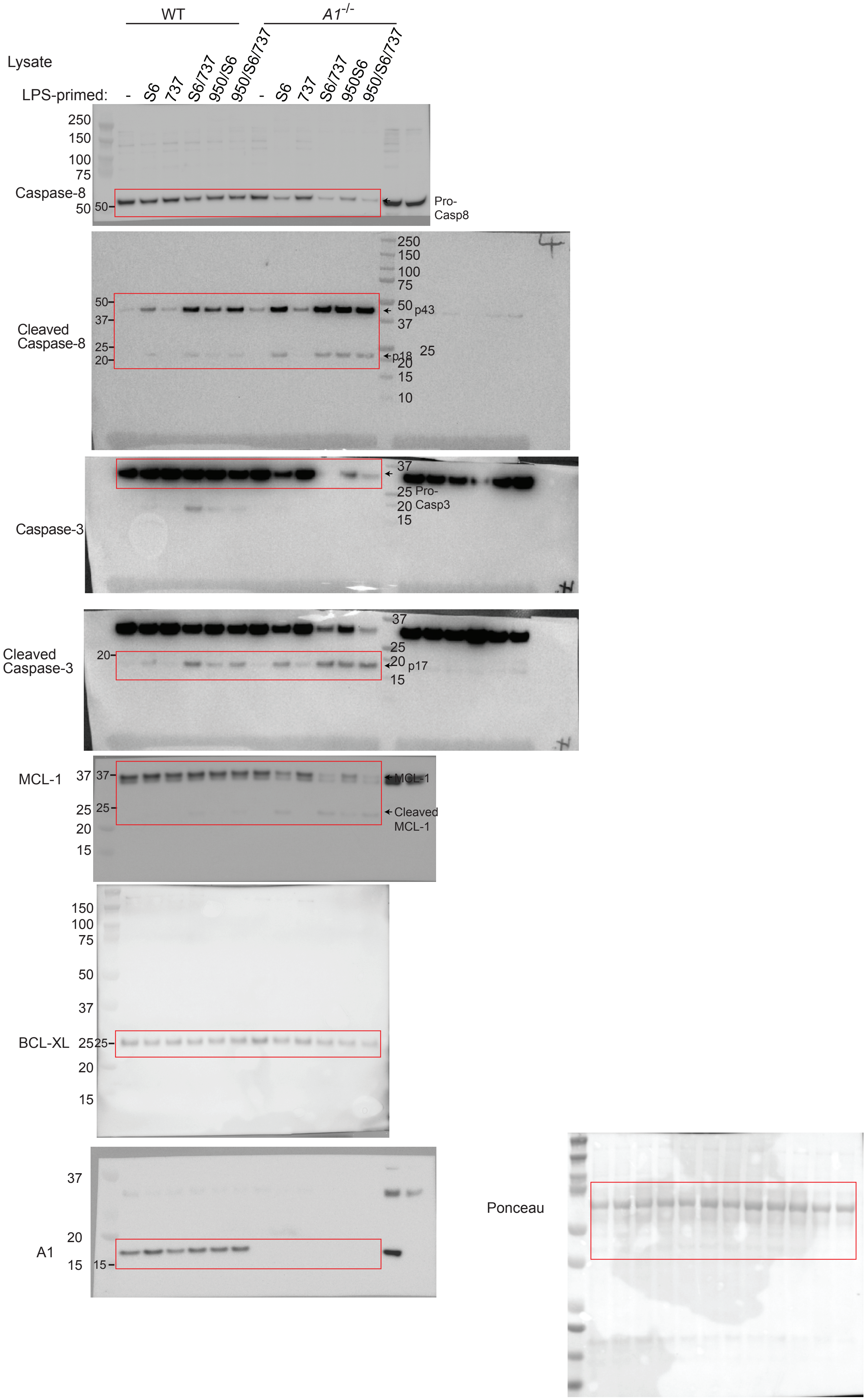

WT
A1-/-
Lysate
LPS-primed:
-
S6
737
S6/737
950/S6
950/S6/737
-
S6
737
S6/737
950S6
950/S6/737
Caspase-8
Pro-Casp8
Cleaved Caspase-8
p43
p18
Caspase-3
Pro-Casp3
Cleaved Caspase-3
p17
MCL-1
Cleaved MCL-1
BCL-XL
A1
Ponceau
250
150
100
75
50
37
25
20
15
10
