# Supplementary material for: A1 is induced by pathogen ligands to limit myeloid cell death and NLRP3 inflammasome activation
Source: EMBO Rep. 2023 Oct 17;24(11):e56865. doi: 10.15252/embr.202356865 (PMC10626451; doi:10.15252/embr.202356865)
Supplement: Supplementary file 11 [file EMBR-24-e56865-s009.pdf]

EMBO reports

A1 is induced by pathogen ligands to limit myeloid cell death and NLRP3 inflammasome activation

Mary Speir, Hazel Tye, Timothy Gottschalk, Daniel Simpson, Tirta Djajawi, Pankaj Deo, Rebecca Ambrose, Stephanie Conos, Jack Emery, Gilu Abraham, Ashlyn Pascoe, Sebastian Hughes, Ashley Weir, Edwin Hawkins, Isabella Kong, Marco Herold, Jaclyn Pearson, Najoua Lalaoui, Thomas Naderer, James Vince, and Kate Lawlor
DOI: 10.15252/embr.202356865

Corresponding author(s): Kate Lawlor (kate.lawlor@hudson.org.au)

Review Timeline:

Transfer Date:	23rd Jan 23
Editorial Decision:	25th Jan 23
Revision Received:	23rd Jul 23
Editorial Decision:	22nd Aug 23
Revision Received:	9th Sep 23
Accepted:	21st Sep 23

Editor: Achim Breiling

Transaction Report: This manuscript was transferred to EMBO *reports* following peer review at The EMBO Journal.

1st Editorial Decision 25th Jan 2023

Dear Dr. Lawlor

Thank you for transferring your manuscript to EMBO reports. I now went through your manuscript and the referee reports from The EMBO Journal (attached again below) and your p-b-p-response (revision plan). The referees have several concerns and suggestions to improve the manuscript, or to strengthen the data and the conclusions drawn.

EMBO reports emphasizes novel functional over detailed mechanistic insight, but asks for strong in vivo relevance of the findings, and clear experimental support of the major conclusions. Thus, we will not require addressing points regarding more mechanistic details experimentally. However, it will be necessary that during revision you address all points questioning the main conclusions and the novelty of the study, and all technical concerns, or points regarding the experimental designs, model systems used, or data presentation, by the addition of further data, text changes or good arguments in the rebuttal letter.

I thus invite you to revise your manuscript with the understanding that all concerns of the referees must be addressed in the revised manuscript or in a final detailed point-by-point response. Acceptance of your manuscript will depend on a positive outcome of another (final) round of review at EMBO reports, using the same referees.

Revised manuscripts should be submitted within three months of a request for revision. Please contact me to discuss the revision (also by video chat) if you have questions or comments regarding the revision, or should you need additional time.

When submitting your revised manuscript, please also carefully review the instructions that follow below.

PLEASE NOTE THAT upon resubmission revised manuscripts are subjected to an initial quality control prior to exposition to re-review. Upon failure in the initial quality control, the manuscripts are sent back to the authors, which may lead to delays. Frequent reasons for such a failure are the lack of the data availability section (please see below) and the presence of statistics based on n=2 (the authors are then asked to present scatter plots or provide more data points).

When submitting your revised manuscript, we will require:

1) a .docx formatted version of the final manuscript text (including legends for main figures, EV figures and tables), but without the figures included. Please make sure that changes are highlighted to be clearly visible. Figure legends should be compiled at the end of the manuscript text.

2) individual production quality figure files as .eps, .tif, .jpg (one file per figure), of main figures and EV figures. Please upload these as separate, individual files upon re-submission. Please make sure that all figure panels are called out separately and sequentially in the manuscript text

The Expanded View format, which will be displayed in the main HTML of the paper in a collapsible format, has replaced the Supplementary information. You can submit up to 5 images as Expanded View. Please follow the nomenclature Figure EV1, Figure EV2 etc. The figure legend for these should be included in the main manuscript document file in a section called Expanded View Figure Legends after the main Figure Legends section. Additional Supplementary material should be supplied as a single pdf file labeled Appendix. The Appendix should have page numbers and needs to include a table of content on the first page (with page numbers) and legends for all content. Please follow the nomenclature Appendix Figure Sx, Appendix Table Sx etc. throughout the text, and also label the figures and tables according to this nomenclature.

For more details please refer to our guide to authors:
http://www.embopress.org/page/journal/14693178/authorguide#manuscriptpreparation

See also our guide for figure preparation:
http://wol-prod-cdn.literatumonline.com/pb-assets/embo-site/EMBOPress_Figure_Guidelines_061115-1561436025777.pdf

See also the guidelines for figure legend preparation:
https://www.embopress.org/page/journal/14693178/authorguide#figureformat

3) a .docx formatted letter INCLUDING the reviewers' reports and your detailed point-by-point responses to their comments. As part of the EMBO Press transparent editorial process, the point-by-point response is part of the Review Process File (RPF), which will be published alongside your paper.

4) a complete author checklist, which you can download from our author guidelines (https://www.embopress.org/page/journal/14693178/authorguide). Please insert page numbers in the checklist to indicate where the requested information can be found in the manuscript. The completed author checklist will also be part of the RPF.

Please also follow our guidelines for the use of living organisms, and the respective reporting guidelines:
http://www.embopress.org/page/journal/14693178/authorguide#livingorganisms

5) that primary datasets produced in this study (e.g. RNA-seq, ChIP-seq and array data) are deposited in an appropriate public database. This is now mandatory (like the COI statement). If no primary datasets have been deposited in any database, please state this in this section (e.g. 'No primary datasets have been generated and deposited').

See also: http://embor.embopress.org/authorguide#datadeposition

Please remember to provide a reviewer password if the datasets are not yet public.

The accession numbers and database should be listed in a formal "Data Availability " section (placed after Materials & Methods) that follows the model below. Please note that the Data Availability Section is restricted to new primary data that are part of this study.

Data availability

The datasets produced in this study are available in the following databases:

- RNA-Seq data: Gene Expression Omnibus GSE46843 (https://www.ncbi.nlm.nih.gov/geo/query/acc.cgi?acc=GSE46843)
- [data type]: [name of the resource] [accession number/identifier/doi] ([URL or identifiers.org/DATABASE:ACCESSION])

*** Note - All links should resolve to a page where the data can be accessed. ***

Moreover, I have these editorial requests:

6) We now request the publication of original source data with the aim of making primary data more accessible and transparent to the reader. Our source data coordinator will contact you to discuss which figure panels we would need source data for and will also provide you with helpful tips on how to upload and organize the files.

7) Our journal encourages inclusion of *data citations in the reference list* to directly cite datasets that were re-used and obtained from public databases. Data citations in the article text are distinct from normal bibliographical citations and should directly link to the database records from which the data can be accessed. In the main text, data citations are formatted as follows: "Data ref: Smith et al, 2001" or "Data ref: NCBI Sequence Read Archive PRJNA342805, 2017". In the Reference list, data citations must be labeled with "[DATASET]". A data reference must provide the database name, accession number/identifiers and a resolvable link to the landing page from which the data can be accessed at the end of the reference. Further instructions are available at: http://www.embopress.org/page/journal/14693178/authorguide#referencesformat

8) Regarding data quantification and statistics, please make sure that the number "n" for how many independent experiments were performed, their nature (biological versus technical replicates), the bars and error bars (e.g. SEM, SD) and the test used to calculate p-values is indicated in the respective figure legends (also for potential EV figures and all those in the final Appendix). Please also check that all the p-values are explained in the legend, and that these fit to those shown in the figure. Please provide statistical testing where applicable. Please avoid the phrase 'independent experiment', but clearly state if these were biological or technical replicates. Please also indicate (e.g. with n.s.) if testing was performed, but the differences are not significant. In case n=2, please show the data as separate datapoints without error bars and statistics.
See also:
http://www.embopress.org/page/journal/14693178/authorguide#statisticalanalysis

If n<5, please show single datapoints for diagrams.

9) Please add up to 5 keywords below the abstract.

10) Please note our reference format:
http://www.embopress.org/page/journal/14693178/authorguide#referencesformat

11) We updated our journal's competing interests policy in January 2022 and request authors to consider both actual and perceived competing interests. Please review the policy https://www.embopress.org/competing-interests and add a statement declaring your competing interests. Please name that section 'Disclosure and Competing Interests Statement' and add it after the author contributions section.

12) Please order the manuscript sections like this using these names:
Title page - Abstract - Introduction - Results - Discussion - Materials and Methods - Data availability section (DAS) - Acknowledgements - Disclosure and Competing Interests Statement - References - Figure legends - Expanded View Figure legends

13) Please make sure that all the funding information is also entered into the online submission system and is complete and similar to the one in the manuscript text file (acknowledgements).

14) We now use CRediT to specify the contributions of each author in the journal submission system. CRediT replaces the author contribution section. Please use the free text box to provide more detailed descriptions. Thus, please remove the author contributions section from the manuscript text file. See also guide to authors:
https://www.embopress.org/page/journal/14693178/authorguide#authorshipguidelines

I look forward to seeing a revised version of your manuscript when it is ready. Please let me know if you have questions or comments regarding the revision.

Finally, please note that all corresponding authors are required to supply an ORCID ID for their name upon submission of a revised manuscript. Please find instructions on how to link the ORCID ID to the account in our manuscript tracking system in our Author guidelines: http://www.embopress.org/page/journal/14693178/authorguide#authorshipguidelines

Kind regards,

Achim

Achim Breiling
Senior Editor
EMBO Reports

Referee #1:

In this ms. Speir and colleagues identify A1 (BFL-1) as a key regulator of myeloid cell death/NLRP3 inflammasome activation in response to pathogen sensing. Based on LPS priming, they demonstrate an upregulation of A1, confirming functional relevance using A1 ko mice (whereby loss of A1 promotes inflammation/cell death in response to BH3-mimetic treatment). This is then investigated in a more physiologically relevant model using OMVs derived from Neisseria, essentially supporting the in vitro validation. In my opinion, this study offers important new insight into apoptotic control of inflammation, specifically the role of A1 in this, and has clear relevance for pathogen control. On the whole, the data support the authors' conclusions, however some aspects require additional clarification. These are highlighted below (in no order of importance).

- figure 1, there is reference to LPS-priming affecting cell death in response to MCL-1 inhibition (page 5), based on the data presented (fig 1) would suggest that the authors mean to refer to S6/737 combo? (not MCL-1 inhibitor alone - this seems to not cause any cell death).

- line 107 doesn't make much sense "we observed that specific MCL-1 inhibition with S63845 could delay cell death and consequent IL-1β release in LPS-primed cells, despite efficient priming" - again, think authors must be referring to combo treatment, but regardless, it reads back to front, it's the LPS-priming that is retarding cell death as opposed to the BH3-mimetic addition (as it reads here).

- definition of caspase-8 activity in figure 2 as far as I can see is via cleavage of caspase-8, this is not conclusive evidence of activity since caspase-3/7 can cleave caspase-8 (and cleavage of c8 per se does not activate it). In the absence of using additional approaches to demonstrate C8 activation, suggest the authors temper their conclusion of caspase-8 activity.

- with respect to deciphering residual necroptosis, others have shown that necroptosis can be engaged following MOMO dependent on TNF synthesis (see PMID: 28846096), is TNF required for necroptosis observed in the current study ?

Referee #2:

Here the authors show that LPS-mediated upregulation of A1 modestly influences LPS-mediated death of macrophages (BMDMs) and monocytes, but only upon concurrent neutralization of Mcl-1 and Bcl-x (macrophages) or Mcl-1 (Monocytes) using BH3 mimetics. The role of A1 in regulating LPS-induced survival (and IL-1beta production downstream) on its own was negligible. The data, as presented, are sound and well interpreted. However, I don't feel that these observations rise to the level of significance that would warrant publication in the EMBO Journal for a number of reasons.

It is well known that pro-inflammatory stimuli, such as LPS and TNF, upregulate A1 (e.g. Hu et al., 1998 Blood 92, 2759). Loss of

A1 by itself appears to play only a very minor role in regulating cell death and inflammasome activation downstream of LPS, with Mcl-1 and Bcl-xL playing much more dominant roles in this context (as evidenced by the robust increases in cell death seen upon treatment with S6/ABT737 in combination). The effects of A1 loss are only revealed when Mcl-1 and Bcl-xl are neutralized using synthetic BH3-mimetics, or in combination with protein synthesis blockade. I'm not persuaded that this represents any particular physiological scenario.

The effects of A1 loss were somewhat more significant when inflammation was driven with very high concentration of NOMVs (50 ug per mL), that effectively block protein synthesis, but it is not at all surprising that loss of a pro-survival Bcl-2 family member leads to accelerated cell death under certain conditions. Therefore, although the study is nicely conducted and the data are well described and presented, the findings reported herein do not have the novelty or significance that would warrant publication in the EMBO Journal and are more suited to publication in a specialist journal.

Referee #3:

To understand the crosstalk between cell death and pathogen-induced innate immune responses, in this study, Speir et al demonstrated that the pro-survival BCL-2 family member A1, induced by pathogen ligands limits BAX/BAK-dependent apoptosis and IL-1β release in macrophages and inflammatory Ly6Chi monocytes. They also show that the survival modulator A1 in inflammatory monocytes delays death modalities and IL-1β activation triggered by Neisseria gonorrhoeae-derived outer membrane vesicles (NOMVs), exemplified by heightened IL-1β secretion into serum in response to NOMV injection in A1-deficient mice, advancing the development of myeloid cell-targeted antimicrobial therapeutics. This mechanistic study demonstrating A1 in controlling the timing of cell death and IL-1β production upon infection is important but more details are needed to strengthen the manuscript and make it suitable for a general interest journal.

Major concerns:

1. The whole study is based on the notion that "Loss of MCL-1 and BCL-XL (or BCL-2) activity in LPS-primed macrophages can trigger BAX/BAK-dependent inflammation associated with the loss of ripoptosome repressors XIAP and cIAP1, and through effector caspase-3/-7-mediated activation of caspase-8 to directly cleave IL-1β [23, 25, 63]" (Text line 335 to 339) and authors proposed A1 restricts NLRP3 inflammasome-dependent/independent IL-1β secretion via MCL-1-BAX/BAK-caspase 3/7 axis (Fig 5I). However, mitochondrial dysfunction coupled with MOMP can lead to inflammasome activation independent of caspase 8, caspase 3 or caspase 7. This should be addressed and discussed.

https://www.embopress.org/doi/full/10.15252/embr.201949799

2. The authors show that A1 and MCL-1 are key survival proteins in monocytes in sensing LPS or OMV while macrophages rely on A1, BCL-XL and MCL-1to prevent cell death (Fig 2-4 and Fig 5I). The physiological significance underlying this discrepancy between different types of inflammatory myeloid cells in response to infection needs to be discussed and linked with the in vivo results in Fig 5.

3. This study emphasized the control of A1 in "timing/delaying" cell death and IL-1β production. Therefore, kinetic studies of caspases activation and NLRP3 inflammasome dependent/independent IL-1β production are crucial to make the whole mechanistic process clear.

For example, Fig 4H is confusing. According to Fig 4E, expression level of A1 protein in WT monocytes at 5 h after NOMV treatment was relatively low, why would the authors choose "5 h" as an optimal timepoint to compare IL-1β production between WT vs A1 KO monocytes in Fig 4H?

4. Are cell death and IL-1β production tightly linked/synchronized? In Fig 2B, cell death in S6/737 group between WT BMDMs and A1 KO BMDMs was similar, whereas in Fig 2E, IL-1β production was much more robust in A1 KO BMDMs, compared to WT BMDMs. Is IL-1β production here unrelated to caspase 8 or a "delayed" response? This should be discussed.

Minor concerns:

1. In addition to IL-1β production, how does caspase 8 modulate pyroptotic pore formation for IL-1β secretion? In Fig 4J and Fig S4F, the author show GSDMD p30 was elevated when apoptotic caspases were inhibited by QVD, suggesting apoptotic caspases limit GSDMD N-terminal p30 cleavage and pyroptosis. How does this link to IL-1β secretion? This should be addressed.

2. The PI uptake measured throughout the whole manuscript to demonstrate "apoptosis" may need to be revised. This method would indicate diverse types of cell death including necroptosis and pyroptosis.

3. Why was IL-1β production increase in A1 KO mice only observed in serum samples (Fig 5) but not peritoneal lavage fluid (Fig

S5) when the acute inflammatory responses were assessed after 6 h of intraperitoneal injection of NOMV. This should be discussed.

4. The accuracy of description needs to be improved. For example, in Fig 2 C and Fig 3C, it is not precise to state "cytochrome-c release were assessed by flow cytometric analysis as a measure of cell death and MOMP, respectively.". What the authors show in the figure was remaining cyto-c rather than released cyto c signal.

It is not accurate to state "Crucially, IL-1β secretion was almost completely abrogated by combined pre-treatment with MCC950 and Q-VD-OPh (Fig. 4H, 4J), confirming that caspase-1 and caspase-8 were both required for full IL-1β activity." (Line 286-288). Q-VD-OPh is pan-caspase inhibitor suppressing several caspases in addition to caspase 8.

5. Data inconsistency needs to be addressed. In Fig S2B and Fig S2D, IL-1β was reduced by A1 loss compared to WT.

RESPONSE TO REVIEWERS - EMBOR-2023-56865-T

We thank the editor and reviewers for their constructive comments. We have carefully revised our manuscript EMBOR-2023-56865-T entitled "**A1 is induced by pathogen ligands to limit myeloid cell death and NLRP3 inflammasome activation"** for clarity of language, as requested and have updated figures and text to match with the *EMBO reports* guidelines. Changes to the text are underlined in the manuscript with updated references. We also provide a point-by-point rebuttal addressing all the reviewer comments/questions shown in italics below.

We would like to highlight that our study is the first to show physiological relevance for the anti-apoptotic BCL-2 family member, *Bcl2a1* (A1) during infection. We make a number of novel observations including:

1. Upregulation of A1 in LPS-primed macrophages and inflammatory monocytes delays apoptotic cell death and downstream NLRP3 inflammasome- and caspase-8-mediated activation of IL-1β.
2. Unlike macrophages, monocyte survival in response to pathogen sensing is regulated by the short-lived pro-survival BCL-2 family members MCL-1 and A1.
3. A1-deficient macrophages and monocytes undergoing rapid BAX/BAK-induced apoptosis show cell death plasticity by engaging with the necroptotic pathway when apoptotic caspase activity is compromised. In this scenario, necroptotic signalling also triggers NLRP3 inflammasome activation which further defines the inflammatory potential of BAX/BAK signalling.
4. Outer membrane vesicles (OMVs) derived from *N. gonorrhoea* that target the mitochondria to activate BAX and BAK also upregulate A1 in inflammatory monocytes to delay apoptotic cell death and IL-1β activation triggered by caspase-8 and NLRP3-caspase-1 activity;
5. A1-deficient mice have a heightened acute inflammatory response associated with increased IL-1β production upon *in vivo* challenge with *N. gonorrhoea*-derived OMVs (NOMVs).

We note that the reviewers expressed admiration for the quality of the biochemical data and the conclusions presented and hope that our revised study, which incorporates robust and exciting new data, will make this manuscript suitable for publication in *EMBO Reports*. We believe our work significantly enhances our understanding of myeloid cell death, as it documents the plasticity and inflammatory crosstalk that exists downstream of intrinsic BAX/BAK activation during pathogen signalling.

Referee #1:

R#1. *In this ms. Speir and colleagues identify A1 (BFL-1) as a key regulator of myeloid cell death/NLRP3 inflammasome activation in response to pathogen sensing. Based on LPS priming, they demonstrate an upregulation of A1, confirming functional relevance using A1 ko mice (whereby loss of A1 promotes inflammation/cell death in response to BH3-mimetic treatment). This is then investigated in a more physiologically relevant model using OMVs derived from Neisseria, essentially supporting the in vitro validation. In my opinion, this study offers important new insight into apoptotic control of inflammation, specifically the role of A1 in this, and has clear relevance for pathogen control. On the whole, the data support the authors' conclusions, however some aspects require additional clarification. These are highlighted below (in no order of importance).*

Response 1. We thank the reviewer for their positive feedback on the quality of the data, as well as their recognition that this paper provides important new insights into the regulation of apoptosis and inflammatory signalling during infection.

R#1. *- figure 1, there is reference to LPS-priming affecting cell death in response to MCL-1 inhibition (page 5), based on the data presented (fig 1) would suggest that the authors mean to refer to S6/737 combo? (not MCL-1 inhibitor alone - this seems to not cause any cell death).*

Response 2. The reviewer is correct. The text in this section (lines 119-129) has been amended for clarity in the revised manuscript.

R#1. - *line 107 doesn't make much sense "we observed that specific MCL-1 inhibition with S63845 could delay cell death and consequent IL-1β release in LPS-primed cells, despite efficient priming" - again, think authors must be referring to combo treatment, but regardless, it reads back to front, it's the LPS-priming that is retarding cell death as opposed to the BH3-mimetic addition (as it reads here).*

Response 3. The text in this section (lines 119-129) has been amended for clarity in the revised manuscript.

R#1. - *definition of caspase-8 activity in figure 2 as far as I can see is via cleavage of caspase-8, this is not conclusive evidence of activity since caspase-3/7 can cleave caspase-8 (and cleavage of c8 per se does not activate it). In the absence of using additional approaches to demonstrate C8 activation, suggest the authors temper their conclusion of caspase-8 activity.*

Response 4. Cleavage of caspase-8 to its p43 and p18 forms is often used as a marker of its activation. However, we have now amended the wording in the text to 'cleavage of caspase-8' to better reflect the western blot data. We wish to highlight for the reviewers benefit that we do show further evidence of the active status of caspase-8, as we often observe modification of cleaved caspase-8 in LPS/ABT-737/S63845 treated A1-deficient BMDMs, which is thought to be ubiquitylation that is linked to enhanced caspase-8 function (Jin Z *et al.* 2009 *Cell* 137:721 PMID:19427028) [*e.g.*, **Rebuttal Fig. 1, Fig. EV2G of the revised manuscript that was Fig. 2H** (NB. Blot cut through modification to probe for other proteins of interest) **and NEW data Fig. 2B and 5I of the revised manuscript**], and we see cleavage of the caspase-8 substrate c-FLIP (**Fig. EV3B of the revised manuscript**). Further, we wish to note that we can assert that caspase-8 is active in this setting, as we show in **Fig. 2B, 2E, EV2C, EV2D, EV2G, EV2H and EV2I** that addition of the NLRP3 inhibitor, MCC950, abrogates BAX/BAK-dependent caspase-1 cleavage but does not completely block IL-1β activation and secretion in A1-deficient cells. This is in line with our previous study where we showed genetically that BAX/BAK signalling induces caspase-3/7-mediated cleavage and activation of caspase-8 to trigger NLRP3-independent IL-1β activation (Vince JE *et al.* 2018 *Cell Rep* 25:2339 ref#28). Noting that, of the caspases, active caspase-8 and caspase-1 share a number of common substrates, including, for example, IL-1β, GSDMD, and BID (*e.g.*, Vince JE *et al.* 2012 *Immunity* 36:215 ref#68; Orning P *et al.* 2018 *Science* 362:1064 ref#66; Chen KW *et al.* 2019 *EMBO J* 38:e101 ref#55; Heilig R *et al.* 2020 *Life Science Alliance* 3:e101000735 PMID:32345661, Hughes SA *EMBO J* 42(5):e110468 PMID: 36647747).

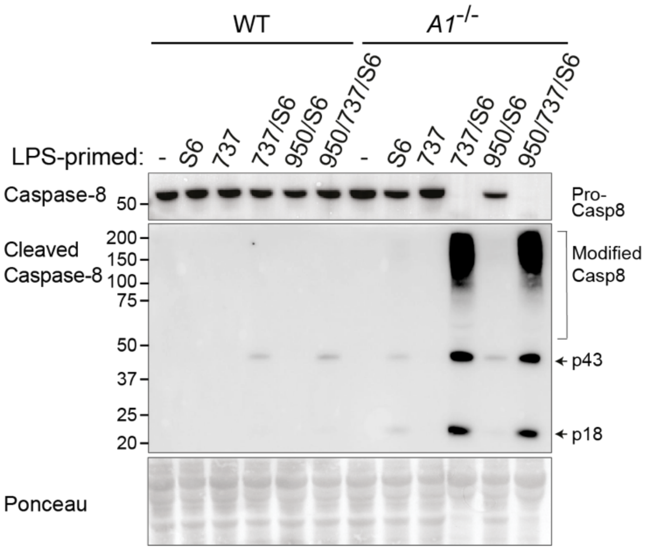

***Rebuttal Figure 1**. **BAX/BAK signalling promotes caspase-8 cleavage and modification that are associated with its activity**. WT and A1-deficient BMDMs were primed for 3 hours with LPS (50 ng/mL), treated in the last 30 minutes of priming with MCC950 (950; 5 µM) as indicated, and treated with ABT-737 (737; 0.5 µM) and S63845 (S6; 10 µM) for 6 hours. Immunoblots were performed on cellular lysates.*

R#1. - *with respect to deciphering residual necroptosis, others have shown that necroptosis can be engaged following MOMO dependent on TNF synthesis (see PMID: 28846096), is TNF required for necroptosis observed in the current study?*

Response 5. We thank the reviewer for asking this interesting question surrounding the role of MOMP-induced TNF in triggering necroptosis in our study. We have addressed this question in both LPS-primed and unprimed BMDMs (that better allow us to see evidence of MOMP-associated TNF secretion). We now show that in the absence of caspases in unprimed WT and A1-deficient BMDMs, activation of BAX/BAK specifically using S63845 and ABT-737 promotes TNF secretion and TNF-dependent necroptosis in a proportion of cells (**NEW DATA Fig. EV3A, EV3C, EV3D and EV3E, reproduced below for your convenience**), paralleling work by Giampazolias *et al.* in non-hematopoietic cells (Giampazolias E *et al.* 2017 *Nat Cell Biol* 19:1116 ref #49). We also show that Q-VD-OPh/S63845/ABT-737 treatment in LPS-primed WT and A1-deficient macrophages does at least partly induce TNF-dependent RIPK3 kinase-dependent necroptosis (RIPK3 kinase inhibitor GSK'872 delays cell death; **NEW DATA Fig. 3A, 3B, 3F and Fig. EV3F, see below**). This is in contrast to our data relating to ABT-737 and CHX treatment where BAX/BAK-dependent cell death is more efficiently inhibited by Q-VD-OPh (**Fig. 3A and EV3A of revised manuscript, reproduced below for your convenience**; Vince JE *et al.* 2018 *Cell Rep* 25:2339 ref#28), which we point out in the main text is likely to be due to CHX impairing synthesis of death complex regulators and promoting a pro-apoptotic complex. We also provide new data showing that when caspase activity is compromised, specific activation of BAX/BAK induces TNF and RIPK3-MLKL-mediated activation of the NLRP3 inflammasome (**NEW DATA Fig. 3C, 3D, 3E and 3F, see below**), as reported for extrinsic signalling (Conos SA *et al.* 2017 *PNAS* 114:E961 ref#29). Overall, our data suggest that, when BAX/BAK are specifically activated in LPS-primed cells using ABT-737 and S63845, in the presence of caspase inhibition, cells can be sensitised to TNF-driven necroptosis and NLRP3 inflammasome activation, and, importantly we show these events are delayed by A1 expression (lines 217-232, 239-262).

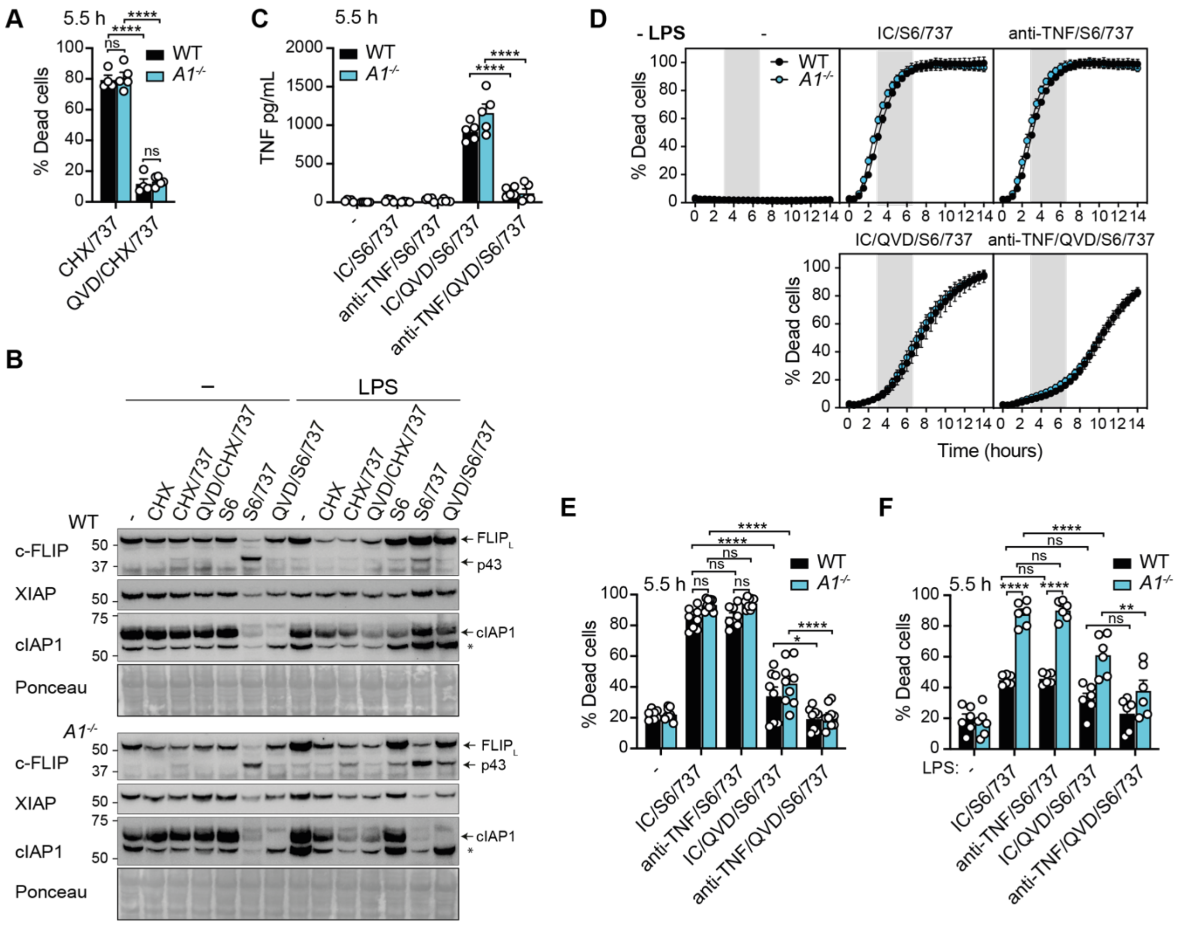

Figure EV3. In the presence of caspase inhibition, specific activation of BAX/BAK using S63845 and ABT-737 triggers TNF-dependent necroptosis.

(A, B) *WT and A1-deficient (A1$^{-/-}$) BMDMs were primed, as indicated, with B5 LPS (50 ng/mL) for 3 h, pre-treated with the pan-caspase inhibitor Q-VD-OPh (QVD; 40 µM) and/or GSK'872 (872; 1 µM) for the final 20-30 min of priming, as indicated, prior to the addition of S63845 (S6; 10 µM), ABT-737 (737; 500 nM), CHX (20 µg/mL), and z-VAD-fmk (zVAD; 50 µM), as specified, for up to 6 h.*

(A) *Cell death was measured via flow cytometric analysis of PI uptake. Data are presented as the mean + SEM, n = 4-5 mice/group pooled from 2 experiments. (NEW DATA)*

(B) *Cell lysates were analysed by immunoblot for the indicated proteins after 2 h. *cFLIP re-probe. One of two representative blots. Supplementary Figure 2M of original submission.*

(C-E) *WT and A1-deficient (A1$^{-/-}$) BMDMs were treated with 20 µg/mL isotype control (IC; GL113) or anti-TNF monoclonal antibody (XT22), as indicated. Cells were next incubated with Q-VD-OPh (QVD; 40 µM), as specified, and treated with S63845 (S6; 10 µM) and ABT-737 (737; 0.5 µM).*

(C) TNF secretion was measured in the culture supernatants by ELISA. Data are presented as the mean + SEM, n = 5 mice/group pooled from 2 experiments. (NEW DATA)

(D) Cell death was measured by PI uptake and time-lapse IncuCyte imaging. Data are presented as the mean ± SEM, n = 5-6 mice/group pooled from 2 experiments. (NEW DATA)

(E) Cell death was measured by PI uptake and flow cytometric analysis after 5.5 h. Data are presented as the mean + SEM, n = 8-9 mice/group pooled from 3 experiments. (NEW DATA)

(F) WT and A1-deficient (A1$^{-/-}$) BMDMs were treated with 20 µg/mL isotype control (IC; GL113) or anti-TNF monoclonal antibody (XT22), as indicated. Cells were next treated with LPS (50 ng/mL) for 3 hours, incubated with Q-VD-OPh (QVD; 40 µM) for the last 30 minutes of priming, as specified, and treated with S63845 (S6; 10 µM) and ABT-737 (737; 0.5 µM) for 5.5 h. Cell death was measured by PI uptake and flow cytometric analysis. Data are presented as the mean + SEM, n = 6 mice/group pooled from 2 experiments. (NEW DATA)

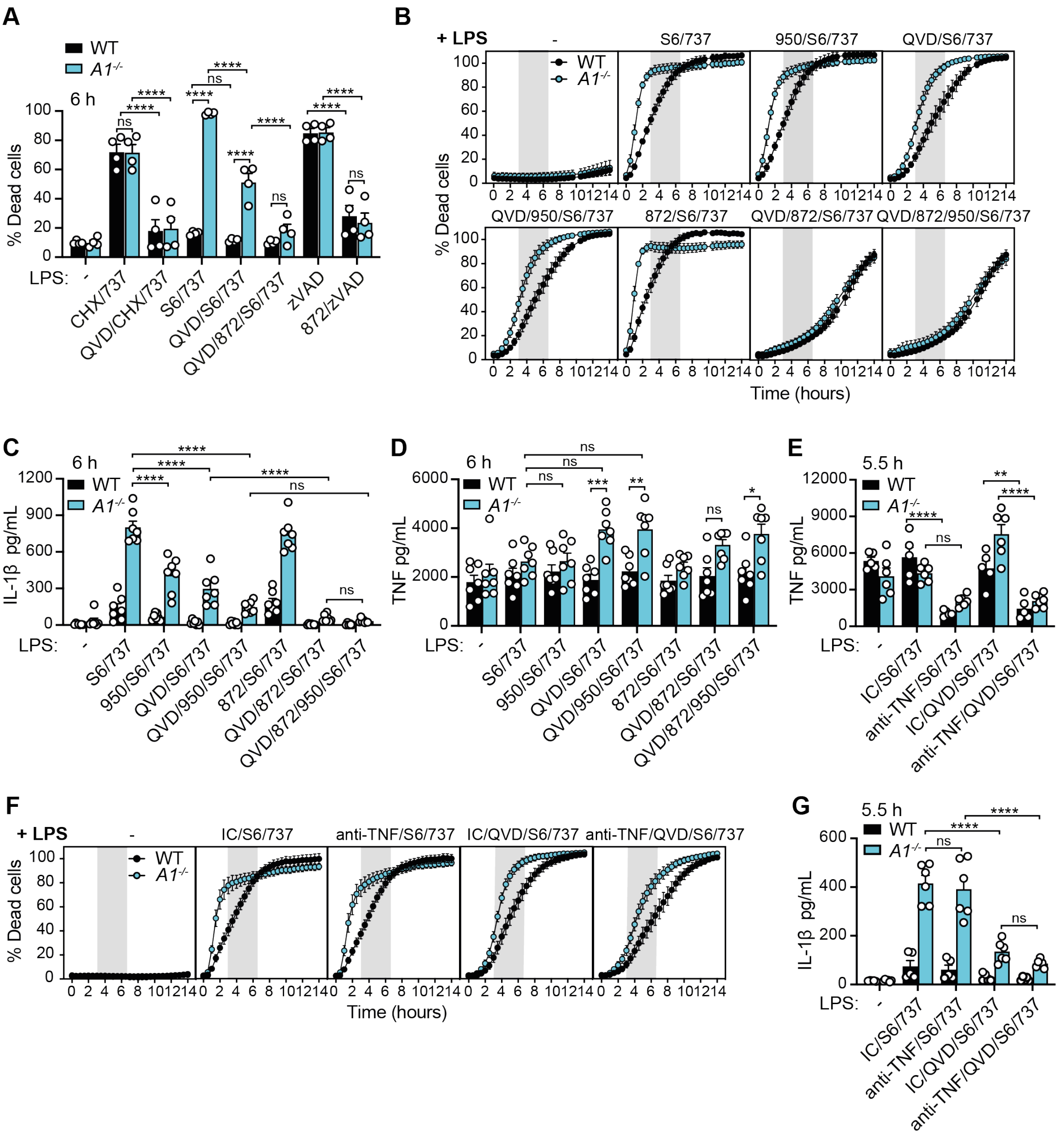

Figure 3. A1 delays BAX/BAK-mediated necroptosis and NLRP3 inflammasome activation in LPS-primed macrophages.

(A-D) WT and A1-deficient BMDMs were primed with LPS for 3 h, then treated with Q-VD-OPh (QVD; 40 µM), MCC950 (950; 5 µM) and GSK'872 (872; 1 µM), as specified, in the last 30 minutes of priming. Cells were then treated with z-VAD-fmk (zVAD 50 µM), cycloheximide (CHX 20 µg/mL), ABT-737 (727 0.5 µM) and S63845 (10 µM), as indicated.

(A) Cell death was measured by PI uptake and flow cytometric analysis. Data are presented as the mean + SEM, n = 4 mice/group pooled from 2 experiments.

(B) Cell death was measured by PI uptake and IncuCyte time-lapse imaging. Data are presented as the mean ± SEM, n = 5-6 mice/group pooled from 2 experiments.

(C) IL-1β and (D) TNF secretion was measured by ELISA. Data are presented as the mean + SEM, n = 7 mice/group pooled from 2 experiments

(E-G) WT and A1-deficient BMDMs were treated with 20 µg/mL isotype control (IC; GL113) or anti-TNF monoclonal antibody (XT22), as indicated. Cells were next treated with LPS (50 ng/mL) for 3 hours, incubated with Q-VD-OPh (QVD; 40 µM) for the last 30 minutes of priming, as specified, and treated with S63845 (S6; 10 µM) and ABT-737 (737; 0.5 µM).

(E) TNF and (G) IL-1β secretion was measured by ELISA. Data are presented as the mean + SEM, n = 5-6 mice/group pooled from 2 experiments.
(F) Cell death was measured by PI uptake and IncuCyte time-lapse imaging. Data are presented as the mean ± SEM, n = 6 mice/group pooled from 2 experiments.

Referee #2:

R#2. *Here the authors show that LPS-mediated upregulation of A1 modestly influences LPS-mediated death of macrophages (BMDMs) and monocytes, but only upon concurrent neutralization of Mcl-1 and Bcl-x (macrophages) or Mcl-1 (Monocytes) using BH3 mimetics. The role of A1 in regulating LPS-induced survival (and IL-1beta production downstream) on its own was negligible. The data, as presented, are sound and well interpreted. However, I don't feel that these observations rise to the level of significance that would warrant publication in the EMBO Journal for a number of reasons.* **6.1** *It is well known that pro-inflammatory stimuli, such as LPS and TNF, upregulate A1 (e.g. Hu et al., 1998 Blood 92, 2759). Loss of A1 by itself appears to play only a very minor role in regulating cell death and inflammasome activation downstream of LPS, with Mcl-1 and Bcl-xL playing much more dominant roles in this context (as evidenced by the robust increases in cell death seen upon treatment with S6/ABT737 in combination). The effects of A1 loss are only revealed when Mcl-1 and Bcl-xl are neutralized using synthetic BH3-mimetics, or in combination with protein synthesis blockade. I'm not persuaded that this represents any particular physiological scenario.*

6.2 *The effects of A1 loss were somewhat more significant when inflammation was driven with very high concentration of NOMVs (50 ug per mL), that effectively block protein synthesis, but it is not at all surprising that loss of a pro-survival Bcl-2 family member leads to accelerated cell death under certain conditions. Therefore, although the study is nicely conducted and the data are well described and presented, the findings reported herein do not have the novelty or significance that would warrant publication in the EMBO Journal and are more suited to publication in a specialist journal.*

Response 6.1. We firstly wish to thank the reviewer for their comment on the technical rigour of our study. In regard to the reviewer's suggestion that A1 itself does not appear to have a primary role in myeloid cell survival on its own, or in response to LPS, we totally agree. However, A1 may play a vital role in dictating when cell death occurs upon pathogen exposure, which will be of particular interest as the field continues to uncover pathogen effectors that can directly, or indirectly, target mitochondria, as well as target related BCL-2 family members, to manipulate host cell viability [Speir M *et al.* 2016 *Nat Microb* 1:15034 ref#8; Suzuki T *et al.* 2018 *PLOS Pathog* 14:e1007299 ref#18; Ohmer M *et al.* 2016 Cell Death Dis. 7:e2340 ref#20; Chauhan D *et al.* 2018 *Cell Rep* 25:2354 ref#32; Deo P *et al.* 2020 *Nat Microb* 5:1418 ref#33; Orzalli MH *et al.* 2021 *Immunity* 54:1447 ref#34; Kepp O *et al.* 2007 *EMBO J* 26:825 PMID:17235284]. As also astutely noted by the reviewer, inflammatory PAMPs or cytokines associated with infection induce A1 expression, yet, the effect of transient A1 expression on infections, particularly those that manipulate intrinsic cell death signalling, has not been studied until now. Also, with the recent discovery that intrinsic apoptosis can activate the NLRP3 inflammasome- and caspase-8-mediated IL-1β activation in LPS-primed macrophages [Vince JE *et al.* 2018 *Cell Rep* 25:2339 ref#28], elucidating the role of A1 in regulating apoptotic cell death and inflammation during infection has wide-reaching implications for our understanding of dynamic host-pathogen interactions.

With regard to the reviewer's opinion that these events (modelled using BH3-mimetics) are not particularly physiological, we would instead point to a number of published papers that we have now highlighted within the discussion (lines 476-481) that show that diverse pathogens target anti-apoptotic BCL-2 proteins, either directly or indirectly [Speir M *et al.* 2016 *Nat Microb* 1:15034 ref#8; Suzuki T *et al.* 2018 *PLOS Pathog* 14:e1007299 ref#18; Chauhan D *et al.* 2018 *Cell Rep* 25:2354 ref#32; Deo P *et al.* 2020 *Nat Microb* 5:1418 ref#33; Orzalli MH *et al.* 2021 *Immunity* 54:1447 #34]. This literature likely represents the tip of the iceberg with regard to the modulation of intrinsic apoptotic factors during infection. In fact, a recent study suggested that changes in the susceptibility of lung epithelial cells to apoptosis with ageing may be determined by the levels of pro- and anti-apoptotic BCL-2 family

proteins, and can have an impact on infection severity and host mortality following SARS-CoV-2 infection [Inde *et al.* 2021 Science Advances 7:eabf8609 ref#19]. Beyond infection, our results may also have implications for clinical outcomes in immunocompromised anti-cancer patients where chemotherapeutics that activate BAX and BAK may incidentally trigger NLRP3 activity. In support of this notion, chemotherapeutics that activate BAX and BAK have also been linked, albeit indirectly, with NLRP3 activation (Sauter KAD *et al.* 2011 *Cancer Biology & Therapy* 2011 11:1008 PMID: 21464611; Westbom C *et al.* 2015 *Plos One* 10:e0145404 PMID 26689911; Ghiringelli F *et al.* 2009 *Nat Med* 15:1170 PMID: 19767732; Wei S *et al.* 2020 Cardiovasc Toxicol 20:507 PMID: 32607760).

Response 6.2. With regard to the reviewer's comment that a high concentration (50 µg/mL) of *Neisseria* outer membrane vesicles (NOMVs) was used in this study, we refer to other studies in the literature that use a similar dose of OMVs derived from Gram-negative bacteria, establishing that this is normal practice in the field [Deo P *et al.* 2018 *PLOS Pathog* 14:e1006945 ref#87; Turner L *et al.* 2018 *Front Immunol* 9:1466 PMID: 30013553; Bitto NJ *et al.* 2018 *Immunol Cell Biol* 96:1120 PMID: 30003588; Deo P *et al.* 2020 *Nat Micro* 5:1418 ref#33]. Whilst it would be potentially interesting to titrate the concentration of NOMVs required to alter cell death signalling responses *in vitro* in A1-deficient cells, this is beyond the scope of this study.

Referee #3:

To understand the crosstalk between cell death and pathogen-induced innate immune responses, in this study, Speir et al demonstrated that the pro-survival BCL-2 family member A1, induced by pathogen ligands limits BAX/BAK-dependent apoptosis and IL-1β release in macrophages and inflammatory Ly6Chi monocytes. They also show that the survival modulator A1 in inflammatory monocytes delays death modalities and IL-1β activation triggered by Neisseria gonorrhoeae-derived outer membrane vesicles (NOMVs), exemplified by heightened IL-1β secretion into serum in response to NOMV injection in A1-deficient mice, advancing the development of myeloid cell-targeted antimicrobial therapeutics. This mechanistic study demonstrating A1 in controlling the timing of cell death and IL-1β production upon infection is important but more details are needed to strengthen the manuscript and make it suitable for a general interest journal.

Major concerns:

R#3. *1. The whole study is based on the notion that "Loss of MCL-1 and BCL-XL (or BCL-2) activity in LPS-primed macrophages can trigger BAX/BAK-dependent inflammation associated with the loss of ripoptosome repressors XIAP and cIAP1, and through effector caspase-3/-7-mediated activation of caspase-8 to directly cleave IL-1β [23, 25, 63]" (Text line 335 to 339) and authors proposed A1 restricts NLRP3 inflammasome-dependent/independent IL-1β secretion via MCL-1-BAX/BAK-caspase 3/7 axis (Fig 5I). However, mitochondrial dysfunction coupled with MOMP can lead to inflammasome activation independent of caspase 8, caspase 3 or caspase 7. This should be addressed and discussed.*

https://www.embopress.org/doi/full/10.15252/embr.201949799

Response 7. We thank the reviewer for pointing out that we have not discussed the conflicting literature surrounding mitochondrial dysfunction and canonical NLRP3 inflammasome activation. Our past work, using our genetic mouse models, has challenged several models that suggest mitochondrial dysfunction is universally triggered by canonical NLRP3 stimuli to induce inflammasome assembly and activation (Allam R *et al.* 2014 *EMBO Rep* 15:982 ref#24, Vince JE *et al.* 2018 *Cell Rep* 25:2339 ref#28). These include reports that activation of the BAX/BAK pore or mitochondrial permeability transition pore (mPTP) is required for oxidized mtDNA release to activate NLRP3 (Shimada. K *et al.* 2012 *Immunity* 55:1370 ref#27, Xian H *et al.* 2022 *Immunity* 36:401 ref#26). It is worth highlighting that Shimada and colleagues themselves actually show that mtDNA dominantly triggers AIM2 rather than NLRP3 (Shimada K *et al.* ref#27), and that a recent report showed that depletion of mtDNA in macrophages does not impact canonical NLRP3 activity induced by ATP treatment (Dang EV *et al.*, *Cell* 2017 171:1057 ref#25). We now amend our introduction (lines 77-92) to briefly include discussion of the

relevant proposed models and reference key papers and reviews that scrutinise the literature (Lawlor KE *et al.* 2014 *Biochim Biophys Acta* 1840:1433 ref#22; Riley JS *et al.* 2014 *EMBO rep* 21:e49799 ref#23; Yabal M *et al*. 2019 *J Leukocy Biol* 105:377 ref#21). We also wish to point out to the reviewer that our **NEW DATA** show how specific activation of BAX/BAK, largely in the absence of caspase activity, can trigger both TNF production and necroptotic signalling that culminates in NLRP3 activity in A1-deficient BMDMs (**see response 5 NEW DATA Fig. 3 and Fig. EV3**).

R#3. *2. The authors show that A1 and MCL-1 are key survival proteins in monocytes in sensing LPS or OMV while macrophages rely on A1, BCL-XL and MCL-1to prevent cell death (Fig 2-4 and Fig 5I). The physiological significance underlying this discrepancy between different types of inflammatory myeloid cells in response to infection needs to be discussed and linked with the in vivo results in Fig 5.*

Response 8. Yes, we show that A1 and MCL-1 are key survival proteins in monocytes, while A1, BCL-XL and MCL-1 prevent death of macrophages. We have now discussed the rationale for this difference in BCL-2 protein requirements in infection and reflected on why we failed to see clear changes associated with enhanced IL-1β production (lines 465-476). Ideally, we would like to have analysed mice at later time-points, however, our A1-deficient mice typically reached the ethical endpoint and needed to be euthanised by 6 hours post-NOMV-injection. While beyond the scope of this study, in the future we plan to test various infectious models that may modulate BAX/BAK signalling.

R#3. *3. This study emphasized the control of A1 in "timing/delaying" cell death and IL-1β production. Therefore, kinetic studies of caspases activation and NLRP3 inflammasome dependent/independent IL-1β production are crucial to make the whole mechanistic process clear.*

For example, Fig 4H is confusing. According to Fig 4E, expression level of A1 protein in WT monocytes at 5 h after NOMV treatment was relatively low, why would the authors choose "5 h" as an optimal timepoint to compare IL-1β production between WT vs A1 KO monocytes in Fig 4H?

Response 9. To address this point, we have performed time course experiments looking at caspase activation and IL-1β production in LPS-primed WT and A1-deficient BMDMs treated with BH3-mimetics and MCC950 at 1.5, 3, 4.5, 6, 9 and 24 hours to capture when cell death, caspase activity and IL-1β release ensue in WT versus A1-deficient macrophages. Our **NEW DATA** (**Fig. 2A, 2B, 2C, 2E and 2F of the revised manuscript, see below**) and OLD DATA (**Fig. 2A-G now Fig. EV2A-G of revised paper**) show that, in the absence of A1, LPS-primed macrophages exhibit robust early caspase cleavage and activity, which is marked by the cleavage of known substrates (*i.e.*, caspase-1/8: IL-1β p17 and GSDMD p30; caspase-3: GSDMD p43 and p20, GSDME p35 and PARP) and elevated early caspase-3 activity (assessed using a DEVDase assay) that can be blocked by pan-apoptotic caspase-inhibition. Correlating with this early caspase activity we observe that the A1-deficient BMDMs die (within the first 2-3 hours of ABT-737 and S63845 treatment) and exhibit accelerated and exaggerated NLRP3 inflammasome and IL-1β activity.

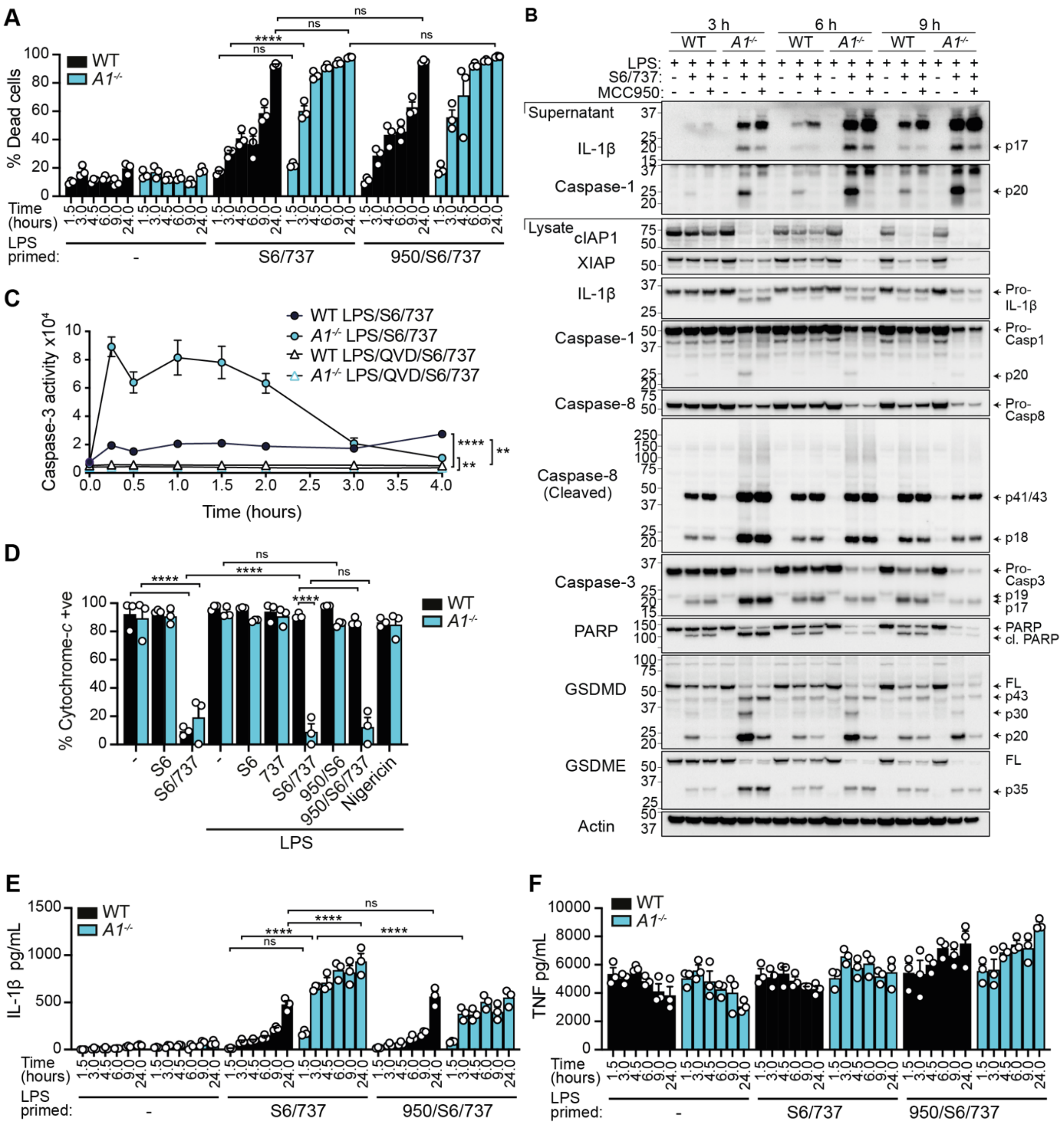

Figure 2. (NEW DATA) A1 deficiency leads to accelerated apoptotic cell death and inflammasome-dependent and -independent IL-1β activation in LPS-primed macrophages.

(A-F) WT and A1-deficient (A1$^{-/-}$) BMDMs were primed with B5 LPS (50 ng/mL) for 3 h, pre-treated with the NLRP3 inhibitor MCC950 (950; 5 µM) for the last 20-30 min of priming, as specified, prior to the addition of S63845 (S6; 10 µM) and/or ABT-737 (737; 500 nM), as indicated. In some cases, BMDMs were primed with LPS for 3 h and treated with nigericin (10 µM) for 1 h.

(A) Cell death was measured by PI uptake using IncuCyte time-lapse imaging. Data are presented as the mean ± SEM, n = 6 mice/group pooled from 2 experiments.

(B) Immunoblot analysis was performed on cell lysates and supernatants for relevant proteins over time. One representative blot of at two shown. Ponceau stain and β-actin were used as a control for the loading of total protein.

(C) WT and A1-deficient (A1$^{-/-}$) BMDMs were primed with B5 LPS (50 ng/mL) for 3 h, pre-treated with the pan-apoptotic caspase inhibitor Q-VD-Oph (QVD; 5 µM) for the last 20-30 min of priming, as specified, prior to the addition of S63845 (S6; 10 µM) and/or ABT-737 (737; 500 nM) for the indicated times. Caspase-3 activity was measured over time using a DEVDase assay. n = 3 mice/group and is representative of 1 of 2 experiments.

(D) Cytochrome-c retention, as a measure of MOMP and marker of intrinsic cell death, was assessed by flow cytometric analysis. Data are presented as the mean + SEM, n = 3 mice/group and are representative of 3 experiments.
(E) IL-1β and (F) TNF levels in the cell supernatant were measured by ELISA. Data are presented as the mean + SEM, n = 3 mice/group and are representative of at least 3 experiments.

With regard to the timing and kinetics of our NOMV responses shown in Fig. 4E and 4H (**now Fig. 5E and 5G in our revised manuscript**), we observe some variation between cells and NOMV batches as to when A1 levels decline. However, we optimally see cell death and IL-1β activation between 4-5 hours in our sorted monocytes (**revised manuscript Fig. 5F, 5G and 5I**), when both A1 and MCL-1 appear to decline (**revised manuscript Fig. 5E**) and sufficient NLRP3 inflammasome priming has occurred. Moreover, a 5-hour time-point allowed us to capture the switch to necroptosis and related IL-1β activation to Q-VD-OPh/NOMV treatment (**revised manuscript Fig. 5F, 5G and 5I**). For the reviewers benefit, we include data showing that at an earlier time-point (3 hours) we failed to see significant amounts of IL-1β, most likely due to insufficient time for inflammasome priming (**Rebuttal Figure 2, <u>y-axis scaled to match the 5-hour data in revised manuscript Fig. 5G</u>**).

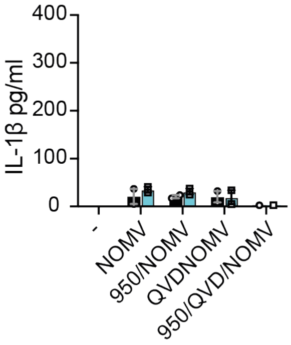

Rebuttal Figure 2. NOMVs fail to significantly induce inflammasome and IL-1β activation after 3 hours of treatment. *Bone marrow inflammatory monocytes were isolated and pre-treated with pan-apoptotic caspase inhibitor Q-VD-OPh (QVD; 40 μM) and/or NLRP3 inhibitor MCC950 (950; 5 μM), as indicated, for 30 minutes, prior to treatment with NOMVs (50 μg/mL) for a further 3 hours. Levels of IL-1β secreted into the supernatant were measured by ELISA. n = 2 mice, mean ± S.D.*

R#3. 4. *Are cell death and IL-1β production tightly linked/synchronized? In Fig 2B, cell death in S6/737 group between WT BMDMs and A1 KO BMDMs was similar, whereas in Fig 2E, IL-1β production was much more robust in A1 KO BMDMs, compared to WT BMDMs. Is IL-1β production here unrelated to caspase 8 or a "delayed" response? This should be discussed.*

Response 10. We do see inter-experiment variability in the amount of IL-1β detected in supernatants at 24 hours in WT BMDMs, which will be caused by differences in our culturing conditions (*e.g.*, L929 batches, BH3-mimetic batches) over a period of 3+ years (COVID-19 lockdowns delayed experiments). Most likely these differences cause variations in the magnitude and kinetics of LPS-induced A1 turnover between experiments (*i.e.*, A1 expression typically declines anywhere between 8-12 hours post-LPS treatment). The magnitude of IL-1β responses may also vary due to the differences in the expression of transcriptional and post-translational negative regulators of NLPR3 and IL-1β activity (*e.g.*, Yabal M et al. JLB 2019 105:377 ref# 21; Poudel B *et al.* JLB 2018 doi: 10.1002/JLB.3MIR0917-350R; Vijayaraj S *et al.* 2021 *Nat Commun* 12:2713 ref #38). However, it is important to mention that fitting with our conclusions that A1 limits cell death and inflammatory signalling, we consistently observe early cell death and IL-1β responses in A1-deficient cells in response to ABT-737 and S64845 compared to WT cells. We have now included new data sets performed in WT and A1-deficient BMDMs generated using consecutive batches of L929 to show the consistency of our cell death and IL-1β data and complement our existing data (**NEW DATA Fig. 1A-D, Fig. 2A, B, C, E, F, Fig. 3, Fig. EV1A-H and EV3A, C, D-F**). A discussion surrounding experimental variability has now been included in the results section (<u>lines 182-192</u>). It is also important to note that our use of an IncuCyte to perform detailed time-lapse imaging of cell death revealed that cell density can also impact the rate of cell death; with the low density required for imaging leading to accelerated cell death. This is a phenomenon we previously observed in our investigations into extrinsic cell death and inflammatory signalling (Lawlor KE et al. 2017 *Cell Reports* 20:668 ref#41). Overall,

this difference does not affect our conclusions, as A1 expression still delayed cell death at low and high cell densities.

Minor concerns:

R#3. *1. In addition to IL-1β production, how does caspase 8 modulate pyroptotic pore formation for IL-1β secretion? In Fig 4J and Fig S4F, the author show GSDMD p30 was elevated when apoptotic caspases were inhibited by QVD, suggesting apoptotic caspases limit GSDMD N-terminal p30 cleavage and pyroptosis. How does this link to IL-1β secretion? This should be addressed.*

Response 11. We have now amended the text to better clarify the data presented (lines 325-364). To summarise our findings, in response to NOMVs we dominantly observe apoptosis, as suggested by reduced cytochrome-*c* retention in the mitochondria (*i.e.*, intrinsic apoptosis) (**revised manuscript Fig. EV4D**) and delayed cell death with pan-apoptotic caspase inhibitor Q-VD-OPh (**revised manuscript Fig. 5F**). Inflammasome and GSDMD-mediated pyroptosis appears to play a negligible role in cellular demise under these conditions, as inhibiting NLRP3 with MCC950 alone, or together with Q-VD-OPh, failed to impact death kinetics to NOMV treatment (revised manuscript **Fig. 5F**; **see MCC950/NOMV and QVD/MCC950/NOMV versus NOMV and QVD/NOMV, respectively**). Correspondingly, as the reviewer suggests, and in line with our past findings with BAX/BAK signalling in TLR-primed macrophages (Vince JE *et al.* 2018 *Cell Rep* 25:2339 ref#28), we observe robust inactivation of GSDMD (p20 and p43 fragments **NEW DATA Fig 5I and EV4E of revised manuscript**) that is caused by apoptotic caspase-3. We do note that there is also evidence of GSDMD p30 in these lysates that is associated with cleaved caspase-1 and caspase-8. Hence, Q-VD-OPh-targeting of apoptotic caspases (caspase-3/7, caspase-8 and caspase-9) subsequently permits efficient NLRP3 inflammasome-mediated activation of GSDMD (namely, it is MCC950 blockable; **NEW DATA Fig 5I and EV4E; see QVD/NOMV vs QVD/MCC950/NOMV**). However, it is crucial to reiterate that in the absence of apoptotic caspase activity these cells do not die by pyroptosis but engage RIPK3-dependent necroptosis (**Fig. 5F revised manuscript; see QVD/NOMV vs QVD/950/NOMV vs QVD/872/NOMV**), which suggests that MLKL signalling (and pore formation) may act upstream of NLRP3 to promote IL-1β activation and secretion. However, impressively we now find that inhibition of necroptosis with GSK'872 fails to block GSDMD cleavage and IL-1β activation (**see Fig. 5I and EV4E QVD/872/NOMV compared to 950/QVD/NOMV**), suggesting that when apoptosis and necroptosis are inhibited NLRP3 activity is still triggered by an alternative mechanism (**NEW DATA Fig. 5I and EV4E, EV4F, EV4G, EV4H of revised manuscript**).

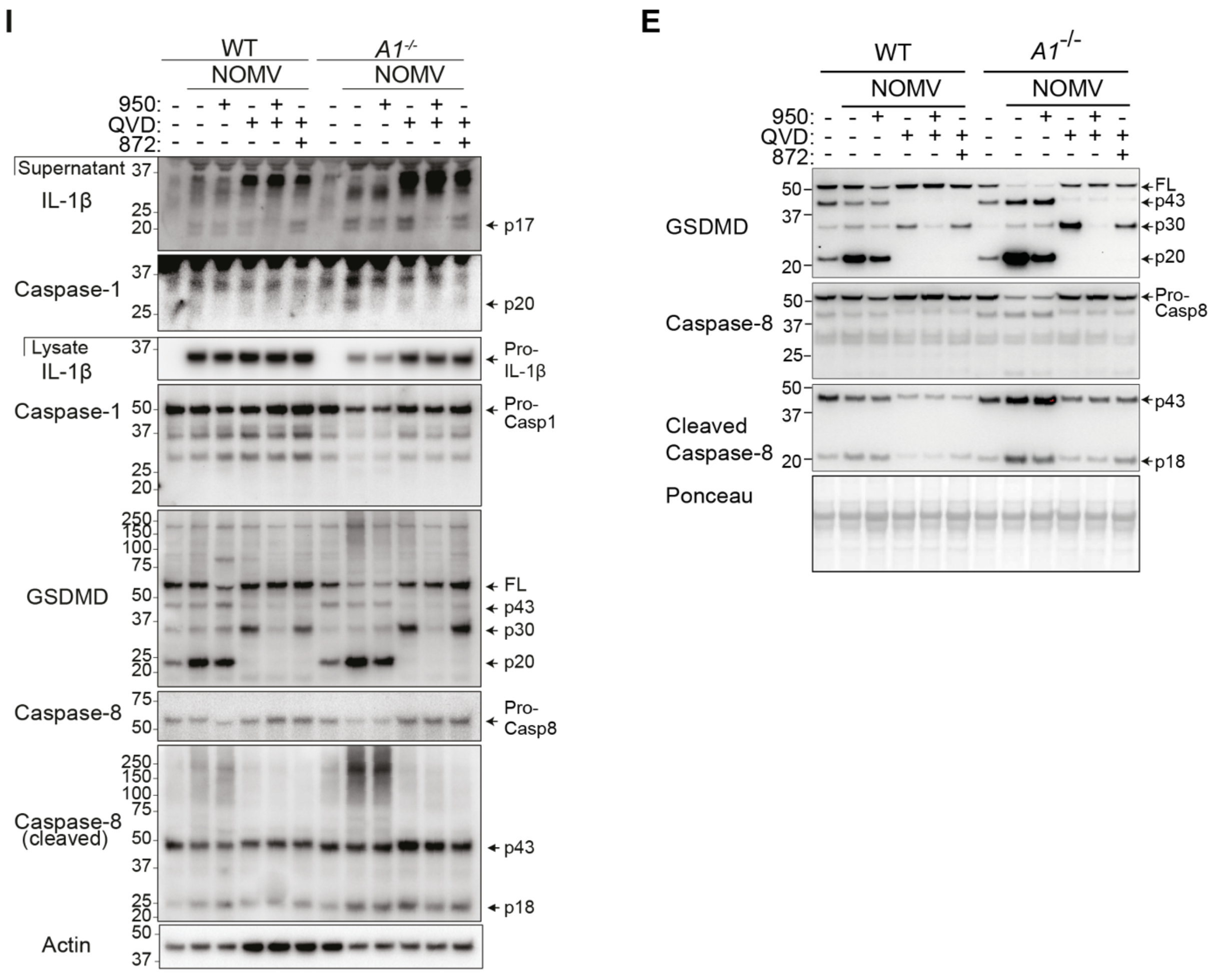

Figure 5I (NEW DATA) and Revised EV4F. *Bone marrow inflammatory monocytes were isolated and pre-treated with pan-apoptotic caspase inhibitor Q-VD-OPh (QVD; 40 μM), MCC950 (950; 5μM) and GSK'872 (872; 1 μM) as indicated, for 30 minutes, prior to treatment with NOMVs (50 μg/mL) for a further 5 hours. Immunoblots of cellular lysates and supernatants for relevant proteins.*

While we do not show a direct correlation between caspase-8 and pyroptotic pore formation and IL-1β secretion, residual GSDMD p30 expression in MCC950-treated cells is likely be caspase-8-mediated, as we now allude to in the text. Likewise, and as discussed above, apoptotic caspase-3 does inactivate GSDMD to limit pyroptosis. It is worth noting that it is unlikely that GSDMD pores play a major role in IL-1β secretion, as three recent studies have shown that BAX/BAK signalling largely bypasses the need for GSDMD, GSDME, and GSDMD/E for both cell death and IL-1β secretion in primary macrophages (Chen KW *et al.* 2019 *EMBO J* 38:e101 ref#55; Vince JE *et al.* 2018 *Cell Rep* 25:2339 ref#28, Hughes SA et al. 2023 *EMBO J* 42(5):e110468 PMID: 36647747). However, to address whether GSDMD pores are required for IL-1β secretion in response to NOMVs, we firstly examined NOMV-induced inflammatory responses in WT and A1-deficient Ly6C^{hi} monocytes pre-treated with the GSDMD inhibitor Disulfiram (Hu JJ *et al.* 2020 *Nature Immunol.* 21:736 PMID: 3237036) Surprisingly, while we observed a complete block in GSDMD cleavage we found that the NOMVs also failed to induce caspase-8 activation or precursor IL-1β expression (**Rebuttal Fig. 3**). These data suggest that disulfiram may impede sensing of the NOMVs or inhibit transcription via its antioxidant properties at the high 50 μM dose adopted (N.B. No obvious cytotoxicity was observed). Therefore, to avoid further optimisation steps, we next examined responses in WT and GSDMD-deficient inflammatory monocytes. As predicted, GSDMD pores were not required for IL-1β secretion in response to NOMVs during apoptotic and necroptotic signalling (**NEW DATA EV4F, EV4G, EV4H, reproduced below for your convenience**) but blockade of both modes of cell death (**with QVD/872**)

led to a GSDMD-dependent activation of NLRP3 and IL-1β secretion (**NEW DATA EV4F**). We believe this is likely driven by noncanonical caspase-11 activity triggered by LPS within the NOMVs. We show preliminary evidence for caspase-11 cleavage in 872/QVD/NOMV-treated WT and *Gsdmd*$^{-/-}$ monocytes (**Rebuttal Fig. 4**) but have elected not to include these data in the paper as these experiments have been difficult and inconclusive (*i.e.*, caspase-11 has proven difficult to consistently detect in small numbers of isolated monocytes) and, ultimately, these findings are not relevant to the core message of the paper that A1 can be induced by pathogen molecules to limit apoptotic cell death.

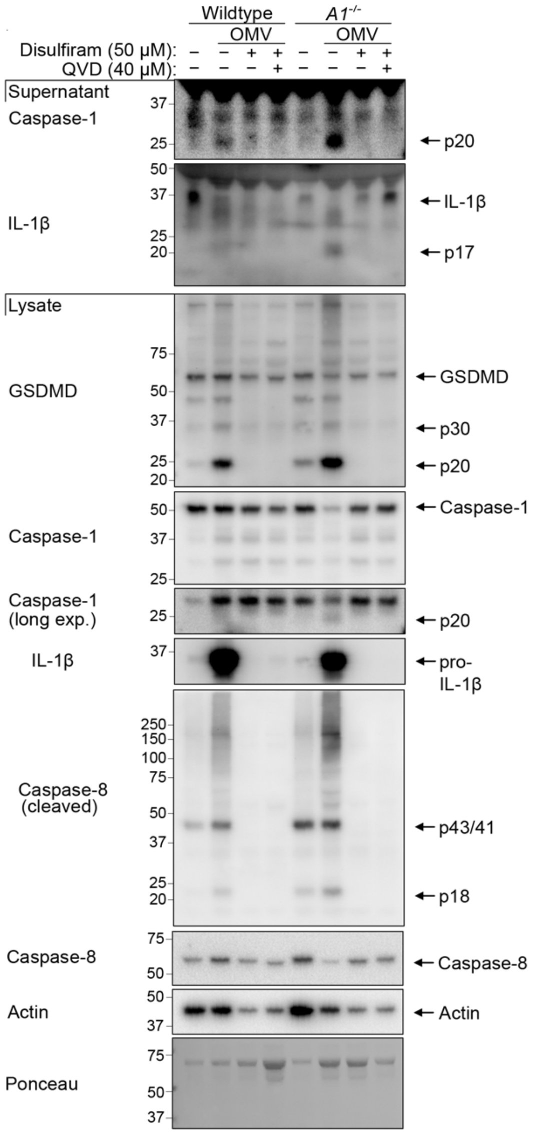

Rebuttal Figure 3. GSDMD inhibitor Disulfiram prevents NOMV-induced caspase and pro-IL-1β induction. *Bone marrow inflammatory monocytes were isolated and pre-treated with pan-apoptotic caspase inhibitor Q-VD-OPh (QVD; 40 µM) and/or GSDMD inhibitor Disulfiram (50 µM), as indicated, for 30 minutes, prior to treatment with NOMVs (50 µg/mL) for a further 5 hours. Immunoblots of cellular lysates and supernatants for relevant proteins.*

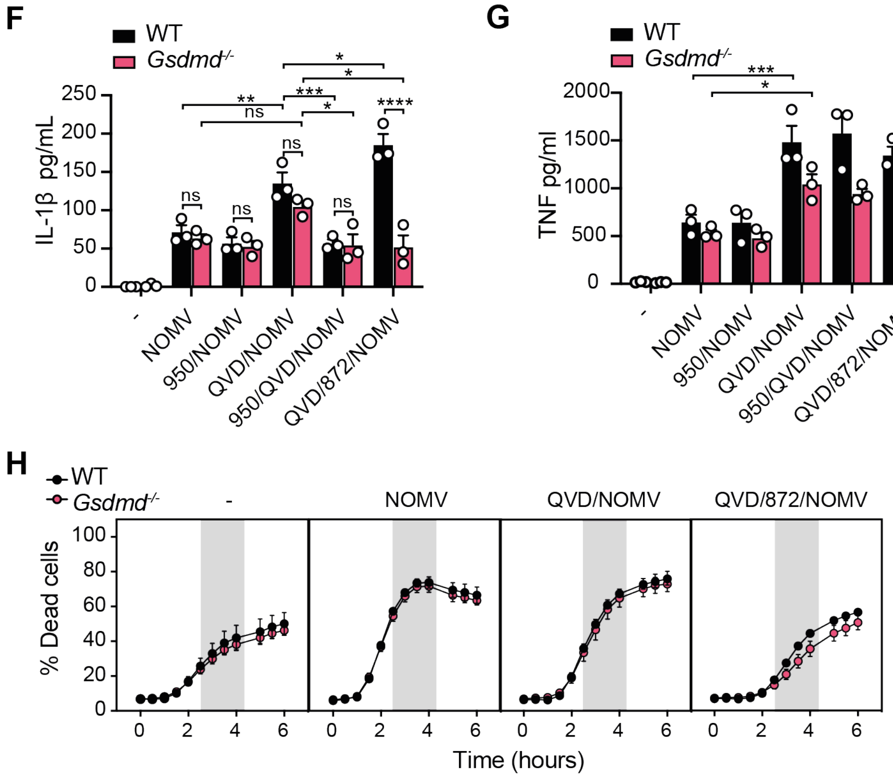

NEW DATA EV4. GSDMD pores are not absolutely required for IL-1β secretion upon exposure to NOMVs. *WT and GSDMD-deficient Ly6Chi monocytes were treated with QVD (40 µM) and GSK'872 (1 µM), as specified, for 30 minutes prior to treatment with NOMVs (50 µg/mL). After 6 hours (F) IL-1β and (G) TNF secretion was measured by ELISA. (H) Cell Death was measured by PI uptake and IncuCyte time-lapse imaging. Data are the mean ± SEM. (A, B D) n = 3 mice per group.*

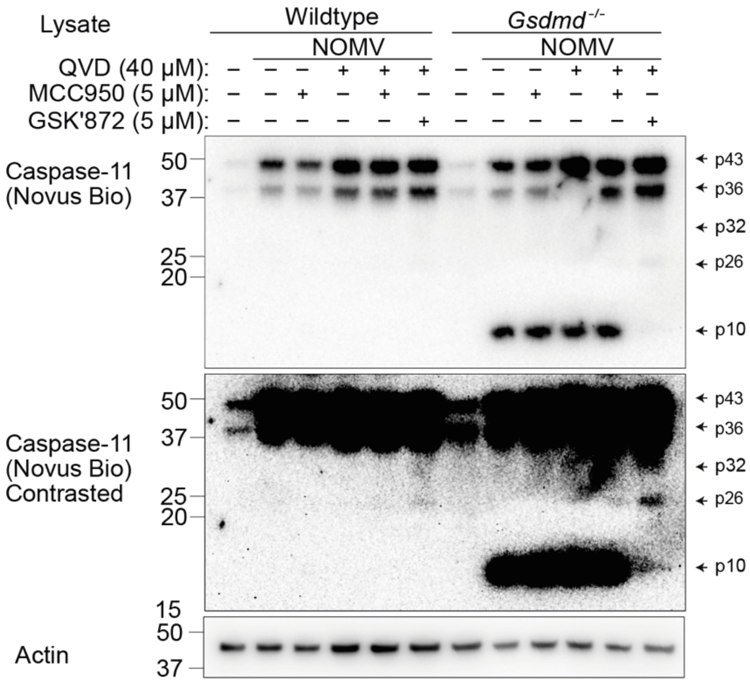

Rebuttal Fig. 4. Blockade of apoptosis and necroptosis in NOMV-treated A1-deficient Ly6Chi monocytes induces caspase-11 cleavage. *WT and A1-deficient Ly6Chi monocytes were treated with QVD (40 µM) and GSK'872 (1 µM), as specified, for 30 minutes prior to treatment with NOMVs (50 µg/mL). After 5 hours cell lysates were interrogated for caspase-11 cleavage by immunoblot.*

R#3. *2. The PI uptake measured throughout the whole manuscript to demonstrate "apoptosis" may need to be revised. This method would indicate diverse types of cell death including necroptosis and pyroptosis.*

Response 12. While we agree the PI staining does not differentiate between different types of cell death, our previous study [Vince JE *et al*. 2018 *Cell Rep* 25:2339 ref#28] showed genetically that cell death induced upon BCL-XL and MCL-1 targeting is BAX/BAK- and caspase-3/7-dependent (*i.e.*, intrinsic apoptosis) in both LPS-primed and unprimed cells. Moreover, we showed genetically that macrophage cell death was independent of extrinsic (RIPK3/caspase-8) and pyroptotic cell death (NLRP3, caspase-1/11, GSDMD, GSDME) machinery. In this study, to confirm BAX/BAK activation we measured loss of mitochondrial cytochrome-*c* retention to mark commitment to intrinsic cell death (**Fig. 2D, 4C, EV4D, Appendix Fig. S1, Appendix Fig. S2, Appendix Fig. S4** of the revised manuscript). We also used chemical inhibitors, in parallel with BH3-mimetics, or NOMVs, to demonstrate that cell death in macrophages and monocytes is not pyroptotic in nature *i.e.*, death is not MCC950 blockable **(Fig. 2A, 3B, 4A and EV2A, EV2B, EV4B, and EV4E**) and instead is largely dependent on apoptotic caspase activity *i.e.*, Q-VD-OPh delays cell death (**Fig. 3A, 3B, 3F, 5F, 5G, EV3F and EV4H)**. Importantly, we now also demonstrate that highly specific triggers of BAX/BAK activation and MOMP can trigger RIPK3 kinase-dependent necroptosis in the absence of caspase activity *i.e.*, RIPK3 inhibitor GSK'872 further delayed cell death in Q-VD-OPh/ABT-737/S63845 and Q-VD-OPh/NOMV treated cells (**Fig. 3A, NEW DATA 3B, 5F, 5G, EV4H, see response 5**). To further improve our manuscript, we have now performed additional experiments in an attempt to further confirm that apoptotic cell death is being triggered. We firstly performed time-lapse imaging experiments using a caspase-3/7 activity probe in parallel with experiments examining PI uptake (**NEW DATA Figure 3B and Rebuttal Fig. 5**). We now show that apoptotic caspase-3/7 activity is indeed reduced in LPS-primed A1-deficient (and WT) BMDMs treated with ABT-737/S63845 upon pre-incubation with pan-apoptotic caspase inhibitor QVD. However, surprisingly, we find that this probe can be non-specific as it is also reduced when GSK'872 is used to block necroptotic cell death to Q-VD-OPh/ABT-737/S63845 treatment (**Rebuttal Fig. 5**). We next performed DEVDase assays to confirm that apoptotic caspase-3 activity was rapidly induced and proceeded cell death in A1-deficient BMDMs upon LPS/ABT-737/S63845 treatment (**NEW DATA Fig. 2C of revised manuscript, <u>see response 9</u>**).

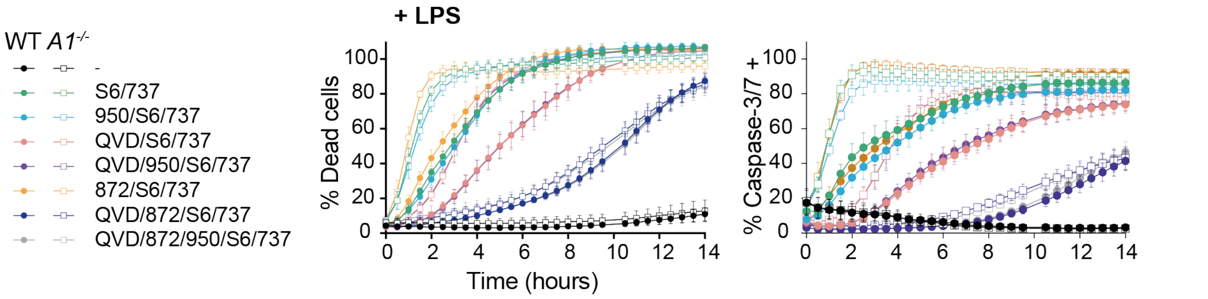

Rebuttal Fig. 5. Analysis of apoptotic activity using a caspase-3/7 activity probe. *WT and A1-deficient BMDMs were primed with LPS (50 ng/mL) for 3 hours and were treated with QVD (40 μM), MCC950 (5 μM) and GSK'872 (1 μM), as specified, for 30 minutes prior to treatment with ABT-737 (0.5 μM) and S63845 (10 μM). Cells were then incubated with membrane labelling dye and either PI or the caspase-3/7 activity probe and imaged for 14 hours on an IncuCyte. Data are the mean ± SEM. n = 6 mice from 2 pooled experiments.*

R#3. *3. Why was IL-1β production increase in A1 KO mice only observed in serum samples (Fig 5) but not peritoneal lavage fluid (Fig S5) when the acute inflammatory responses were assessed after 6 h of intraperitoneal injection of NOMV. This should be discussed.*

Response 13. We are not sure why we did not see a difference in local peritoneal responses at this time-point, although we hypothesise that local resident macrophages may exhibit delayed apoptotic and inflammatory responses (Deo P *et al.* 2020 *Nat Micro* 5:1418 ref#33) and refer to that in the text (<u>lines</u>

467-473). Unfortunately, our A1-deficient mice routinely reached our ethical endpoint at 6 hours post OMV injection preventing us from examining later time-points.

R#3. 14.1 *4. The accuracy of description needs to be improved. For example, in Fig 2 C and Fig 3C, it is not precise to state "cytochrome-c release were assessed by flow cytometric analysis as a measure of cell death and MOMP, respectively.". What the authors show in the figure was remaining cyto-c rather than released cyto c signal.*

14.2 *It is not accurate to state "Crucially, IL-1β secretion was almost completely abrogated by combined pre-treatment with MCC950 and Q-VD-OPh (Fig. 4H, 4J), confirming that caspase-1 and caspase-8 were both required for full IL-1β activity." (Line 286-288). Q-VD-OPh is pan-caspase inhibitor suppressing several caspases in addition to caspase 8.*

Response 14.1. We have amended the text in the figure legends to state cytochrome-*c* "retention" rather than "release". We based our wording on an original description of the assay [see Waterhouse NJ and Trapani JA (2003) A new quantitative assay for cytochrome-*c* release in apoptotic cells. *Cell Death Differ.* ***10***: 853-855 ref#91].

Response 14.2. With regard to the reviewer's second point, as far as we are aware no other caspases beyond caspase-1 and caspase-8 have been implicated in robust IL-1β processing to its active fragment in mouse cells, including caspase-9 (Conos SA *et al*. 2016 *Cell Death Differ* 23:1827 PMID: 27419363) and caspase-11 (Exconde PM *et al.* BiorXiv doi.org/10.1101/2023.02.16.528859). Therefore, based on our detection of caspase-1 and caspase-8 cleavage and the switch to necroptosis we observed with pan-apoptotic caspase inhibition (which suggests that caspase-8 activity limits RIPK3 signalling in this setting), as well as the fact we genetically showed that BAX/BAK signalling activates caspase-8 and IL-1β in LPS-primed BMDMs (ref #28) we feel confident in concluding that caspase-1 and caspase-8 act in parallel downstream of BAX and BAK to trigger IL-1β activation to NOMVs. Although, we now add to our findings with NOMVs by showing that in the presence of QVD and QVD/872 that necroptotic and pyroptotic signalling, respectively, can trigger NLRP3 activity.

R#3. *5. Data inconsistency needs to be addressed. In Fig S2B and Fig S2D, IL-1β was reduced by A1 loss compared to WT.*

Response 15. See Response 10 above.

Dear Dr. Lawlor,

Thank you for the submission of your revised manuscript to our editorial offices. I have now received the reports from two of the three referees that I asked to re-evaluate your study, you will find below. Referee #3 declined my invitation and refused to assess the revision. However, going through your p-b-p-response, I consider her/his points as adequately addressed. As you will see, the remaining referees are satisfied by the revision and support publication of the manuscript in EMBO reports.

Before proceeding with formal acceptance, I have these editorial requests I ask you to address in a final revised manuscript:

- Please add up to 5 keywords to the manuscript text, below the abstract.

- Please provide individual production quality figure files as .eps, .tif, .jpg (one file per figure) also for the 5 EV figures. Please upload these as separate, individual files upon re-submission. The figure legend for these should be included in the main manuscript document file in a section called Expanded View Figure Legends after the main Figure Legends section.

- We now use CRediT to specify the contributions of each author in the journal submission system. CRediT replaces the author contribution section. Please use the free text box to provide more detailed descriptions and do not provide your final manuscript text file with an author contributions section. See also our guide to authors:
https://www.embopress.org/page/journal/14693178/authorguide#authorshipguidelines

- We updated our journal's competing interests policy in January 2022 and request authors to consider both actual and perceived competing interests. Please review the policy https://www.embopress.org/competing-interests and update your competing interests if necessary. Please name this section 'Disclosure and Competing Interests Statement' and put it after the Acknowledgements section.

- Please add the manuscript number to the author checklist and update the Journal.

- Regarding data quantification and statistics, please make sure that the number "n" for how many independent experiments were performed, their nature (biological versus technical replicates), the bars and error bars (e.g. SEM, SD) and the test used to calculate p-values is indicated in the respective figure legends (main, EV and Appendix figures). Please also check that all the p-values are explained in the legend, and that these fit to those shown in the figure. Please provide statistical testing where applicable. Please avoid the phrase 'independent experiment', but clearly state if these were biological or technical replicates. Please also indicate (e.g. with n.s.) if testing was performed, but the differences are not significant. In case n=2 please show the data as separate datapoints or bars without error bars and statistics. See also:
http://www.embopress.org/page/journal/14693178/authorguide#statisticalanalysis

If n<5, please show single datapoints for diagrams.

- Please make sure that all figure panels are called out separately and sequentially. Presently, there seems to be no separate callouts for Fig 6C. Please check.

- Please change the callouts for the Appendix figures. These should follow the nomenclature Appendix Figure Sx.

- Please provide the final manuscript text file without the referee token in the Data Availability Section and make sure that the dataset is public latest upon publication of the manuscript.

- Please format the references according to our journal style:
http://www.embopress.org/page/journal/14693178/authorguide#referencesformat

- Please remove the sentence 'Source data are available online for this figure' from the legends. SD will be linked to each figure online.

- Finally, please find also attached a word file of the manuscript text (provided by our publisher) with changes we ask you to include in your final manuscript text and comments. Please use the attached file as basis for further revisions and provide your final manuscript file with track changes, in order that we can see any modifications done.

In addition, I would need from you:
- a short, two-sentence summary of the manuscript (not more than 35 words).
- two to four short (!) bullet points highlighting the key findings of your study (two lines each).
- a schematic summary figure as separate file that provides a sketch of the major findings (not a data image) in jpeg or tiff format (with the exact width of 550 pixels and a height of not more than 400 pixels) that can be used as a visual synopsis on our website.

I look forward to seeing the final revised version of your manuscript when it is ready. Please let me know if you have questions regarding the revision.

Best,

Achim Breiling
Senior Editor
EMBO Reports

Referee #1:

I appreciate the authors' efforts in comprehensively addressing all comments I raised.

Referee #2:

The authors have responded satisfactorily to my previous comments and have made modifications to the text to support their arguments. I have no further comments.

The authors have addressed all minor editorial requests.

Dr. Kate Lawlor
Hudson Institute of Medical Research
Centre for Innate Immunity and Infectious Disease
27-31 Wright Street
Clayton, Victoria 3165
AUSTRALIA

Dear Dr. Lawlor,

I am very pleased to accept your manuscript for publication in the next available issue of EMBO reports. Thank you for your contribution to our journal.

At the end of this email I include important information about how to proceed. Please ensure that you take the time to read the information and complete and return the necessary forms to allow us to publish your manuscript as quickly as possible.

As part of the EMBO publication's Transparent Editorial Process, EMBO reports publishes online a Review Process File to accompany accepted manuscripts. As you are aware, this File will be published in conjunction with your paper and will include the referee reports, your point-by-point response and all pertinent correspondence relating to the manuscript.

If you do NOT want this File to be published, please inform the editorial office within 2 days, if you have not done so already, otherwise the File will be published by default [contact: emboreports@embo.org]. If you do opt out, the Review Process File link will point to the following statement: "No Review Process File is available with this article, as the authors have chosen not to make the review process public in this case."

Thank you again for your contribution to EMBO reports and congratulations on a successful publication. Please consider us again in the future for your most exciting work.

Yours sincerely,

Achim Breiling
Editor
EMBO Reports

****

THINGS TO DO NOW:

Please note that you will be contacted by Wiley Author Services to complete licensing and payment information. The required 'Page Charges Authorization Form' is available here: https://www.embopress.org/pb-assets/embo-site/er_apc.pdf - please download and complete the form and return to embopressproduction@wiley.com

EMBO Press participates in many Publish and Read agreements that allow authors to publish Open Access with reduced/no publication charges. Check your eligibility: https://authorservices.wiley.com/author-resources/Journal-Authors/open-access/affiliation-policies-payments/index.html

You will receive proofs by e-mail approximately 2-3 weeks after all relevant files have been sent to our Production Office; you should return your corrections within 2 days of receiving the proofs.

Please inform us if there is likely to be any difficulty in reaching you at the above address at that time. Failure to meet our deadlines may result in a delay of publication, or publication without your corrections.

All further communications concerning your paper should quote reference number EMBOR-2023-56865V3 and be addressed to emboreports@wiley.com.

Should you be planning a Press Release on your article, please get in contact with emboreports@wiley.com as early as possible, in order to coordinate publication and release dates.

EMBO Press Author Checklist

Corresponding Author Name: KE Lawlor	
Journal Submitted to: EMBO Reports	
Manuscript Number: EMBOR-2023-56865V3	

USEFUL LINKS FOR COMPLETING THIS FORM
The EMBO Journal - Author Guidelines
EMBO Reports - Author Guidelines
Molecular Systems Biology - Author Guidelines
EMBO Molecular Medicine - Author Guidelines

Reporting Checklist for Life Science Articles (updated January
This checklist is adapted from Materials Design Analysis Reporting (MDAR) Checklist for Authors. MDAR establishes a minimum set of requirements in transparent reporting in the life sciences (see Statement of Task: 10.31222/osf.io/9sm4x). Please follow the journal's guidelines in preparing your
Please note that a copy of this checklist will be published alongside your article.

Abridged guidelines for figures
1. Data
The data shown in figures should satisfy the following conditions:
- the data were obtained and processed according to the field's best practice and are presented to reflect the results of the experiments in an accurate and unbiased manner.
- ideally, figure panels should include only measurements that are directly comparable to each other and obtained with the same assay.
- plots include clearly labeled error bars for independent experiments and sample sizes. Unless justified, error bars should not be shown for technical
- if n<5, the individual data points from each experiment should be plotted. Any statistical test employed should be justified.
- Source Data should be included to report the data underlying figures according to the guidelines set out in the authorship guidelines on Data

2. Captions
Each figure caption should contain the following information, for each panel where they are relevant:
- a specification of the experimental system investigated (eg cell line, species name).
- the assay(s) and method(s) used to carry out the reported observations and measurements.
- an explicit mention of the biological and chemical entity(ies) that are being measured.
- an explicit mention of the biological and chemical entity(ies) that are altered/varied/perturbed in a controlled manner.
- the exact sample size (n) for each experimental group/condition, given as a number, not a range;
- a description of the sample collection allowing the reader to understand whether the samples represent technical or biological replicates (including how many animals, litters, cultures, etc.).
- a statement of how many times the experiment shown was independently replicated in the laboratory.
- definitions of statistical methods and measures:
  - common tests, such as t-test (please specify whether paired vs. unpaired), simple χ2 tests, Wilcoxon and Mann-Whitney tests, can be unambiguously identified by name only, but more complex techniques should be described in the methods section;
  - are tests one-sided or two-sided?
  - are there adjustments for multiple comparisons?
  - exact statistical test results, e.g., P values = x but not P values < x;
  - definition of 'center values' as median or average;
  - definition of error bars as s.d. or s.e.m.

Please complete ALL of the questions below.
Select "Not Applicable" only when the requested information is not relevant for your study.

Materials

Newly Created Materials	Information included in the manuscript?	In which section is the information available? (Reagents and Tools Table, Materials and Methods, Figures, Data Availability Section)
New materials and reagents need to be available; do any restrictions apply?	Not Applicable	

Antibodies	Information included in the manuscript?	In which section is the information available? (Reagents and Tools Table, Materials and Methods, Figures, Data Availability Section)
For **antibodies** provide the following information:<br>- Commercial antibodies: RRID (if possible) or supplier name, catalogue number and or/clone number<br>- Non-commercial: RRID or citation	Yes	Materials and Methods

DNA and RNA sequences	Information included in the manuscript?	In which section is the information available? (Reagents and Tools Table, Materials and Methods, Figures, Data Availability Section)
**Short novel DNA or RNA including primers, probes**: provide the sequences.	Yes	Materials and Methods

Cell materials	Information included in the manuscript?	In which section is the information available? (Reagents and Tools Table, Materials and Methods, Figures, Data Availability Section)
**Cell lines:** Provide species information, strain. Provide accession number in repository **OR** supplier name, catalog number, clone number, and/**OR** RRID.	Not Applicable	
**Primary cultures:** Provide species, strain, sex of origin, genetic modification status.	Yes	Materials and Methods
Report if the cell lines were recently **authenticated** (e.g., by STR profiling) and tested for mycoplasma contamination.	Not Applicable	

Experimental animals	Information included in the manuscript?	In which section is the information available? (Reagents and Tools Table, Materials and Methods, Figures, Data Availability Section)
**Laboratory animals or Model organisms:** Provide species, strain, sex, age, genetic modification status. Provide accession number in repository **OR** supplier name, catalog number, clone number, **OR** RRID.	Yes	Materials and Methods
**Animal observed in or captured from the field:** Provide species, sex, and age where possible.	Not Applicable	
Please detail **housing and husbandry conditions**.	Yes	Materials and Methods

Plants and microbes	Information included in the manuscript?	In which section is the information available? (Reagents and Tools Table, Materials and Methods, Figures, Data Availability Section)
**Plants:** provide species and strain, ecotype and cultivar where relevant, unique accession number if available, and source (including location for collected wild specimens).	Not Applicable	
**Microbes:** provide species and strain, unique accession number if available, and source.	Yes	Materials and Methods

Human research participants	Information included in the manuscript?	In which section is the information available? (Reagents and Tools Table, Materials and Methods, Figures, Data Availability Section)
If collected and within the bounds of privacy constraints report on age, sex and gender or ethnicity for all study participants.	Not Applicable	

Core facilities	Information included in the manuscript?	In which section is the information available? (Reagents and Tools Table, Materials and Methods, Figures, Data Availability Section)
If your work benefited from core facilities, was their service mentioned in the acknowledgments section?	Yes	Acknowledgments

Design

Study protocol	Information included in the manuscript?	In which section is the information available? (Reagents and Tools Table, Materials and Methods, Figures, Data Availability Section)

If study protocol has been **pre-registered, provide DOI in the manuscript**. For clinical trials, provide the trial registration number **OR** cite DOI.	Not Applicable	
Report the **clinical trial registration number** (at ClinicalTrials.gov or equivalent), where applicable.	Not Applicable	

Laboratory protocol	Information included in the manuscript?	In which section is the information available? (Reagents and Tools Table, Materials and Methods, Figures, Data Availability Section)
Provide DOI OR other citation details if **external detailed step-by-step protocols** are available.	Not Applicable	

Experimental study design and statistics	Information included in the manuscript?	In which section is the information available? (Reagents and Tools Table, Materials and Methods, Figures, Data Availability Section)
Include a statement about **sample size** estimate even if no statistical methods were used.	Yes	Materials and Methods - Statistical analysis
Were any steps taken to minimize the effects of subjective bias when allocating animals/samples to treatment (e.g. **randomization procedure**)? If yes, have they been described?	Yes	Materials and Methods - Statistical analysis
Include a statement about **blinding** even if no blinding was done.	Yes	Materials and Methods - Statistical analysis
Describe **inclusion/exclusion criteria** if samples or animals were excluded from the analysis. Were the criteria pre-established?<br><br>If sample or data points were omitted from analysis, report if this was due to attrition or intentional exclusion and provide justification.	Yes	Materials and Methods - Statistical analysis
For every figure, are **statistical tests** justified as appropriate? Do the data meet the assumptions of the tests (e.g., normal distribution)? Describe any methods used to assess it. Is there an estimate of variation within each group of data? Is the variance similar between the groups that are being statistically compared?	Yes	Materials and Methods - Statistical analysis

Sample definition and in-laboratory replication	Information included in the manuscript?	In which section is the information available? (Reagents and Tools Table, Materials and Methods, Figures, Data Availability Section)
In the figure legends: state number of times the experiment was **replicated** in laboratory.	Yes	Figure legends
In the figure legends: define whether data describe **technical or biological replicates**.	Yes	Figure legends

Ethics

Ethics	Information included in the manuscript?	In which section is the information available? (Reagents and Tools Table, Materials and Methods, Figures, Data Availability Section)
Studies involving **human participants**: State details of **authority granting ethics approval** (IRB or equivalent committee(s), provide reference number for approval.	Not Applicable	
Studies involving **human participants**: Include a statement confirming that **informed consent** was obtained from all subjects and that the experiments conformed to the principles set out in the WMA Declaration of Helsinki and the Department of Health and Human Services Belmont Report.	Not Applicable	
Studies involving **human participants:** For publication of **patient photos**, include a statement confirming that consent to publish was obtained.	Not Applicable	
Studies involving experimental **animals**: State details of **authority granting ethics approval** (IRB or equivalent committee(s), provide reference number for approval. Include a statement of compliance with ethical regulations.	Yes	Materials and Methods - Mice
Studies involving **specimen and field samples:** State if relevant **permits** obtained, provide details of authority approving study; if none were required, explain why.	Not Applicable	

Dual Use Research of Concern (DURC)	Information included in the manuscript?	In which section is the information available? (Reagents and Tools Table, Materials and Methods, Figures, Data Availability Section)
Could your study fall under dual use research restrictions? Please check biosecurity documents and list of **select agents and toxins** (CDC): https://www.selectagents.gov/sat/list.htm	Not Applicable	
If you used a select agent, is the security level of the lab appropriate and reported in the manuscript?	Not Applicable	
If a study is subject to dual use research of concern regulations, is the name of the **authority granting approval and reference number** for the regulatory approval provided in the manuscript?	Not Applicable	

Reporting

The MDAR framework recommends adoption of discipline-specific guidelines, established and endorsed through community initiatives. Journals have their own policy about requiring specific guidelines and recommendations to complement MDAR.

Adherence to community standards	Information included in the manuscript?	In which section is the information available? (Reagents and Tools Table, Materials and Methods, Figures, Data Availability Section)
State if relevant guidelines or checklists (e.g., **ICMJE, MIBBI, ARRIVE, PRISMA**) have been followed or provided.	Not Applicable	
For **tumor marker prognostic studies,** we recommend that you follow the **REMARK** reporting guidelines (see link list at top right). See author guidelines, under 'Reporting Guidelines'. Please confirm you have followed these guidelines.	Not Applicable	
For **phase II and III randomized controlled trials**, please refer to the **CONSORT** flow diagram (see link list at top right) and submit the CONSORT checklist (see link list at top right) with your submission. See author guidelines, under 'Reporting Guidelines'. Please confirm you have submitted this list.	Not Applicable	

Data Availability

Data availability	Information included in the manuscript?	In which section is the information available? (Reagents and Tools Table, Materials and Methods, Figures, Data Availability Section)
Have **primary datasets** been deposited according to the journal's guidelines (see 'Data Deposition' section) and the respective accession numbers provided in the Data Availability Section?	Yes	Materials and Methods
Were **human clinical and genomic datasets** deposited in a public access-controlled repository in accordance to ethical obligations to the patients and to the applicable consent agreement?	Not Applicable	
Are **computational models** that are central and integral to a study available without restrictions in a machine-readable form? Were the relevant accession numbers or links provided?	Not Applicable	
If publicly available data were reused, provide the respective **data citations in the reference list**.	Not Applicable	
